# Structure–Property Relationships in Metakaolin Geopolymers Modified with Shell-Derived Calcium Particles for Multifunctional Wastewater Treatment

**DOI:** 10.3390/polym18162005

**Published:** 2026-08-17

**Authors:** Adriana-Gabriela Schiopu, Mihai Oproescu, Paul Mereuță, Sorin Georgian Moga, Ecaterina Magdalena Modan, Miruna-Adriana Ioța, Alexandru Berevoianu, Ștefan Mira, Marian-Cătălin Ducu, Elena Andreea Vijan, Daniela Istrate, Yasmin Loriana Teodora Grigore

**Affiliations:** 1 Doctoral School Materials Science and Engineering, National University of Science and Technology Politehnica Bucharest, Splaiul Independentei No. 313, Sector 6, 060042 Bucharest, Romania; gabriela.schiopu@upb.ro (A.-G.S.); iota.miruna@imnr.ro (M.-A.I.); stefan.mira@stud.sed.upb.ro (Ș.M.); elena_andreea.vijan@stud.sim.upb.ro (E.A.V.); daniela.calin@stud.sefi.upb.ro (D.I.); 2 Faculty of Mechanics and Technology, Pitesti University Centre, National University of Science and Technology Politehnica Bucharest, 110040 Pitesti, Romania; marian_catalin.ducu@upb.ro; 3 Faculty of Electronics, Communication and Computers, Pitesti University Centre, National University of Science and Technology Politehnica Bucharest, 110040 Pitesti, Romania; 4 Horia Hulubei National Institute for R&D in Physics and Nuclear Engineering (IFIN-HH), 077125 Magurele, Romania; paul.mereuta@nipne.ro (P.M.); alexandru.berevoianu@nipne.ro (A.B.); yasmin.grigore@nipne.ro (Y.L.T.G.); 5 Regional Center of Research & Development for Materials, Processes and Innovative Products Dedicated to the Automotive Industry (CRCD-AUTO), Pitesti University Centre, National University of Science and Technology Politehnica Bucharest, 060042 Bucharest, Romania; sorin_georgian.moga@upb.ro (S.G.M.); ecaterina.modan@upb.ro (E.M.M.); 6 National R&D Institute for Non-Ferrous and Rare Metals IMNR, 077145 Pantelimon, Romania; 7 Faculty of Physics, University of Bucharest Magurele, 077125 Magurele, Romania

**Keywords:** biogenic modified geopolymers, wastewater treatment, adsorption efficiency, preliminary antibacterial screening

## Abstract

The sustainable valorization of marine shell waste as functional additives for geopolymer materials represents a promising strategy for developing multifunctional materials for environmental remediation. In this study, metakaolin-based geopolymers were modified with calcium-rich particles obtained by calcination of five marine shell species (*Chamelea gallina*, *Mya arenaria*, *Mytilus edulis*, *Pecten maximus*, and *Rapana venosa*) under identical synthesis conditions to evaluate the influence of shell mineralogy on the structural, textural, adsorption, and antibacterial properties of the resulting composites. The materials were comprehensively characterized by Fourier transform infrared spectroscopy in attenuated total reflectance (ATR-FTIR), X-ray diffraction (XRD), scanning electron microscopy (SEM), and nitrogen adsorption–desorption (BET/BJH) analyses. Functional performance was assessed through methylene blue (MB) adsorption experiments, adsorption kinetic modeling, and antibacterial tests against *Escherichia coli* (*E. coli*). ATR-FTIR and XRD analyses confirmed the formation of a stable amorphous geopolymer network containing residual crystalline phases together with shell-derived calcium carbonate, predominantly as calcite or aragonite depending on shell origin. The incorporation of shell-derived particles modified the pore architecture of the geopolymers. GP-SJ exhibited the highest BET specific surface area (94.30 m^2^ g^−1^) and the most developed mesoporous structure. Among the investigated formulations, GP-RP showed the most favorable overall combination of functional properties under the investigated conditions, exhibiting the highest methylene blue removal efficiency (63.97%) and experimental adsorption capacity at 160 min (9.60 mg g^−1^), together with a comparatively high reduction in recoverable *E. coli* colonies during preliminary antibacterial screening. The combined structural and functional analyses demonstrate that the environmental performance of shell-modified geopolymers cannot be predicted from a single parameter such as BET surface area or calcium content alone, but results from the synergistic interaction between mineralogical composition, particle dispersion, pore accessibility, and matrix compactness. Under the investigated conditions, these findings provide evidence for proposed structure–property correlations under the investigated conditions and suggests that shell-derived calcium particles act as microstructural regulators of geopolymer matrices, providing a basis for the further development of sustainable multifunctional materials for simultaneous dye removal and bacterial reduction in wastewater treatment.

## 1. Introduction

Water pollution caused by synthetic dyes represents one of the major environmental challenges associated with rapid industrialization. Large quantities of colored effluents generated by textile, paper, leather, pharmaceutical, and printing industries are continuously discharged into aquatic environments, where they reduce light penetration, inhibit photosynthesis, and may exhibit toxic, mutagenic, or carcinogenic effects on living organisms. Among the numerous organic dyes, methylene blue (MB) is widely employed as a model cationic contaminant because of its high chemical stability, strong coloration, and persistence in aqueous environments [1]. Conventional wastewater treatment technologies, including coagulation–flocculation, membrane filtration, advanced oxidation processes, and biological degradation, often suffer from high operational costs, limited efficiency, or the generation of secondary pollutants. Consequently, adsorption has emerged as one of the most attractive technologies for dye removal due to its simplicity, high efficiency, low energy demand, and ease of operation [2].

Geopolymers have attracted considerable attention as environmentally friendly inorganic polymers produced through the alkaline activation of aluminosilicate precursors [3,4,5,6,7]. Their three-dimensional aluminosilicate framework, high chemical stability, tunable pore structure, and ion-exchange capability make them promising candidates for environmental remediation applications [8]. Among the various aluminosilicate precursors, metakaolin has become one of the most extensively investigated owing to its high purity, controlled chemical composition, and excellent reactivity under alkaline activation. Metakaolin-based geopolymers generally exhibit a homogeneous aluminosilicate network, good mechanical integrity, and adjustable pore characteristics, making them suitable for applications ranging from construction materials to adsorbents and functional environmental materials [9,10,11,12,13].

Compared with conventional adsorbents such as activated carbon, geopolymers can be synthesized from abundant natural resources or industrial by-products while exhibiting lower production costs and reduced carbon emissions [9,10]. Recent studies have demonstrated their ability to remove heavy metals, radionuclides, pharmaceuticals, and organic dyes from contaminated waters, highlighting the important role of surface chemistry, pore accessibility, and microstructural organization in adsorption performance [5,11,12].

In parallel with the increasing interest in geopolymers, the valorization of marine shell waste has become an important topic within the framework of the circular economy. Millions of tons of seashell residues generated by the seafood industry are commonly disposed of in landfills despite their high calcium carbonate content [13]. Following calcination, these wastes can provide calcium-rich materials capable of modifying the physicochemical properties of cementitious and geopolymeric matrices [14]. Previous studies have reported that shell-derived calcium compounds may accelerate geopolymerization, refine pore structure, improve mechanical properties, and enhance surface reactivity [15]. Nevertheless, the influence of different biological shell sources on the functional performance of geopolymers remains insufficiently understood. Although numerous investigations have reported the incorporation of calcium carbonate or shell-derived powders into geopolymers, most studies have focused primarily on mechanical performance or general microstructural characterization [16]. Comparatively few studies have systematically correlated the mineralogical characteristics of different shell species with pore architecture, adsorption kinetics, and antibacterial behavior under identical synthesis conditions [17,18]. Furthermore, the combined evaluation of adsorption efficiency and antimicrobial activity remains scarce, despite the growing demand for multifunctional materials capable of simultaneously removing organic contaminants and reducing microbial loads during wastewater treatment. Consequently, the relationships between shell mineralogy, geopolymer microstructure, adsorption kinetics, and reduction in recoverable *E. coli* colonies remain insufficiently established.

Incorporating CaCO_3_ (calcium carbonate/limestone) into geopolymers is an increasingly popular strategy [19]. When used in either nanoparticle (nano-CaCO_3_) or micro-filler (limestone powder) formats, it acts as a highly effective filler, accelerator, and reinforcing agent that improves the mechanical and microstructural properties of the geopolymer matrix [20,21].

To address these limitations, the present study investigates metakaolin-based geopolymers modified with calcium-rich particles obtained from five different calcined marine shell species (*Chamelea gallina*, *Mya arenaria*, *Mytilus edulis*, *Pecten maximus*, and *Rapana venosa*). Unlike previous studies that generally examine a single shell source, all additives were incorporated using the same geopolymer formulation and identical processing conditions, enabling a direct comparison of the influence of shell origin on the structural and functional properties of the resulting composites [22,23,24,25,26]. Comprehensive characterization combining ATR-FTIR, XRD, BET/BJH, SEM, EDX, antibacterial assays, methylene blue adsorption experiments, and adsorption kinetic modeling was performed to identify qualitative structure–property correlations. The main contributions of this study are: (i) the systematic comparison of five different shell-derived calcium additives under identical geopolymer synthesis conditions; (ii) the identification of qualitative correlations between structural characteristics and functional responses by comparing FTIR, XRD, SEM, EDX, BET, adsorption kinetics, and antibacterial activity; (iii) the identification of the most favorable formulation for simultaneous dye adsorption and bacterial inhibition under the investigated conditions; and (iv) the demonstration of the potential of shell waste valorization for sustainable wastewater depollution applications.

## 2. Materials and Methods

### 2.1. Geopolymers Preparation

To obtain the geopolymer composite materials, 19.5 g of metakaolin was first dry-mixed with 0.5 g of each calcium-based particles from calcined shells. The biogenic particles were produced by calcination of five different marine shell species: *Chamelea gallina*, *Mya arenaria*, *Mytilus edulis*, *Pecten maximus*, and *Rapana venosa*, at 900 °C for 2 h. The marine shell powders were calcined at 900 °C to ensure the effective decomposition of calcium carbonate into reactive calcium oxide, providing calcium-rich phases capable of modifying the geopolymerization process and the resulting microstructure, as presented in [27].

The metakaolin used as the aluminosilicate precursor for geopolymer synthesis was obtained by calcining kaolin (Naturall Home, Salonta, Romania) at 750 °C for 2 h in a Mikrotest MKF-05 muffle furnace (Ankara, Türkiye). Kaolin was calcined at 750 °C, which is widely recognized as the optimum temperature range for the dehydroxylation of kaolinite. Higher calcination temperatures may promote recrystallization and reduce its chemical reactivity. The calcination process induced the formation of an amorphous aluminosilicate phase with high reactivity, favorable for alkaline activation and geopolymerization processes. Elemental composition analysis of metakaolin, performed by energy-dispersive X-ray spectroscopy (EDX), revealed the presence of 48.5 at. % O, 18.7 at. % C, 15.8 at. % Si, 15.6% at. Al, 1.0 at. % K and 0.5 at. % Fe as presented in a previous study [1].

The alkaline activating solution was prepared by combining two concentrated alkaline solutions consisting of 3.6 g of 8 M NaOH and 3.6 g of 8 M KOH. The mixed Na^+^/K^+^ alkaline system was selected to optimize the dissolution of the aluminosilicate precursor and enhance the kinetics of the geopolymerization reactions. The simultaneous presence of sodium and potassium cations promotes the structural reorganization of dissolved silicate and aluminate species, contributing to the development of a highly polycondensed geopolymeric network with improved structural stability.

Subsequently, the alkaline activating solution was gradually added to the solid mixture under continuous mechanical stirring to ensure uniform particle dispersion and initiate the alkaline dissolution processes. Thereafter, 16.5 g of sodium silicate (Na_2_SiO_3_) was incorporated as an additional source of reactive silicate species and as a promoter of polycondensation reactions.

The homogenization process continued until a uniform paste free of visible agglomerates was obtained, exhibiting a consistency suitable for casting and compaction into molds.

The resulting geopolymer pastes were cast into cylindrical molds with a diameter of 4 cm. Immediately after casting, the molds were tightly sealed with polyethylene film to minimize precursor evaporation and moisture loss. The sealed specimens were thermally cured at 60 °C for 24 h in a temperature-controlled oven (Biobase Bioland Co., Ltd., Jinan, China). After thermal curing, the specimens were not demolded and the polyethylene enclosure was not removed. Instead, they remained sealed in their original molds and were maintained at ambient laboratory temperature (23 ± 2 °C) for 28 days. The relative humidity of the surrounding laboratory atmosphere was measured at approximately 10%; however, because the specimens remained sealed throughout this period, this value represents the external ambient humidity and not the relative humidity within the sealed molds, which was not directly measured. All formulations were prepared, thermally cured, and stored concurrently under the same nominal conditions. This sealed curing protocol was selected to minimize uncontrolled moisture loss, ensure a consistent curing history across all formulations, and maintain methodological continuity with the procedure previously employed by the authors in [1].

**Figure 1:**
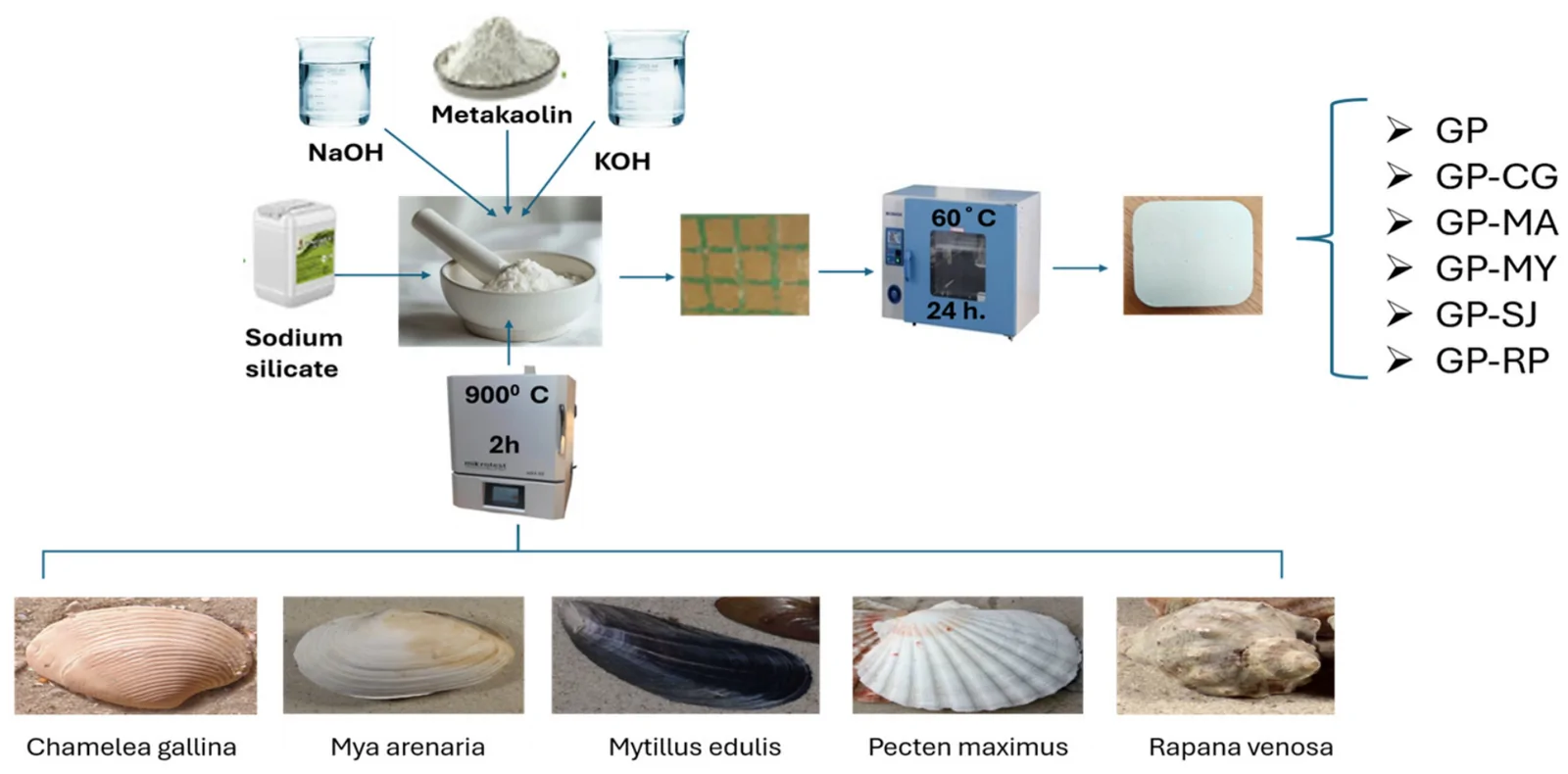
Elaboration of biogenic calcium-based modified geopolymer materials.

During this process, the dissolved aluminosilicate species reacted with soluble silicates in the highly alkaline medium, resulting in the formation of a (Na,K)-A-S-H gel, which is primarily responsible for the development of the structural and functional properties of geopolymer materials. The samples investigated were named GP (geopolymer) and geopolymeric composites modified with shell-derived powders from *Chamelea gallina* (GP-CG), *Mya arenaria* (GP-MA), *Mytilus edulis* (GP-MY), *Pecten maximus* (GP-SJ), and *Rapana venosa* (GP-RP). The schema of elaboration process is presented in Figure 1.

### 2.2. Characterisation Methods

Fourier transform infrared (FTIR) spectroscopy in attenuated total reflectance (ATR) mode was employed to characterize the chemical structure and identify the main functional groups of the synthesized geopolymers incorporating calcium-based particles derived from calcined shell waste. Spectra were recorded over the 4000–350 cm^−1^ wavenumber range using a Bruker Tensor 27 spectrometer (Bruker Optik GmbH, Ettlingen, Germany) equipped with a diamond ATR crystal. All measurements were performed at a spectral resolution of 4 cm^−1^, with 32 scans accumulated and averaged for each sample. The structural characterization was performed by X-ray diffraction (XRD), a widely used technique for the identification of crystalline phases and the evaluation of structural modifications induced by calcium-based additives. The XRD analysis enabled the investigation of both residual crystalline components originating from the aluminosilicate precursor and carbonate phases originating from the shell-derived additives and present after alkaline activation. X-ray diffraction measurements were carried out using CuKα radiation operating at 45 kV and 40 mA. Diffractograms were recorded over a 2θ angular range from 10° to 85°, employing a scanning rate of 2° min^−1^ and a step size of 0.05° to ensure accurate identification of the crystalline phases. Phase identification was performed by comparing the positions and relative intensities of the experimental diffraction peaks with the crystallographic database PDF-5+ 2025.

Prior to analysis, all specimens were finely ground and homogenized to obtain uniform powders, thereby minimizing preferred orientation effects and improving the quality and reproducibility of the recorded diffraction patterns.

The textural properties of the synthesized geopolymer materials were characterized by nitrogen adsorption–desorption analysis at 77 K. The specific surface area was determined using the Brunauer–Emmett–Teller (BET) method, while the pore size distribution and average mesopore diameter were obtained from the desorption branch of the isotherms according to the Barrett–Joyner–Halenda (BJH) model. The t-plot method was employed to estimate the external surface area and micropore contribution. The integration of these complementary approaches provided a detailed evaluation of the porous architecture and surface characteristics of the geopolymer systems.

The mesoporosity index (I_m_) was estimated using the following relationship:

(1) $$I_{m}=\frac{\mathrm{BJH}\mathrm{ads}.\mathrm{Pore}\mathrm{Volume}}{\mathrm{BET}}$$

where:

BJH ads. Pore Volume is the BJH pore volume determined from the adsorption branch (cm^3^ g^−1^);

BET is the BET specific surface area (m^2^ g^−1^).

The mesoporosity index provides an estimate of the relationship between pore volume and available surface area and can therefore be used as an indicator of mesoporous network development.

In geopolymeric materials, this parameter is particularly useful for comparing the effects of different additives on pore structure evolution. By simultaneously considering pore volume and specific surface area, the mesoporosity index provides complementary information regarding pore accessibility, pore size distribution, and the efficiency of pore network formation. Consequently, it represents a valuable indicator for assessing the influence of shell-derived calcium-rich additives on the textural properties and microstructural organization of geopolymer composites.

Scanning electron microscopy (SEM) (JEOL Ltd., Tokyo, Japan) was employed to investigate the morphology and the interactions between the aluminosilicate matrix and biogenic calcium-based particles from calcined shells incorporated into the geopolymer composites. Micrographs were acquired from two distinct regions of each sample at magnifications of 300× and 1000×, under high vacuum, while selected areas were additionally examined at 10,000× magnification to reveal finer microstructural details. This multiscale approach enabled the evaluation of both the overall organization of the geopolymeric matrix and the local morphological characteristics associated with the incorporation of biogenic additives.

Prior to analysis, the specimens were dried to remove residual moisture and mounted on conductive carbon tape holders.

The preliminary antibacterial response of the developed geopolymer composites was investigated using the reference bacterial strain *Escherichia coli* WDCM 00013 (VT 000132), supplied by Merck (Darmstadt, Germany), characterized by a specific Gram-negative cell wall architecture. *E. coli* was selected as a representative Gram-negative microorganism for the preliminary evaluation of the antibacterial behavior of the developed geopolymer composites. The reduction in recoverable *E. coli colonies* was determined using the membrane filtration method followed by colony-forming unit (CFU) enumeration. Prior to testing, decimal serial dilutions were prepared from the stock cultures to obtain countable bacterial populations. Chromocult^®^ Coliform Agar (CCA) was used as the selective chromogenic medium for the isolation and enumeration of *E. coli*. Artificial bacterial suspensions containing approximately 10^2^ CFU mL^−1^ of E. coli were prepared for the antibacterial assays. For each experimental condition, three sample categories were established: positive control: 9 mL sterile distilled water and 1 mL bacterial suspension; negative control: 10 mL sterile distilled water and test samples: 9 mL sterile distilled water, 1 mL bacterial suspension, and 2 mg of geopolymer powder. The suspensions were homogenized using a Vortex V-1 Plus mixer (Biosan SIA, Latvia, Riga) to ensure uniform contact between the bacterial cells and the geopolymer particles. The antibacterial activity was evaluated after 30 min, 4 h, and 24 h of contact. During the contact period, the suspensions were maintained in the dark at 25 °C. At each sampling time, 1 mL of suspension was filtered through a sterile membrane filter, which was subsequently placed onto Chromocult^®^ Coliform Agar plates. The plates were incubated at 37 °C for 24 h, after which the characteristic blue colonies of *E. coli* were counted and expressed as colony-forming units (CFU).

The reduction efficiency of recoverable *E. coli* colonies (RE, %) of the investigated materials was calculated according to Equation (2):

(2) $$RE(\%)=\frac{N_{M^{+}}-N_{\mathrm{sample}}}{N_{M^{+}}}\times 100$$

where $N_{M^{+}}$ represents the number of colony-forming units (CFU/mL) measured for the positive control, and $N_{\mathrm{sample}}$ is the number of colony-forming units (CFU/mL) determined for the corresponding geopolymer sample after the same contact time. An antibacterial efficiency of 100% indicates complete bacterial inhibition, whereas 0% corresponds to the absence of antibacterial activity.

The adsorption performance of the synthesized geopolymer composites was evaluated using methylene blue (MB) as a model cationic dye. An aqueous MB solution with an initial concentration of 15 mg L^−1^ (15 ppm) was prepared using deionized water. For each adsorption experiment, 0.1 g of geopolymer powder was dispersed in 100 mL of the MB solution and continuously stirred under dark conditions at room temperature (23 ± 2 °C) to eliminate any contribution from photocatalytic reactions. The suspensions were magnetically stirred at 300 rpm. At predetermined contact times (20, 40, 60, 80, 100, 120, 140 and 160 min), aliquots of the suspension were withdrawn. Prior to UV–Vis analysis, the solid particles were filtered to avoid light scattering effects caused by suspended geopolymer particles. The residual concentration of methylene blue was determined using an Ocean Optics USB2000+ UV–Vis spectrometer (Ocean Optics, Inc., Dunedin, FL, USA) by monitoring the characteristic absorption maximum of MB at 664 nm.

The adsorption efficiency (η) was calculated according to Equation (3):

(3) $$\eta (\%)=\left(1-\frac{A_{t}}{A_{0}}\right)\times 100$$

where $A_{0}$ and $A_{t}$ represent the absorbance of the initial MB solution and the absorbance measured at contact time t, respectively. According to the Beer–Lambert law, the absorbance ratio ($A_{t}/A_{0}$) is directly proportional to the concentration ratio ($C_{t}/C_{0}$).

In addition to the removal efficiency, the adsorption capacity of the investigated geopolymer materials was calculated using the experimental methylene blue concentration measured after 160 min of adsorption according to Equation (4):

(4) $$q_{160}=\frac{\left(C_{0}-C_{160}\right)V}{m}$$

where $C_{0}$ (mg L^−1^) is the initial methylene blue concentration, $C_{160}$ (mg L^−1^) is the residual MB concentration measured after 160 min. of adsorption, V(L) is the solution volume, and m(g) is the mass of the geopolymer adsorbent. Since all adsorption experiments were performed under identical conditions ($C_{0}=15$ mg L^−1^, V=100 mL and m=0.1 g), the calculated adsorption capacities enabled a direct comparison of the adsorption performances of the investigated geopolymer formulations.

The quantitative BET/BJH, methylene blue adsorption, kinetic, and antibacterial results reported in this study were obtained from a single independent experiment for each geopolymer formulation, using specimens collected from the same synthesis batch and correlated with the structural, morphological and textural properties of the synthesized geopolymer composites obtained from XRD and SEM.

## 3. Results

### 3.1. ATR-FTIR Analysis

**Figure 2:**
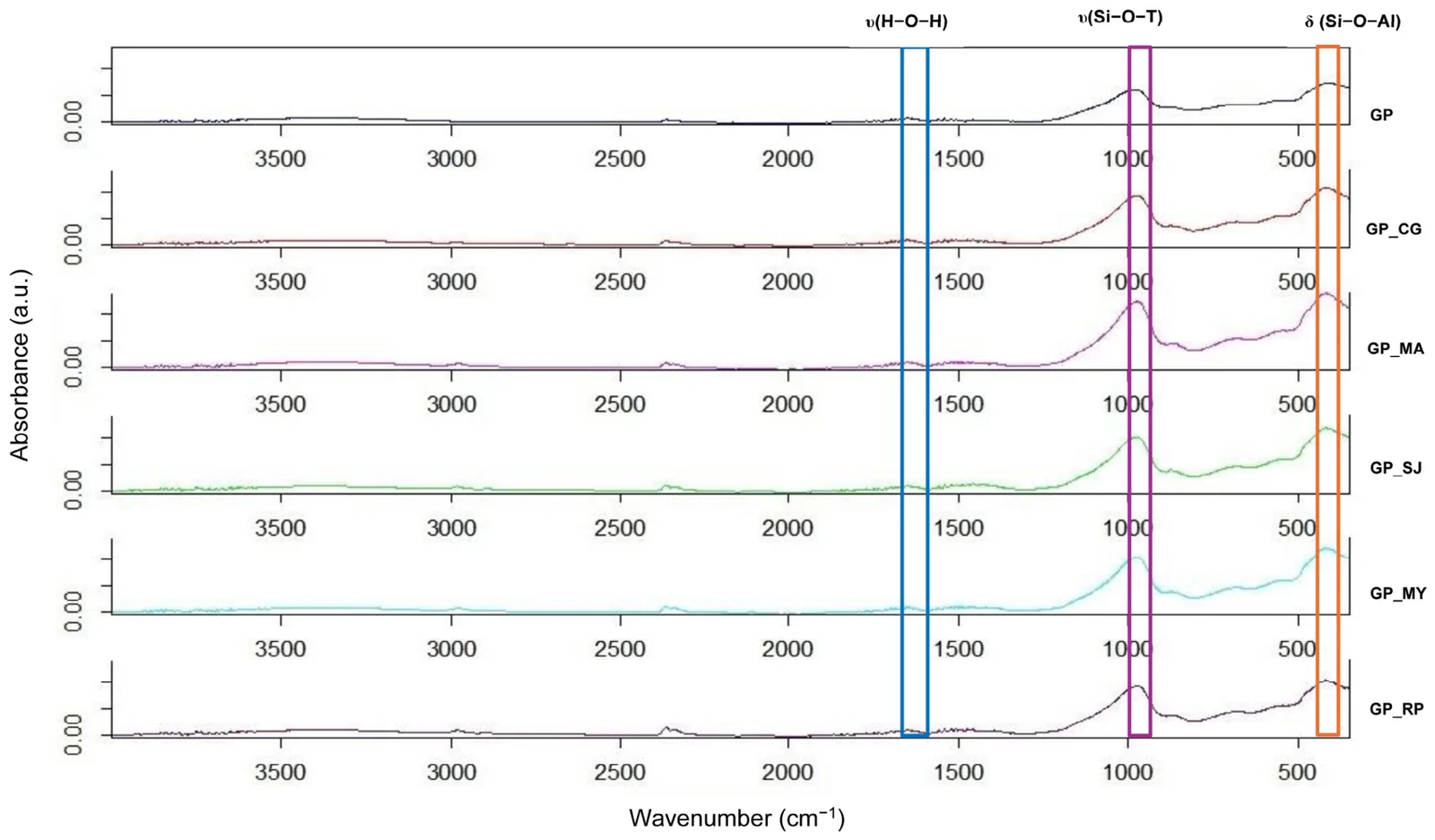
ATR-FTIR spectra of geopolymers.

The ATR-FTIR spectra of the reference geopolymer (GP) and the geopolymer composites incorporating biogenic Ca-based particles from calcined shells (GP-CG, GP-MA, GP-MY, GP-SJ, and GP-RP) are presented in Figure 2.

The overall similarity of the spectra demonstrates that the incorporation of shell-derived biogenic particles does not alter the fundamental aluminosilicate framework generated during geopolymerization. All specimens exhibit the characteristic vibrational bands associated with geopolymer materials, indicating that the alkaline activation process successfully produced an amorphous aluminosilicate network regardless of the type of calcium-rich additive.

The FTIR spectra of all investigated geopolymer samples exhibit the characteristic vibrational features of alkali-activated aluminosilicate materials. A weak absorption band observed near 1640 cm^−1^ is assigned to the H–O–H bending vibration of molecular water retained within the geopolymer matrix, indicating the presence of physically adsorbed and structurally bound water. Although the broad O–H stretching vibration expected in the 3200–3600 cm^−1^ region is only weakly resolved, its presence is consistent with the hydrated nature of the geopolymeric gel and agrees with previous reports on metakaolin-based geopolymers.

The dominant absorption band located between 950 and 1050 cm^−1^ corresponds to the asymmetric stretching vibration of Si–O–T (T = Si or Al) bonds and represents the characteristic fingerprint of the geopolymeric network. Minor variations in its intensity and position among the investigated formulations indicate that the incorporation of shell-derived calcium particles slightly modifies the local structural environment without altering the integrity of the geopolymeric framework.

Carbonate-related vibrations expected in the 1400–1470 cm^−1^ region are only weakly resolved because of their low intensity and partial overlap with neighboring absorption bands. Nevertheless, their presence is consistent with the XRD results, which confirmed crystalline calcium carbonate phases in the shell-modified geopolymers. This indicates that a fraction of the shell-derived calcium phases remains chemically stable after alkaline activation and curing.

The absorption band below 500 cm^−1^ is assigned to Si–O–Al and O–Si–O bending vibrations, reflecting the formation of the aluminosilicate framework characteristic of geopolymeric materials. The persistence of this band in all investigated samples further confirms that the addition of shell-derived particles does not disrupt the development of the geopolymeric network.

### 3.2. Structural Analysis

**Figure 3:**
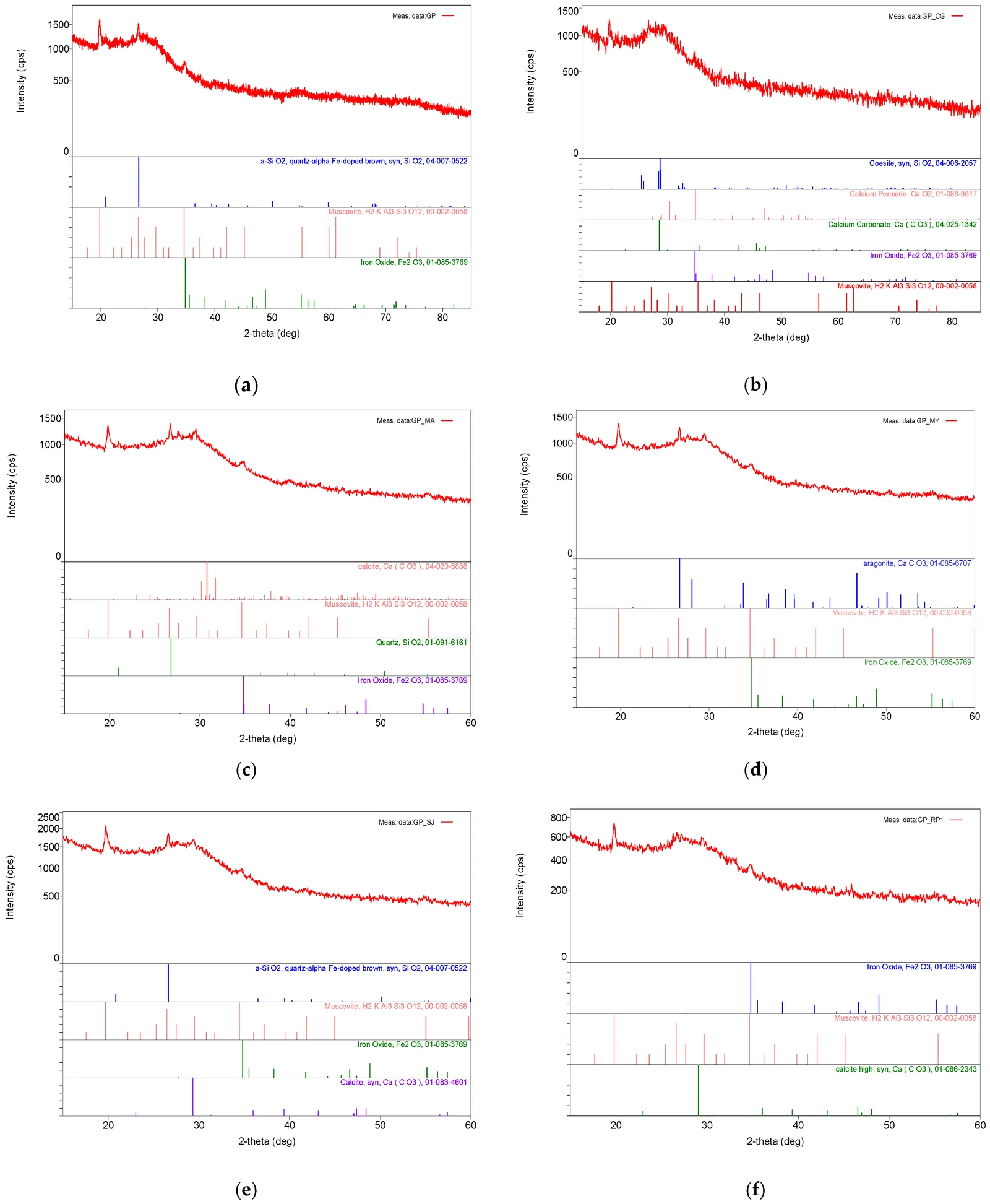
XRD pattern of (**a**) GP; (**b**) GP-CG; (**c**) GP-MA; (**d**) GP-MY; (**e**) GP-SJ; and (**f**) GP-RP.

The X-ray diffraction patterns of the reference geopolymer (GP) and the geopolymer composites modified with Ca-based particles from calcined shells are presented in Figure 3. The diffraction results demonstrate that alkaline activation preserves several crystalline phases inherited from the metakaolin precursor while simultaneously introducing additional calcium carbonate phases originating from the biogenic shell additives.

All specimens exhibit a broad diffuse halo between approximately 20° and 35° (2θ), characteristic of the predominantly amorphous geopolymer gel formed during alkaline activation. Superimposed on this amorphous background, several crystalline phases remain detectable, indicating that geopolymerization is only partial and that a fraction of the precursor minerals remain unreacted or only partially dissolved.

The XRD pattern of the reference geopolymer (Figure 3) is characterized by the presence of quartz (α-SiO_2_), muscovite, and hematite (Fe_2_O_3_). Quartz and muscovite originate commonly observed in metakaolin-based geopolymers. Hematite is attributed to iron-bearing mineral impurities inherited from the raw material. The coexistence of these residual crystalline phases with the amorphous geopolymer gel confirms the heterogeneous nature of the geopolymer matrix. The incorporation of shell-derived particles does not significantly modify the crystalline phases associated with the aluminosilicate precursor, as quartz, muscovite and Fe_2_O_3_ remain detectable in most samples. The principal mineralogical modification is the appearance of crystalline calcium carbonate phases originating from the calcined marine shells.

Three distinct mineralogical behaviors can be distinguished. The GP-MA, GP-SJ, and GP-RP samples are characterized by the predominance of calcite (CaCO_3_). The persistence of calcite after alkaline activation and 28 days of curing indicates that crystalline CaCO_3_ remains embedded within the geopolymer matrix. Consequently, these shell-derived particles behave as mineral fillers capable of modifying the microstructure.

In contrast, the GP-MY sample exhibits aragonite as the dominant calcium carbonate polymorph. Since aragonite is the original mineral phase of many marine shells, its preservation demonstrates that complete polymorphic transformation into calcite did not occur during calcination, alkaline activation, or curing. This result suggests a relatively high mineralogical stability of the Mytilus edulis-derived additive and indicates that the original biogenic structure is partially retained within the geopolymer composite.

The diffraction pattern of GP-CG also reveals the presence of crystalline calcium carbonate together with silica polymorphs and residual aluminosilicate phases. Several reflections are tentatively assigned CaO_2_ (ICDD PDF 01-088-9817). Its formation may be associated with the high reactivity of shell-derived calcium phases generated during calcination. During alkaline activation, residual CaO readily hydrates to Ca(OH)_2_, while the highly alkaline and oxygen-containing reaction environment may facilitate the formation or stabilization of minor peroxide-containing calcium species. Subsequent encapsulation within the geopolymer matrix may limit further hydration and carbonation, favoring the preservation of the detected phase. Although the exact reaction pathway cannot be established from the present results.

Importantly, the diffraction patterns indicate that the calcium-based shell-derived additives are incorporated without disrupting the formation of the amorphous geopolymer network. Instead, they coexist with the geopolymer gel as dispersed crystalline calcium carbonate particles. Depending on the biological origin of the shells, either calcite or aragonite is retained after geopolymerization. Calcite predominates in the GP-MA, GP-SJ, and GP-RP composites, whereas aragonite is preserved only in GP-MY, highlighting the influence of shell mineralogy on the final crystalline composition.

The combined FTIR and XRD analyses indicate that the final composites consist of an amorphous N-A-S-H matrix containing residual crystalline aluminosilicate minerals together with biogenic calcium carbonate phases.

### 3.3. Textural Characterization

The BET technique enables the assessment of surface characteristics that directly affect the reactivity, adsorption capacity, and overall performance of geopolymeric materials. The textural properties determined by BET and BJH analyses revealed significant differences in pore architecture and particle organization among the samples investigated, as presented in Table 1. All modified geopolymer composites exhibited average pore diameters within the mesoporous range (11–36 nm), confirming the predominance of mesoporosity in the synthesized materials.

The reference geopolymer (GP) exhibited the largest BJH pore diameters, reaching 35.20 nm during adsorption and 31.78 nm during desorption. This behavior is associated with a lower pore density and a relatively low specific surface area, suggesting the presence of a less developed porous network. In contrast, the GP-SJ and GP-CG samples displayed smaller average pore diameters accompanied by higher BET specific surface areas, indicating the formation of a finer and more homogeneous porous structure.

Among all investigated materials, GP-SJ exhibited the smallest BET equivalent particle size (26.51 nm) and the highest specific surface area (94.30 m^2^ g^−1^). These characteristics suggest an improved dispersion of calcium-based particles throughout the geopolymeric matrix and enhanced pore accessibility. The increased surface area may also reflect a more efficient interaction between the shell-derived additive and the aluminosilicate gel network, resulting in the development of a highly interconnected mesoporous structure. Conversely, the GP-MA and GP-MY samples showed larger equivalent particle sizes and lower specific surface areas.

**Figure 4:**
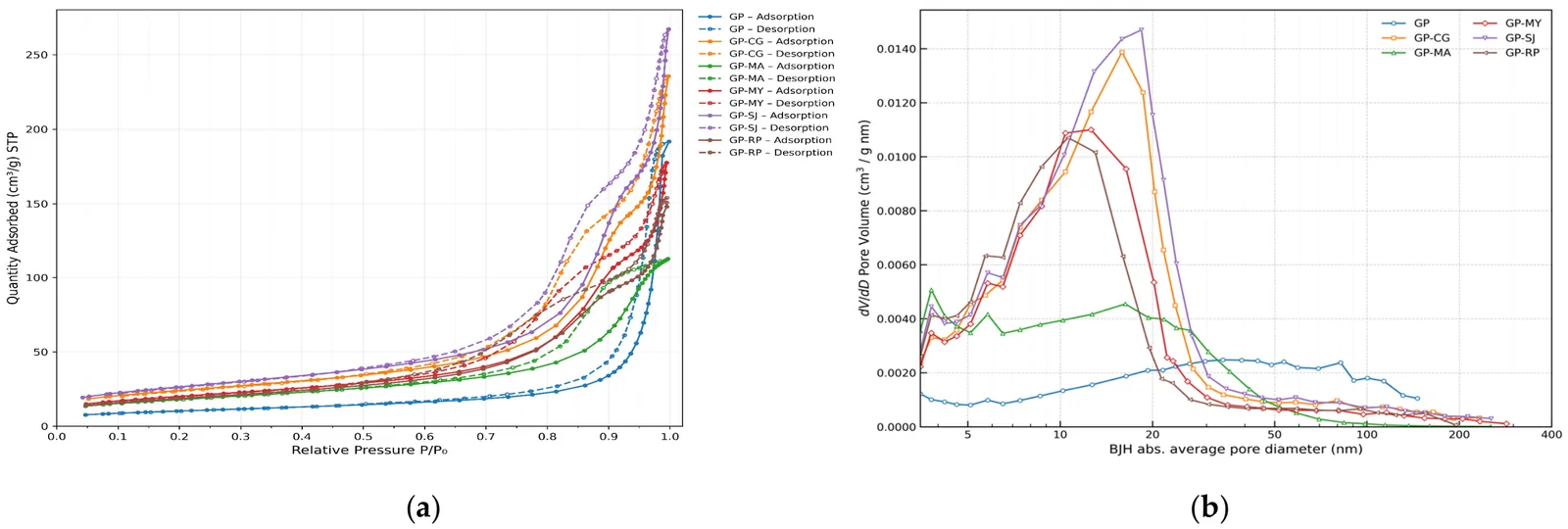
(**a**) Nitrogen adsorption–desorption isotherms of the investigated geopolymer samples; (**b**) BJH differential pore size distribution.

The nitrogen adsorption–desorption isotherms of the investigated geopolymer samples are presented in Figure 4a. All formulations exhibit a pronounced increase in nitrogen uptake at high relative pressures (P/P_0_ > 0.8), accompanied by a distinct adsorption–desorption hysteresis loop, indicating the predominance of mesoporosity. The modified geopolymer samples adsorbed larger amounts of nitrogen than the reference GP, demonstrating that the incorporation of shell-derived particles substantially altered the pore structure. Among the investigated formulations, GP-SJ exhibited the highest nitrogen uptake, followed by GP-CG, GP-MY, GP-RP, and GP-MA, in agreement with the BET surface area values. The observed hysteresis loops are characteristic of capillary condensation within mesopores, confirming that the investigated materials possess interconnected pore systems suitable for adsorption-related environmental applications.

The BJH pore size distribution curves (Figure 4b) confirm that all investigated geopolymers possess predominantly mesoporous structures. Compared with the reference GP, the shell-derived calcium modified geopolymers exhibit a greater concentration of mesopores, particularly within the 10–20 nm range, indicating that the incorporation of shell-derived particles effectively tailors the pore architecture. GP-SJ and GP-CG show the highest differential pore volumes, whereas GP-MA exhibits a broader and less pronounced distribution. These results are consistent with the BET analysis and demonstrate that the shell-derived additives influence the textural properties of the geopolymer matrix under the investigated conditions.

The variation in pore volume, presented in Table 2, highlights the significant influence of calcium-based particles derived from marine shells on the development of the geopolymer porous network. Compared with reference geopolymer (GP), all modified samples exhibited increased pore volume values, indicating that the incorporation of biogenic particles promoted the formation of a more accessible mesoporous structure.

The GP sample exhibited the lowest pore volume at adsorption (P/P_0_ = 0.95), reaching only 0.0939 cm^3^ g^−1^, which suggests a denser matrix with limited pore accessibility. In contrast, the GP-SJ sample displayed the highest pore volume values, reaching 0.2637 cm^3^ g^−1^ during adsorption and 0.2946 cm^3^ g^−1^ during desorption. These results demonstrate the development of a highly porous structure with enhanced pore accessibility and a well-developed mesoporous network, as presented in Table 2.

Similarly, the GP-CG sample exhibited elevated pore volumes ranging from 0.2312 to 0.2645 cm^3^ g^−1^, further confirming the beneficial effect of the shell-derived additive on mesopore generation and pore network expansion. Intermediate pore volume values were observed for the GP-MY and GP-RP samples, whereas GP-MA exhibited the lowest pore volume among the modified geopolymers. This behavior suggests partial pore blockage and a higher degree of particle aggregation, resulting in a reduction in the accessible pore space.

The higher cumulative BJH pore volume values observed for GP-SJ and GP-CG confirm the development of a well-defined mesoporous network, characterized by improved pore connectivity and accessibility. Conversely, the lower values obtained for GP-MA indicate a more compact microstructure with reduced pore development. These findings are consistent with the BET surface area results and support the conclusion that the effectiveness of shell-derived additives in enhancing the porous architecture of geopolymer composites strongly depends on particle dispersion, aggregation behavior, and the interaction between calcium-rich phases and the aluminosilicate gel matrix.

The calculated mesoporosity index values revealed significant differences in pore network organization among the investigated geopolymer samples, as presented in Table 3.

I_m_ is used here only as a comparative descriptor and should not be interpreted as an independent measure of pore uniformity, connectivity, or packing efficiency. Differences in I_m_ reflect variations in pore volume normalized to the available surface area rather than direct differences in pore uniformity or connectivity. Therefore, the I_m_ values were considered only in conjunction with the BET surface area, BJH pore volume, average pore diameter, and SEM observations when comparing the textural characteristics of the investigated materials.

The reference geopolymer (GP) exhibited the highest mesoporosity index (0.00771), despite possessing the lowest BET specific surface area. This behavior is consistent with pore structure dominated by a comparatively small number of larger mesopores relative to the available surface area, in agreement with the BJH pore-size-distribution data (Figure 4b). Consequently, the pore volume is concentrated in fewer mesopores, resulting in a higher ratio between pore volume and specific surface area.

In contrast, the Ca-based modified geopolymer composites exhibited lower mesoporosity index values, which is compatible with the development of a finer mesoporous network, as also indicated by the BJH pore-size-distribution curves (Figure 4b). The decrease in the index reflects a lower pore volume relative to the available surface area and may be associated with a larger contribution of smaller pores; however, the I_m_ ratio alone does not independently establish pore uniformity, distribution, or packing efficiency. These modifications are accompanied by a substantial increase in specific surface area while maintaining significant pore volumes.

Among the modified samples, GP-SJ and GP-CG exhibited a particularly favorable combination of textural parameters, including high BET surface areas, elevated pore volumes, and moderate mesoporosity index values. These characteristics are consistent with an optimized porous architecture consisting of numerous well-distributed mesopores, high pore accessibility, and a large active surface area. Such a structure is advantageous for applications requiring enhanced adsorption capacity, mass transport, and surface reactivity.

Conversely, GP-MA exhibited the lowest mesoporosity index (0.00263), consistent with a more restricted porous structure. This result suggests partial pore blockage and a higher degree of aggregation of calcium-rich particles, which may reduce pore accessibility and limit the development of the mesoporous network. The lower index value, together with the reduced pore volume and specific surface area observed for this sample, supports the hypothesis that particle agglomeration negatively affects pore formation and structural homogeneity.

The mesoporosity index analysis indicates that the incorporation of shell-derived biogenic powders influences pore architecture. The results demonstrate that the effectiveness of the additives depends not only on their chemical composition but also on their ability to disperse within the geopolymeric matrix and promote the formation of a highly accessible and uniformly distributed mesoporous network. I_m_ values were considered only in conjunction with the BET surface area, BJH pore volume, average pore diameter, and SEM observations when comparing the textural characteristics of the investigated metakaolin geopolymers modified with shell-derived calcium particles.

### 3.4. Morphological Analysis

Furthermore, the SEM observations provide complementary information to the XRD and BET results, facilitating a comprehensive understanding of the relationships between phase composition, pore architecture, and microstructural evolution in the synthesized geopolymer composites.

**Figure 5:**
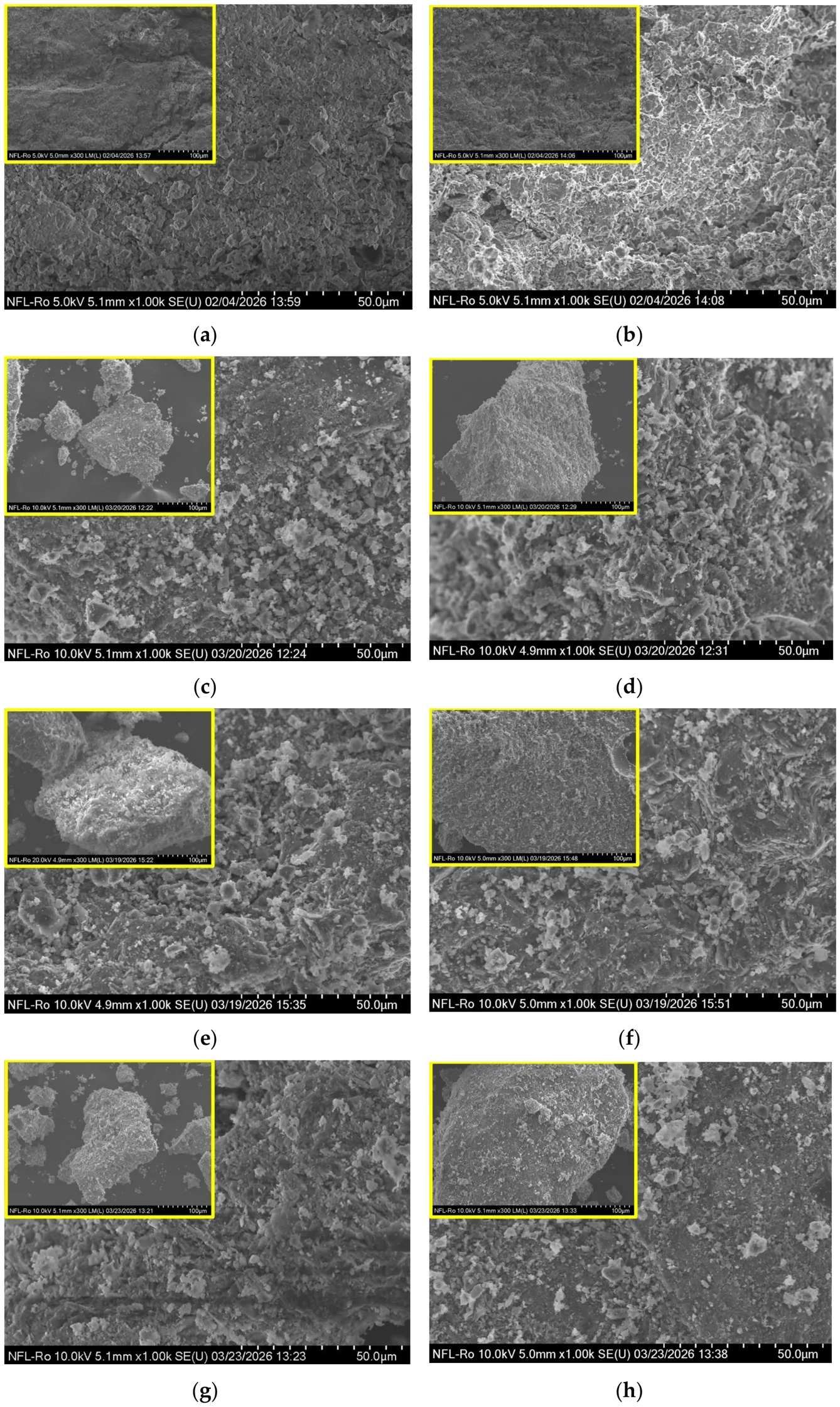

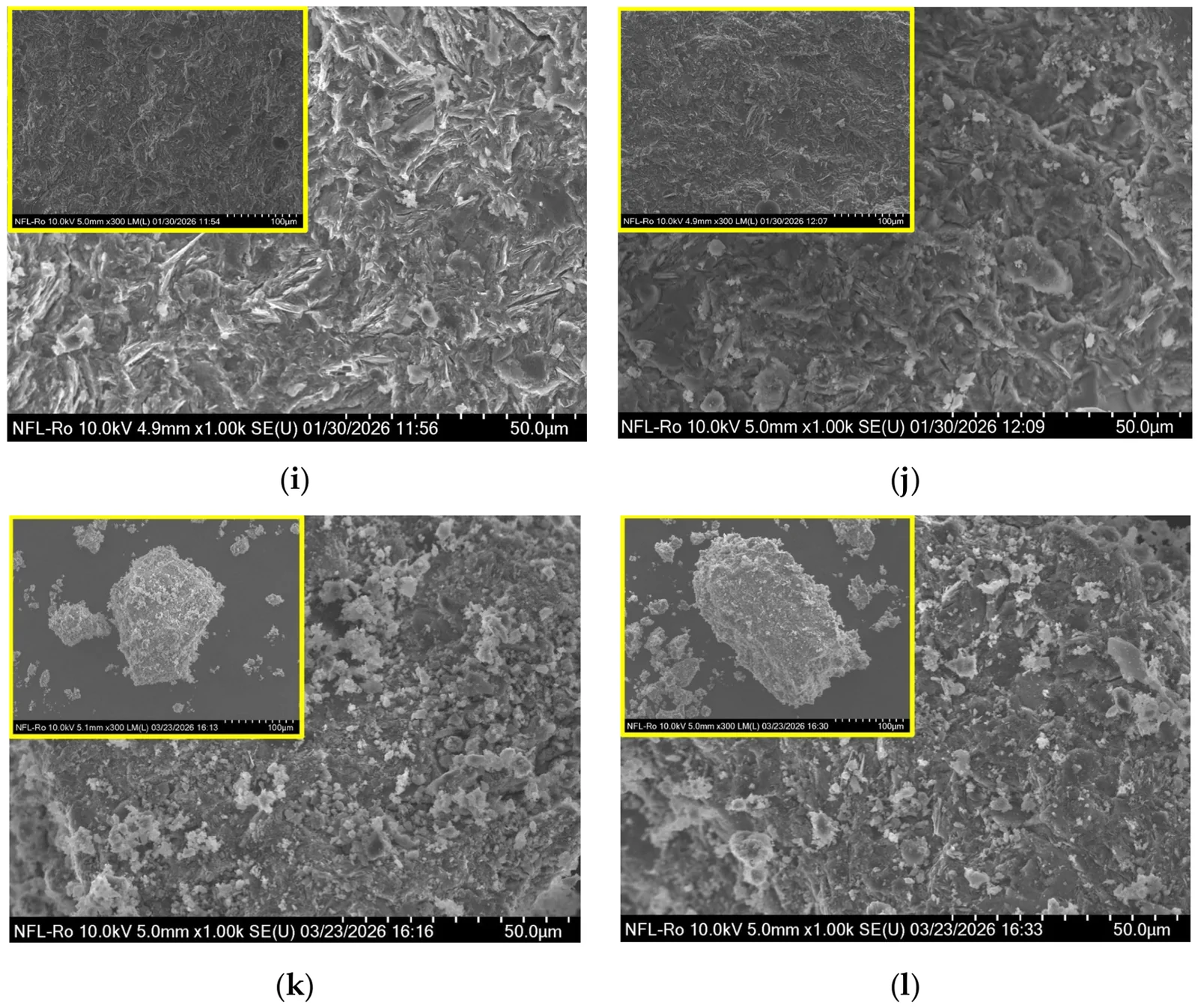
SEM micrographs of the investigated geopolymers acquired from two representative areas of each sample: (**a**,**b**) GP; (**c**,**d**) GP-CG; (**e**,**f**) GP-MA; (**g**,**h**) GP-MY; (**i**,**j**) GP-RP; and (**k**,**l**) GP-SJ. For each sample, the first and second panels correspond to areas 1 and 2, respectively. All main micrographs were acquired at ×1000 magnification, while the inserts show the corresponding overview images acquired at ×300 magnification.

In all the samples, the typical characteristics of activated alkaline geopolymeric materials are observed: irregular surfaces, aggregate structures, and the presence of compact regions associated with amorphous aluminosilicate gel as presented in Figure 5a,b. The introduction of Ca-based particles from calcined shells leads to the change in the morphology and compactness of the matrix.

The micrographs of GP-CG (Figure 5c,d) show a heterogeneous microstructure, characterized by agglomerations of relatively large size. At magnification of 300× alternating compact areas with unevenly distributed cavities and microcracks are highlighted, consistent with heterogeneous dispersion of Ca-based particles in the matrix. At 1k× a rough granular texture only partially embedded in the geopolymer mass is observed, which is consistent with heterogeneous incorporation and/or incomplete reaction of some calcium-rich particles within the aluminosilicate matrix. At the same time, the porous morphology suggests a high specific surface, favorable for photocatalytic or adsorption applications.

The SEM images from Figure 5e,f of GP-MA, present the most compact and uniform microstructure among the analyzed samples. SEM images at 300× indicate a relatively homogeneous solid phase distribution, with significant reduction in structural holes. At 1k× magnification a dense, continuous network consisting of strongly interconnected fine particles is observed, which is consistent with a compact microstructure and close physical association of CaCO_3_ particles with the aluminosilicate matrix; the extent of geopolymerization cannot, however, be independently established from SEM morphology alone.

In the case of the GP-MY, SEM micrographs from Figure 5g,h, show an intermediate structure between the observed heterogeneity for GP-CG and the compactness of the GP-MA sample. At the micrometric scale, the existence of relatively evenly distributed aggregates with moderate porosity is noted. At 1k× fine granular regions and microcavities dispersed in the matrix appear, which is consistent with heterogeneous incorporation of residual shell-derived particles within the amorphous matrix. The morphology is compatible with a moderate degree of structural integration between the calcined precursor and the geopolymeric matrix.

GP-RP has a heterogeneous microstructure, dominated by irregular particles, as shown in Figure 5i,j. At 300× an uneven distribution of solid phases is highlighted, with the existence of compact conglomerates. The images at 1k× show particles with well-defined edges, suggesting the presence of mineral fractions that are only partially incorporated into the surrounding geopolymer gel. This morphology can be advantageous for mass transfer processes and for the development of functional surface properties.

The micrographs of GP-SJ (Figure 5k,l) show a relatively compact structure, but with the presence of lamellar regions and local crystalline agglomerations. At low magnification, a more uniform surface is observed compared to GP-CG, but less dense than GP-MA. At 1k× fine formations appear distributed in matrices and areas with a compact appearance, morphologically consistent with a well-developed geopolymer gel network. The presence of lamellar structures may be associated with morphological reminiscences of calcium biominerals derived from shells or partial recrystallization of CaCO_3_-based compounds during alkaline activation.

The comparative analysis of all samples indicates that the nature of the biogenic particles used directly influences the degree of compaction, porosity distribution, and overall microstructural organization. The GP-MA and GP-SJ samples show the highest degree of structural densification processes, while the GP-CG and GP-RP show higher porosity and structural heterogeneity. The GP-MY sample exhibits intermediate behavior, characterized by a balance between compactness and porous surface development.

The presence of Ca-based particles from calcined shells leads to the formation of aluminosilicate-calcium hybrid structures with distinct microstructural properties.

### 3.5. EDX Analysis

The EDX analysis of biogenic geopolymers with Ca-based particles from calcined shells highlights the local elemental distribution and confirms the integration of calcium-based compounds into the aluminosilicate matrix. The investigation of two distinct zones for each sample provides relevant information on the chemical homogeneity, the dispersion of calcium phases and the degree of interaction between the biogenic precursor and the geopolymeric gel.

In all the samples analyzed, the EDX spectra indicate the predominance of oxygen, silicon and aluminum, elements characteristic of the geopolymer aluminosilicate network. The presence of Na and/or K confirms the participation of the alkaline activator in the polycondensation process and the formation of the (N, K)–A–S–H type gel. In addition, the identification of Ca in all modified samples demonstrates the incorporation of biogenic phases from shells and supports the XRD results on the existence of calcite or aragonite in the structure of the composites. The correlation of the Ca distribution with the local variations in Si and Al indicates the development of hybrid aluminosilicate–calcium regions, associated with the formation of secondary gels type C–(N, K)–A–S–H.

**Figure 6:**
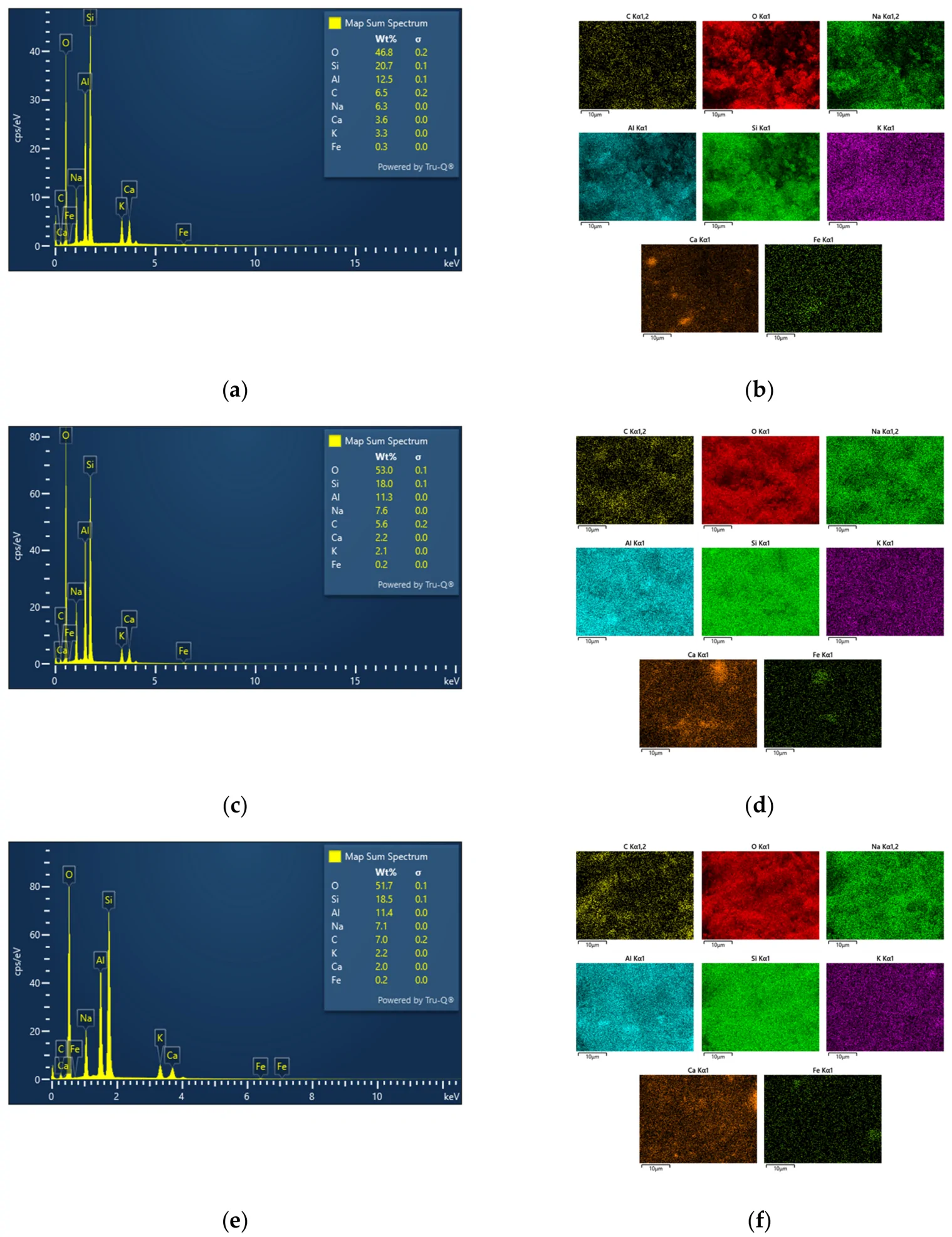

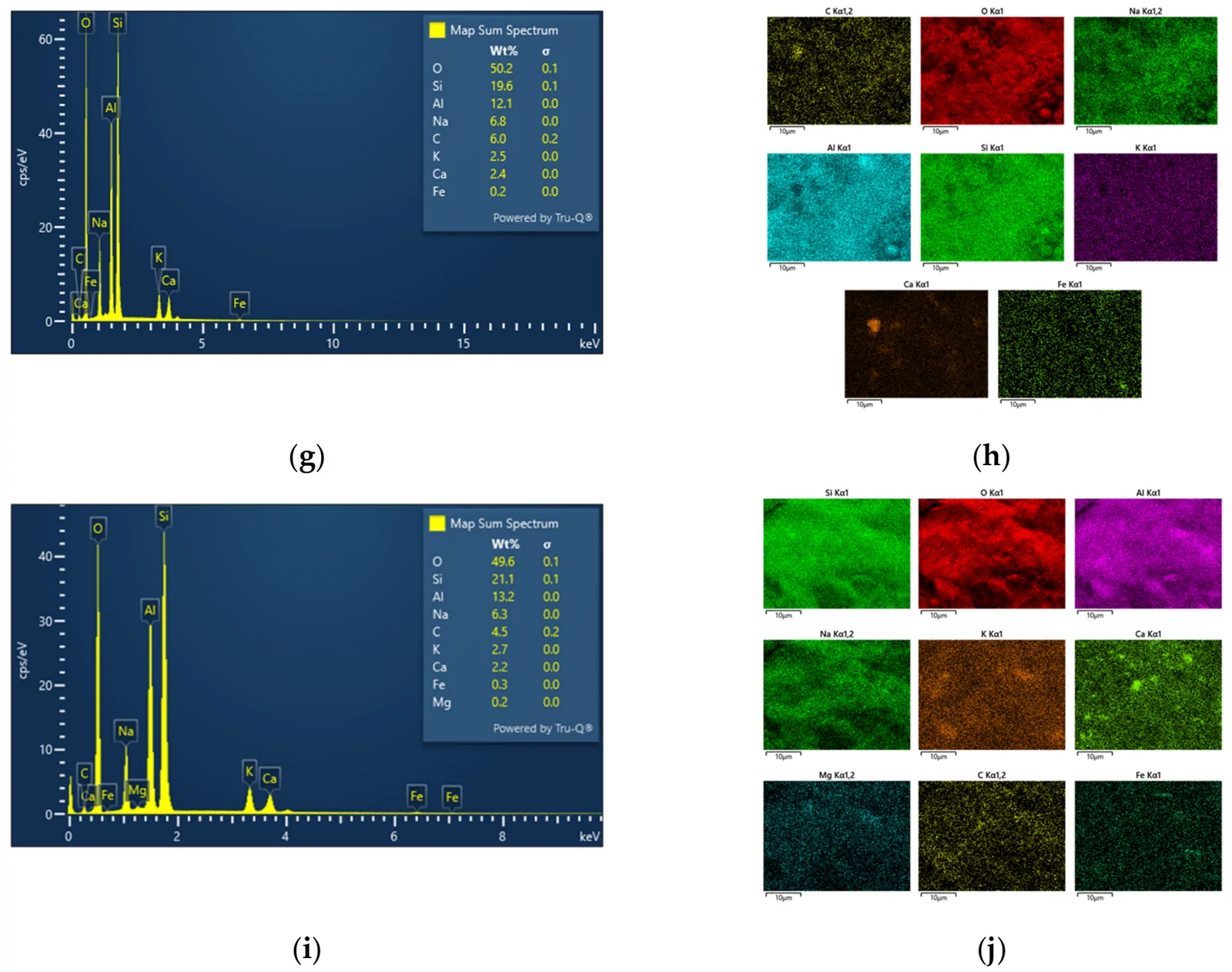
EDX spectra and corresponding elemental mapping images of the investigated shell-modified geopolymers: (**a**,**b**) GP-CG; (**c**,**d**) GP-MA; (**e**,**f**) GP-MY; (**g**,**h**) GP-SJ; and (**i**,**j**) GP-RP. For each sample, the first panel shows the EDX spectrum acquired from area 1, while the second panel presents the corresponding elemental distribution maps.

For the GP-CG sample, as seen in Figure 6a,b, and Figure S1a,b. The EDX analysis highlights significant variations between the two areas investigated, suggesting an uneven distribution of calcium particles from *Chamelea gallina*. The Ca-rich zones probably correspond to the carbonate aggregates observed by SEM, while the Si and Al-dominated zones reflect the geopolymer gel-rich regions. This chemical heterogeneity agrees with the morphology observed by SEM and the high BET values, indicating a developed but partially ununiform mesoporous network.

Figure 6c,d and Figure S1c,d, the micrographs of GP-MA, show the differences between zone 1 and zone 2 are smaller, suggesting a more homogeneous distribution of calcium phases in the aluminosilicate matrix. This uniformity is compatible with the compact microstructure observed by SEM and indicates a high degree of integration of Ca-based compounds into the geopolymer network. The correlation with BET shows that this structural densification is accompanied by a reduction in specific surface area and pore volume, suggesting that calcitic particles contribute to compaction and partial blockage of mesopores.

For GP-MY, EDX confirms the presence of calcium associated with the aragonitic phase identified by XRD, as shown in Figure 6e,f and Figure S1e,f. The relatively variable distribution of Ca between the two zones suggests that particles from *Mytilus edulis* are moderately dispersed in the matrix and partially retain their original biogenic character. Correlation with SEM indicates the existence of granular regions and unevenly distributed microcavities, and intermediate BET values confirm the development of a moderate mesoporous structure. This combination suggests a balance between compactness and surface accessibility.

The GP-SJ sample shows the most favorable chemical and textural distribution. The EDX indicates an important and relatively uniform presence of calcium in both analyzed areas, simultaneously associated with Si and Al, which demonstrates an efficient integration of the calcium phase into the geopolymer matrix, as presented in Figure 6g,h and Figure S1g,h. This local homogeneity supports the BET and BJH results, where GP-SJ exhibits the largest specific surface area and largest pore volume. Correlation with SEM shows that the uniform distribution of Ca-based compounds favors the development of a well-connected mesoporous network and an extensive active surface.

In the case of GP-RP, the EDX analysis highlights a more heterogeneous distribution of Ca between the two areas, suggesting the existence of local regions rich in particles from *Rapana venosa*, as seen in Figure 6i,j and Figure S1i,j. This behavior is compatible with SEM morphology, where irregular conglomerates and porous regions separated by compact areas are observed. Correlation with BET indicates moderate textural development, suggesting that calcium particle integration is less efficient compared to GP-SJ and GP-CG.

EDX analysis complements SEM, XRD, and BET by confirming the local chemistry of the analyzed areas. Since EDX is a point or local method, differences between zone 1 and zone 2 should be interpreted as variations in chemical homogeneity, not as changes in the overall composition of the entire sample, as presented in Table 4.

The EDX analysis indicates a specific composition of calcium-phase modified aluminosilicate geopolymers: Si, Al, O, Na/K and Ca, presented in Table 5. The calculated ratios (directly from EDX-determined weight percentages reported in Table 4) show that all samples have Si/Al values ≈ 1.60–1.64, which is consistent with the formation of a stable geopolymer network, type N–A–S–H, with a relatively well polycondensed three-dimensional structure.

The calculation of the Si/Al, Na/Al, (Na+K)/Al, Ca/Si, Ca/Al and Ca(Si+Al) ratios according to the table indicates that all samples show a well-formed geopolymer matrix, as the Si/Al ratio remains in a narrow range, close to 1.6. This constancy suggests that the addition of calcined shell powders does not destabilize the main aluminosilicate network but acts primarily as a mineral and textural modifier. The Na/Al ratio, with mean values between 0.47 and 0.58, indicates a moderate alkaline compensation of the tetrahedra [AlO_4_]^−^. The subunit values suggest that there is no major excess of free sodium, which is favorable for chemical stability and for reducing the risk of efflorescence. The GP-RP sample has the lowest Na/Al value, which may indicate a less alkaline matrix or a lower participation of residual Na in the analyzed area.

Calcium ratios differentiate the samples most clearly. GP-MA has the highest mean values Ca/Si = 0.16, Ca/Al = 0.27 and Ca/(Si+Al) = 0.10, indicating the strongest influence of calcium phases on the matrix. This is consistent with the SEM–BET interpretation: the MA sample appears more compact and has the smallest BET surface area among the modified samples, suggesting that calcium particles may contribute to densification and partial pore blockage. GP-SJ and GP-CG have moderate but balanced calcium values. This balance appears important: the calcium content is sufficient to modify the structure and generate active interfaces, but not so high as to produce excessive compaction. This indicates the high BET surface area and the development of mesoporosity for these samples. GP-SJ remains the most favorable test by correlating balanced EDX composition, maximum BET and high pore volume.

GP-RP has Ca/Si and Ca/Al values close to GP-CG, but with lower Na/Al. This sample can be interpreted as a system with relatively important local calcium integration, but with a less pronounced alkaline activation in the areas analyzed. For this reason, the effect on porosity is moderate, and the structure can be more compact than in the case of GP-SJ and GP-CG.

GP-MA > GP-RP ≈ GP-CG > GP-SJ > GP-MY

However, for functional properties, the best combination is not given by the maximum amount of Ca, but by the balance between Ca, Si/Al and porosity. Thus, GP-SJ and GP-CG remain favorable for antibacterial and adsorbent applications, while GP-MA is more relevant for compaction and structural stability.

### 3.6. Preliminary Antibacterial Study

The preliminary antibacterial study of the developed geopolymer composites was evaluated using the Gram-negative reference bacterial strain, *Escherichia coli*, as presented in Table 6. The positive control exhibited a slight decrease in viable bacteria during the experimental period, from 94 CFU mL^−1^ after 30 min to 76 CFU mL^−1^ after 24 h, indicating that the bacterial suspension remained viable throughout the experiment and that no significant natural inactivation occurred. The negative control remained sterile during the entire testing period, confirming the absence of external contamination.

**Table 6:** Antibacterial activity against *E. coli*.

Samples	CFU mL^−1^		
	After 30 min	After 4 h	After 24 h
Control −	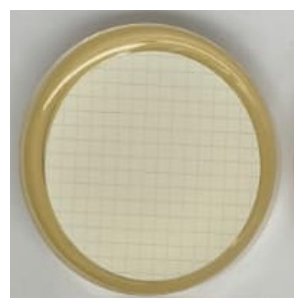	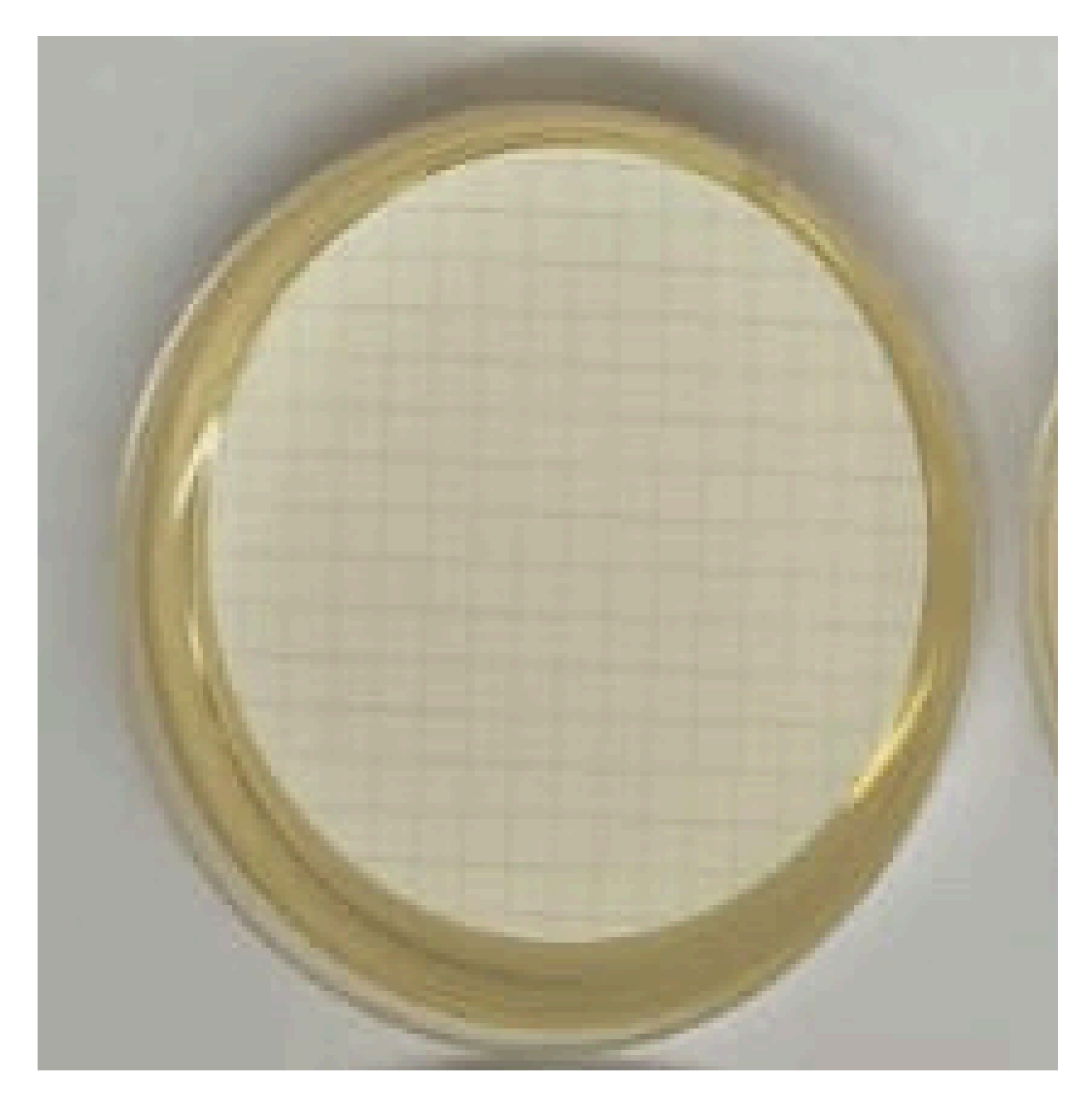	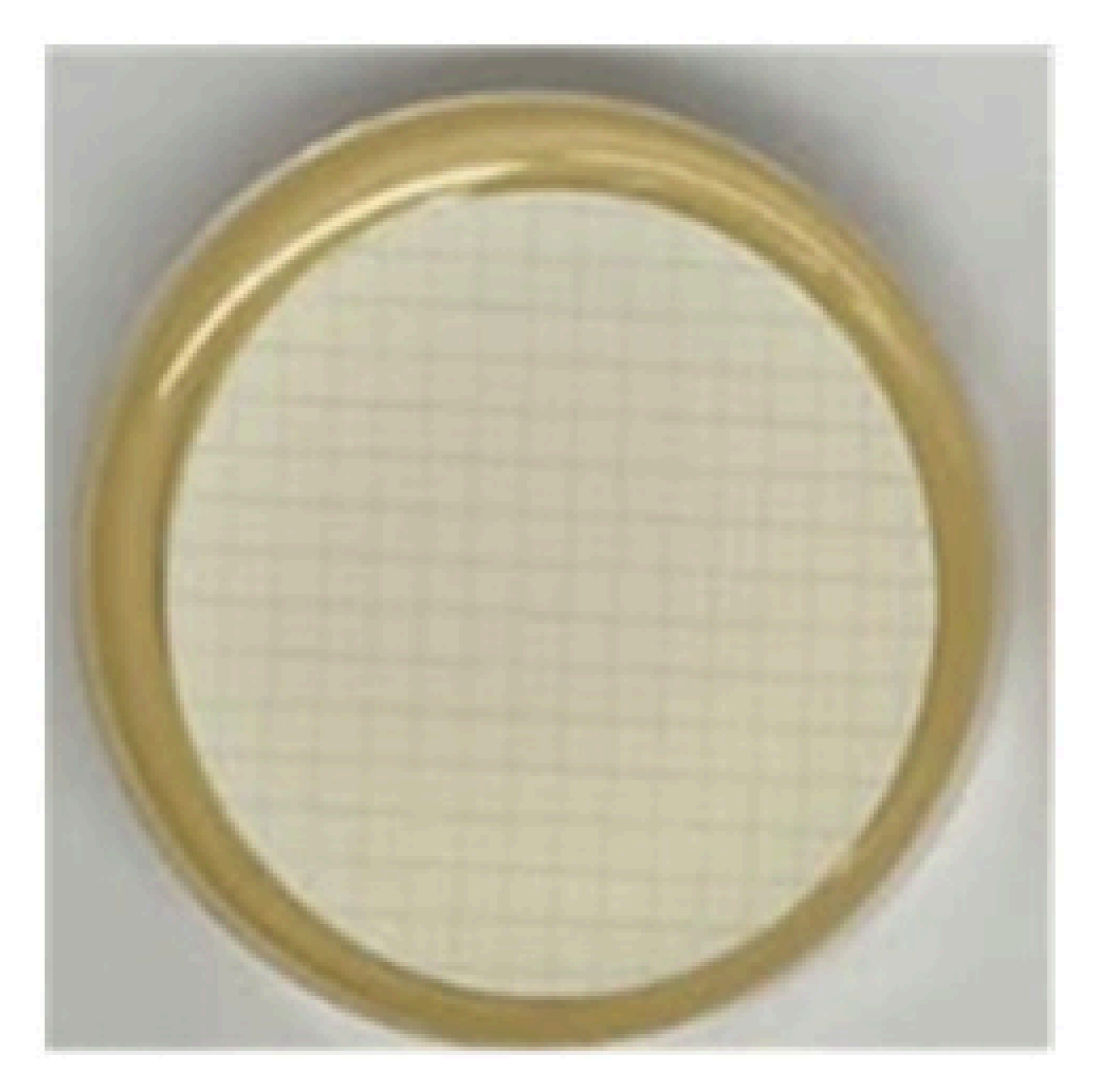
Control +	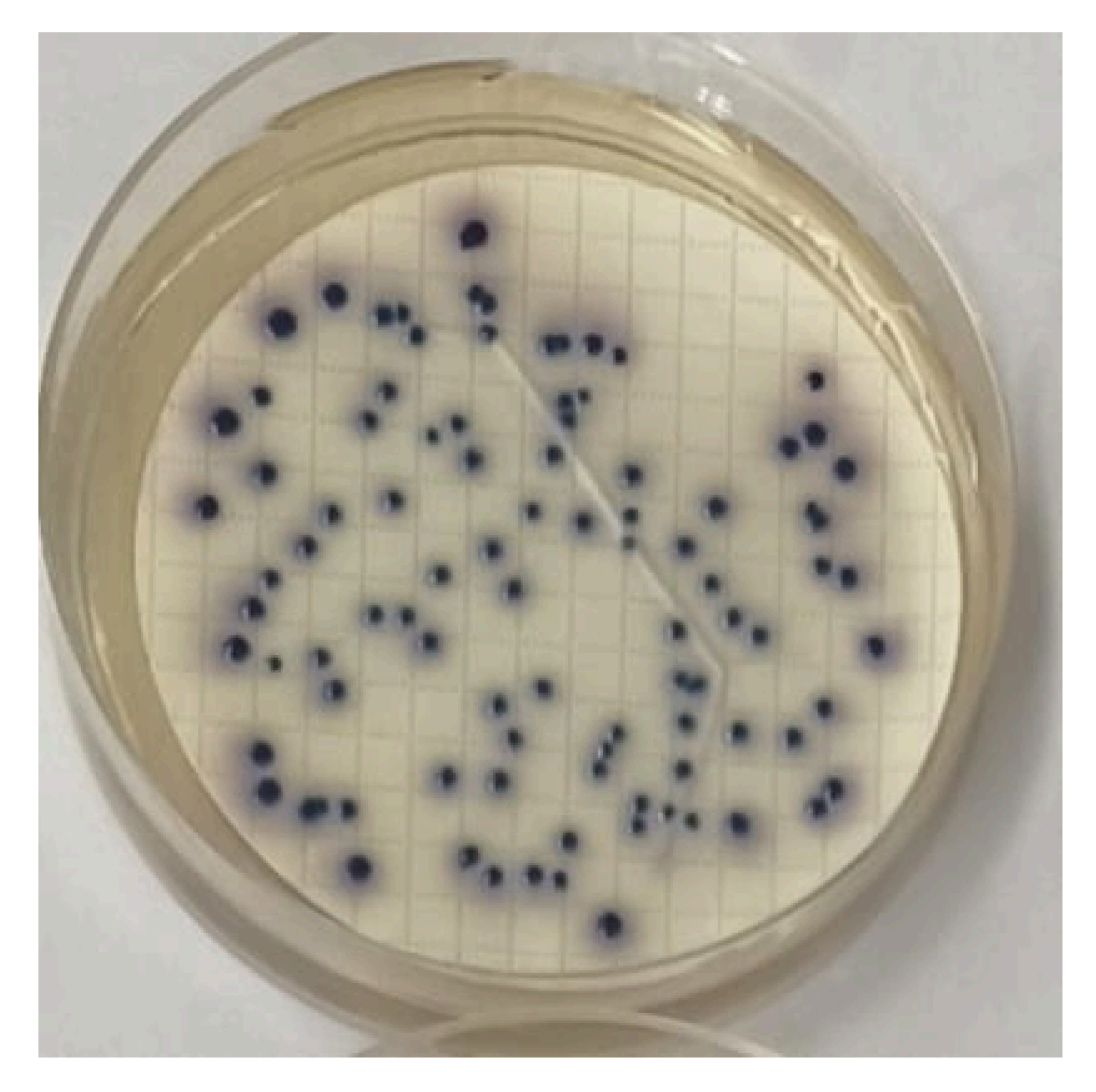	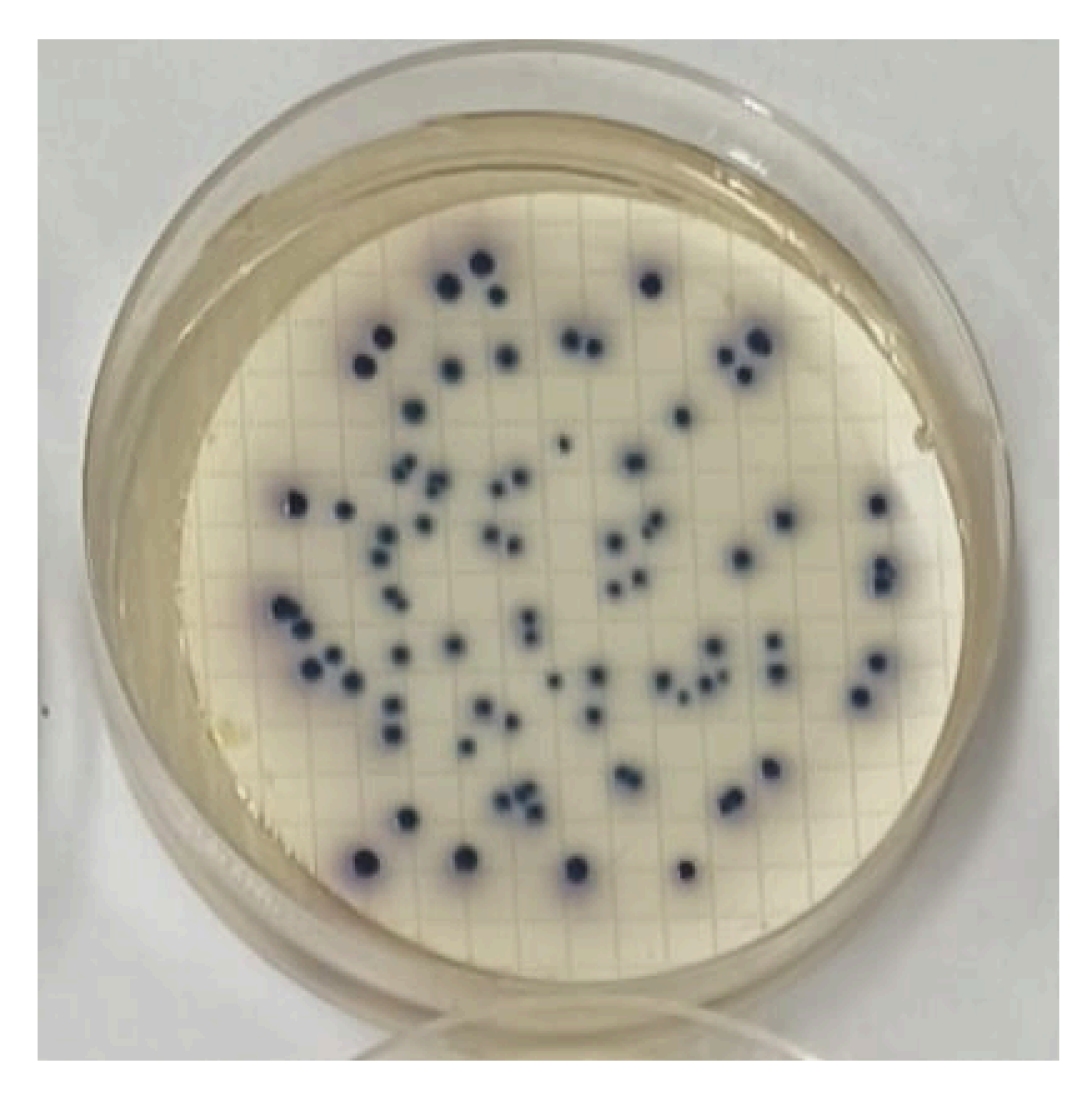	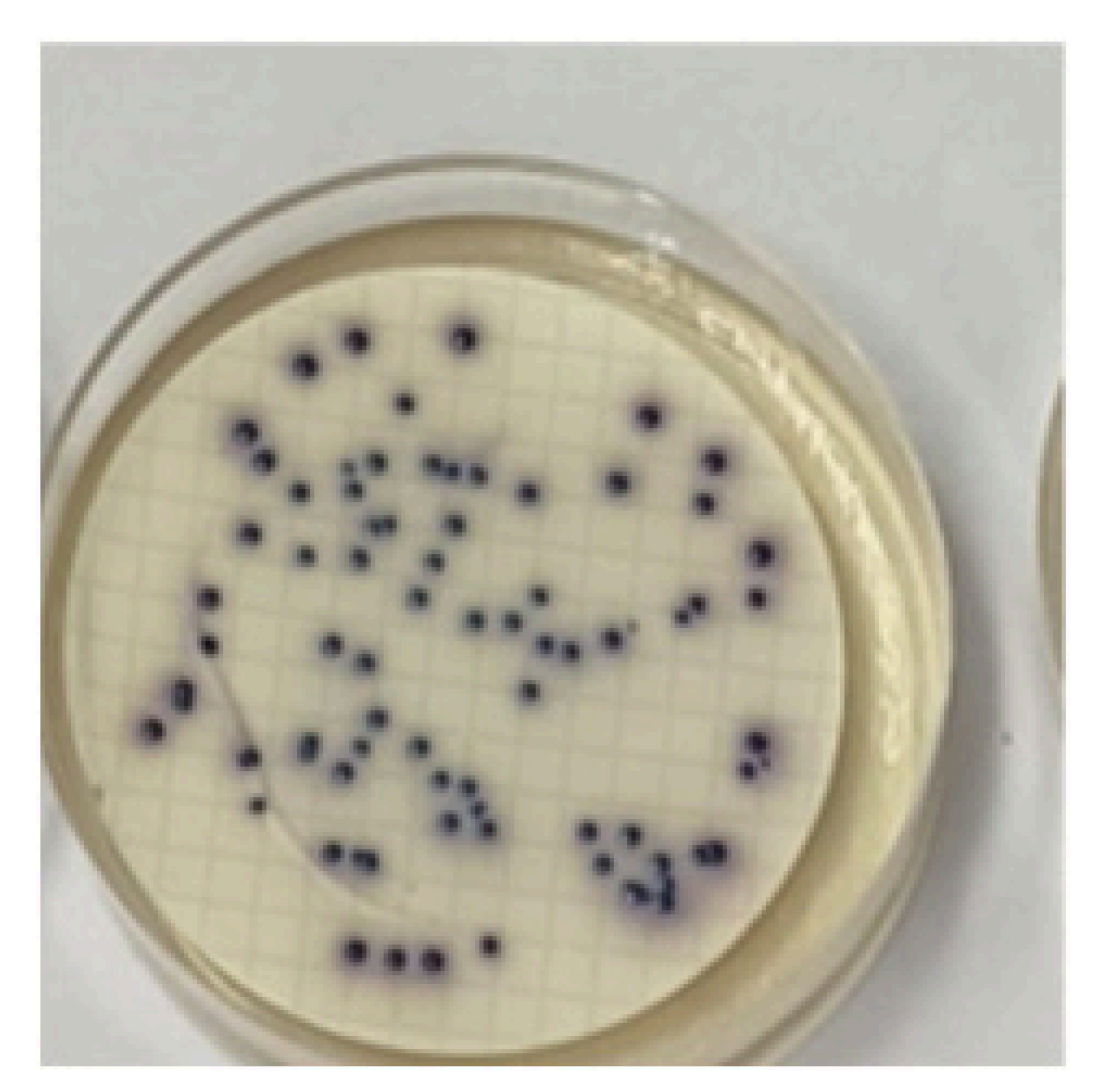
GP	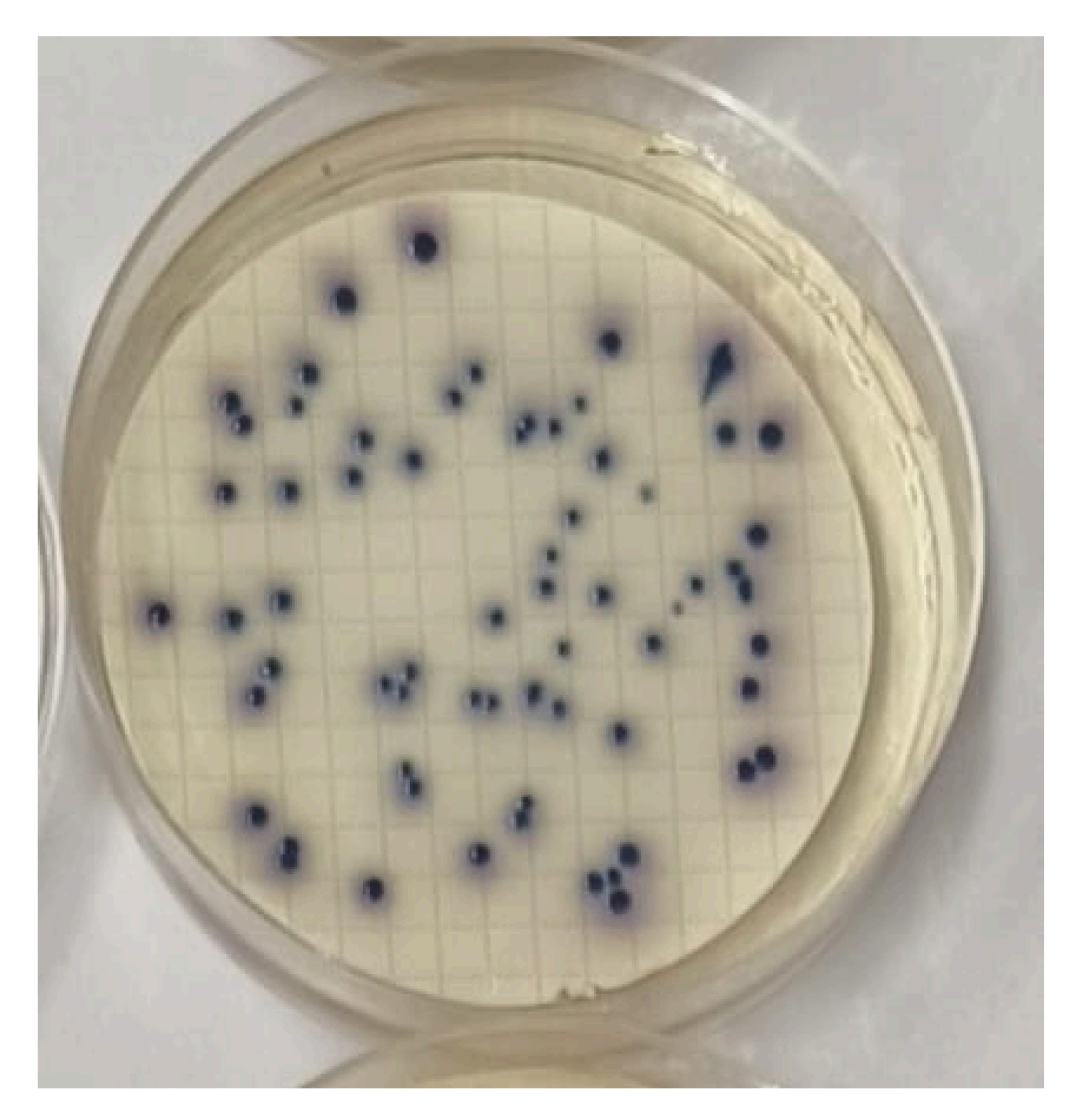	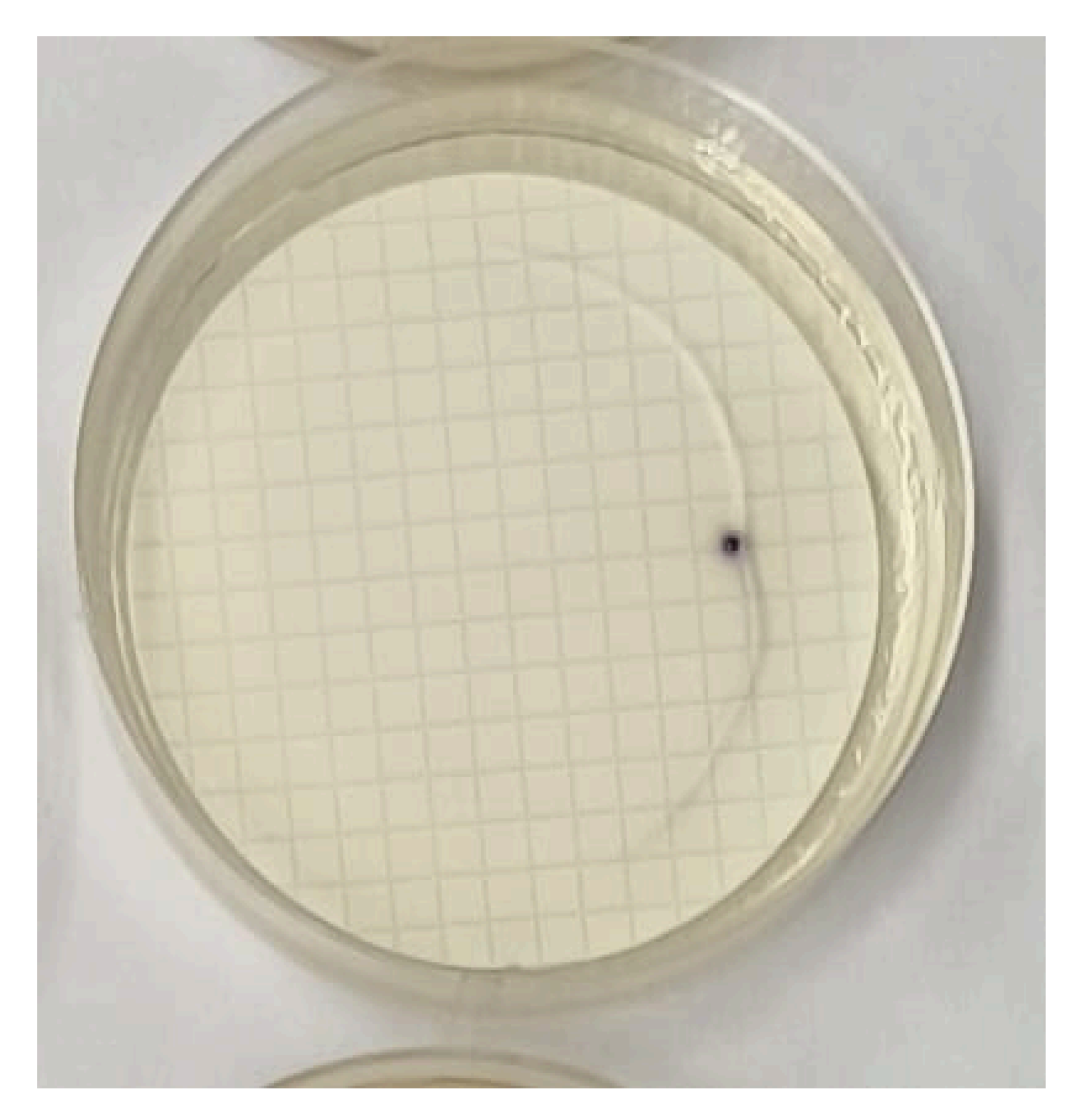	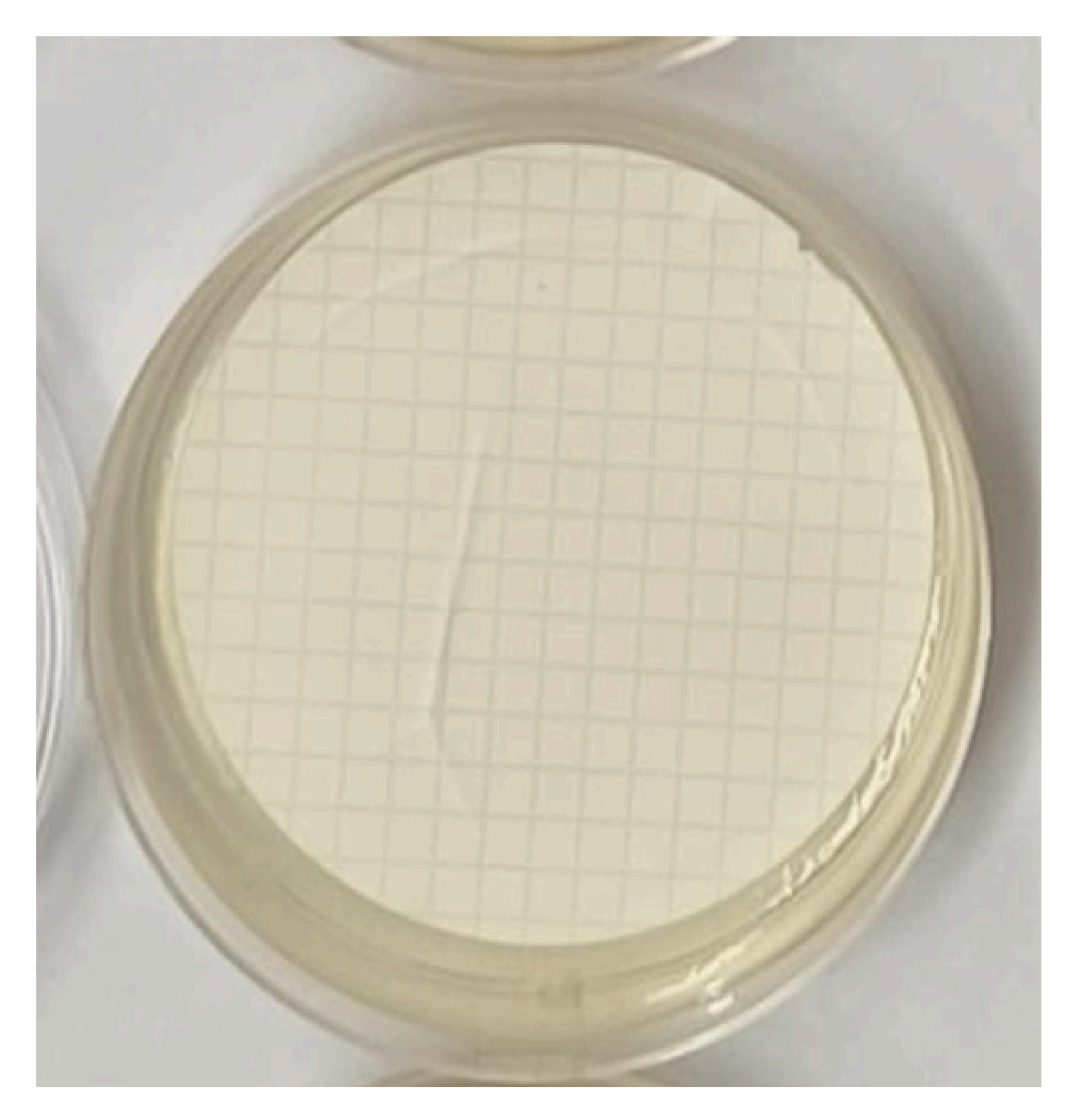
GP-MA	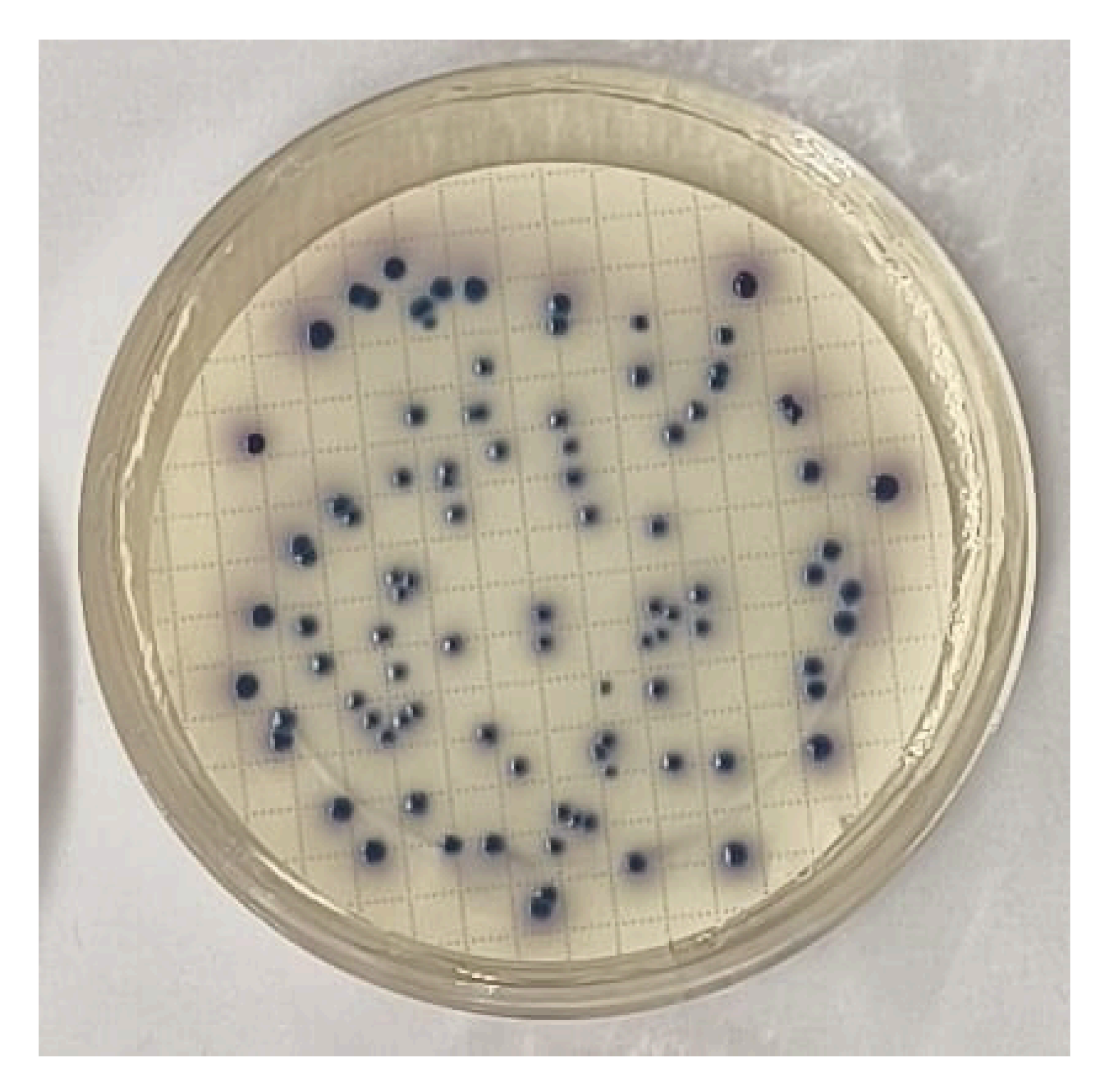	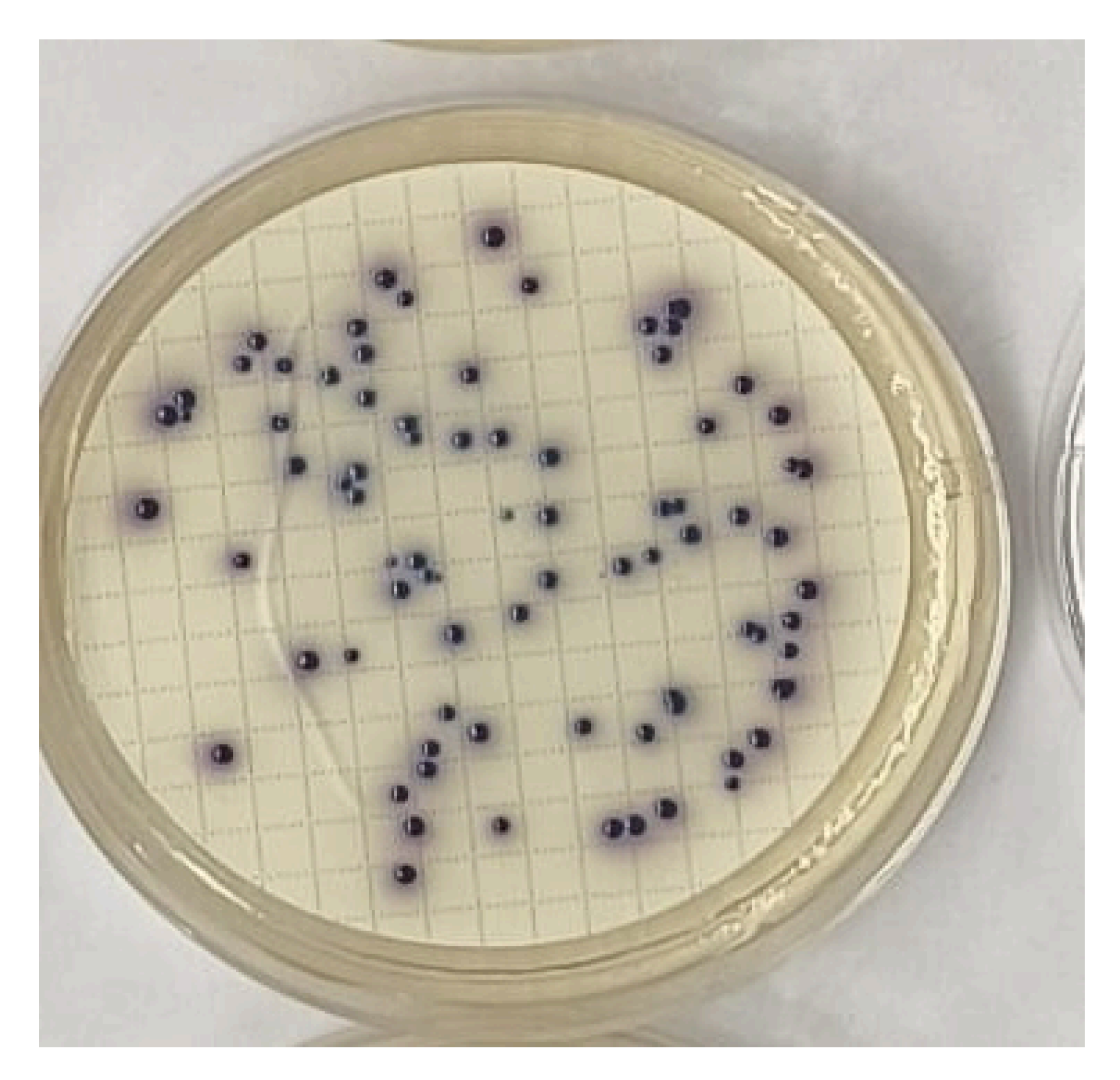	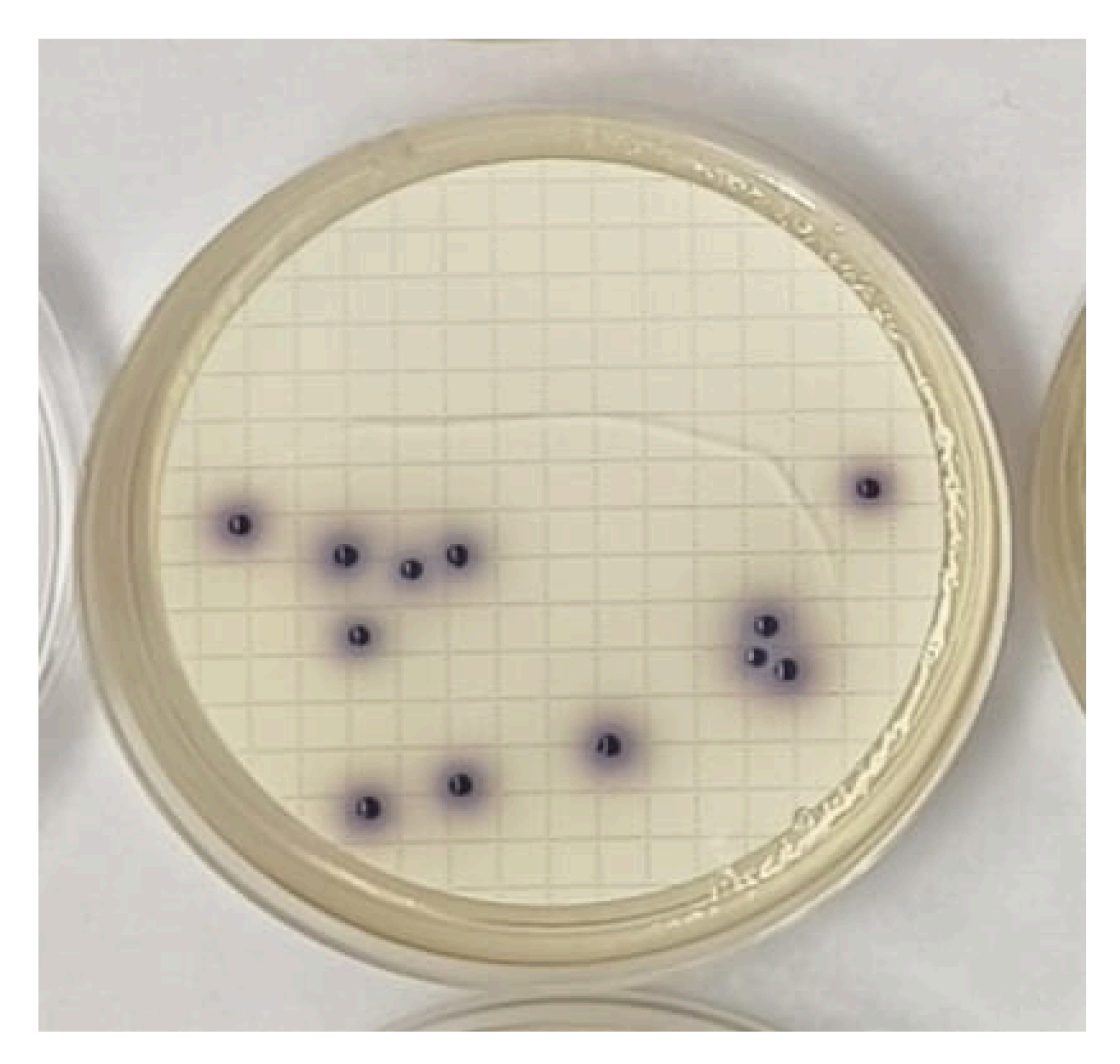
GP-CG	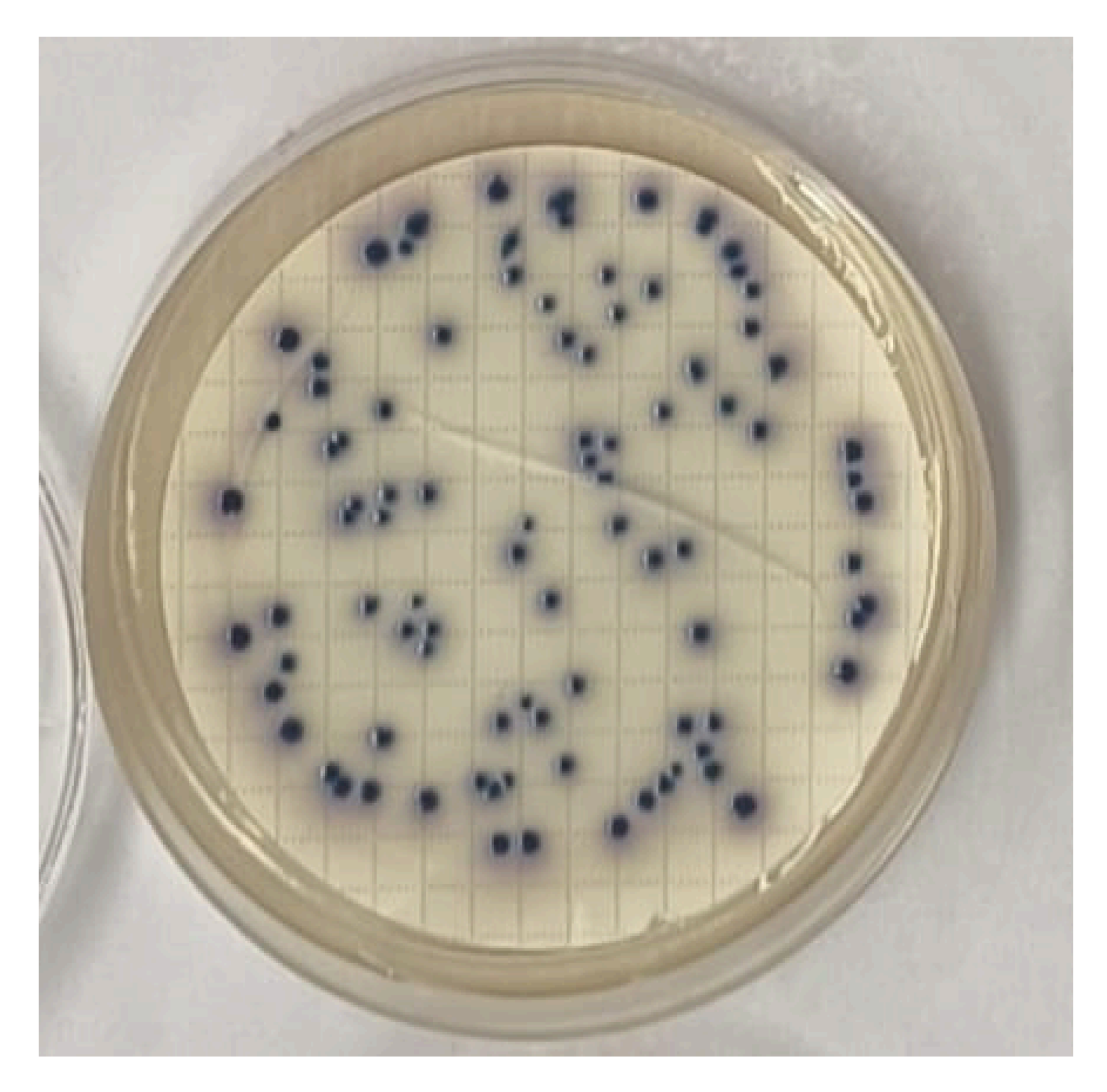	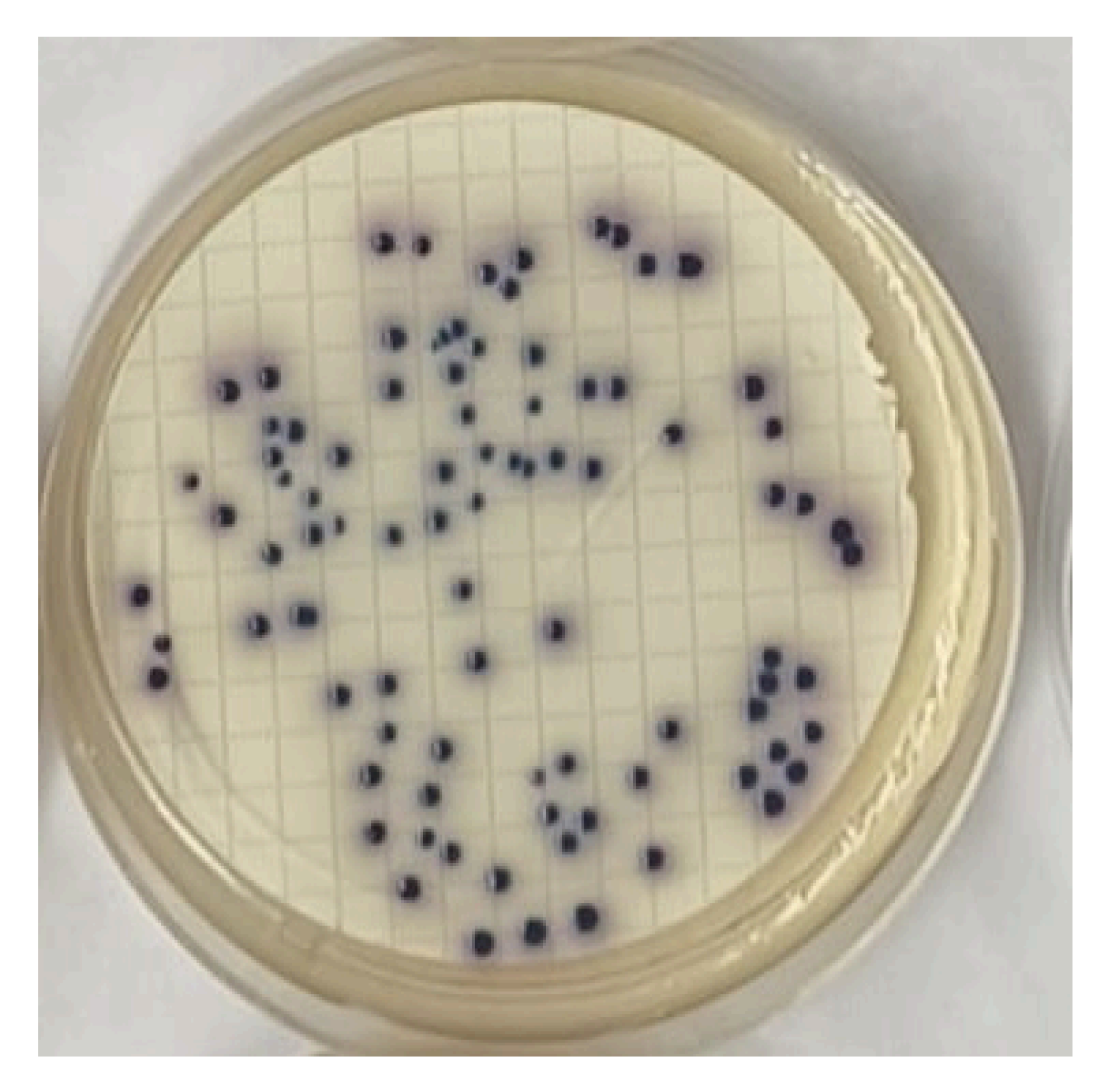	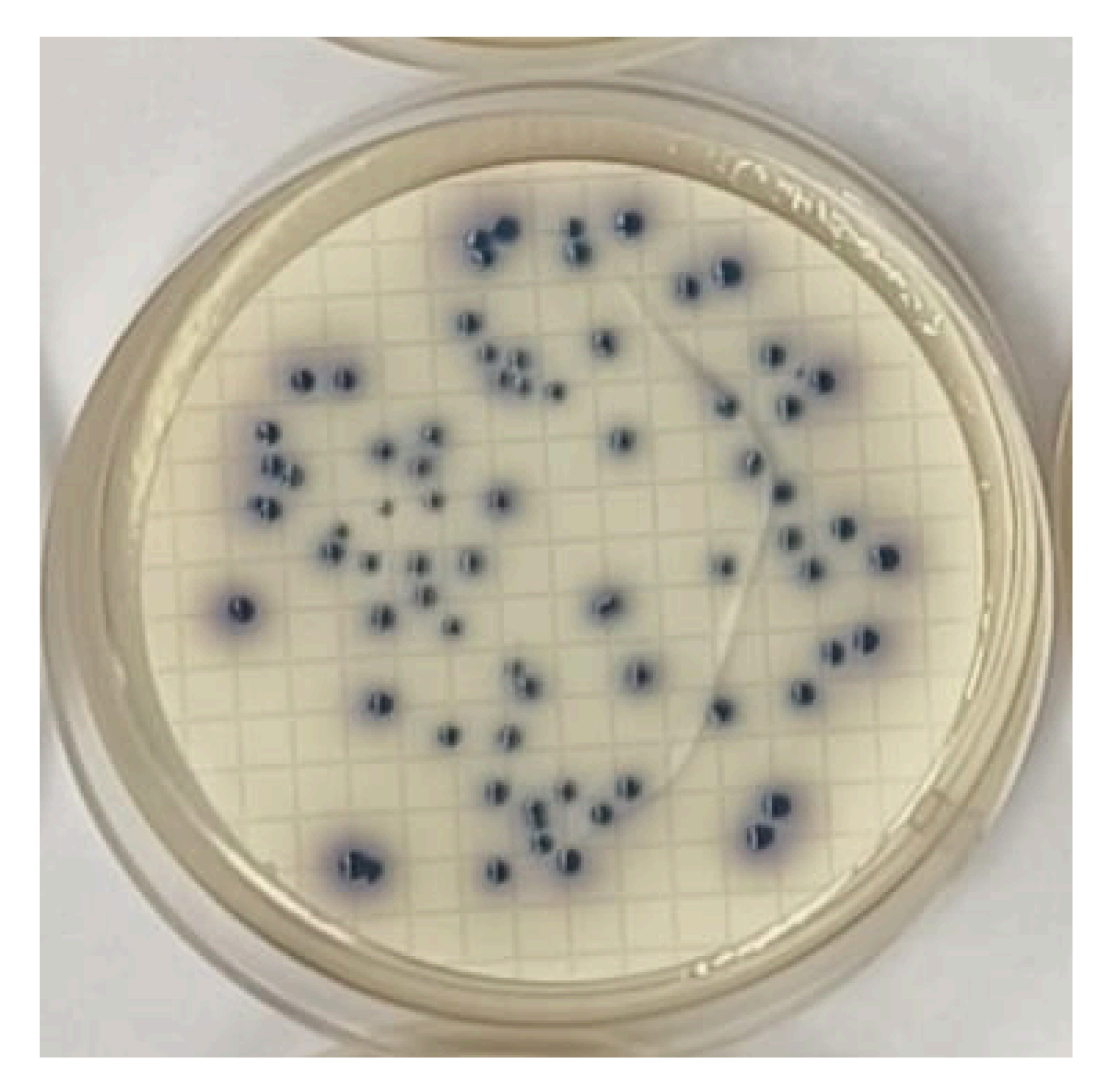
GP-MY	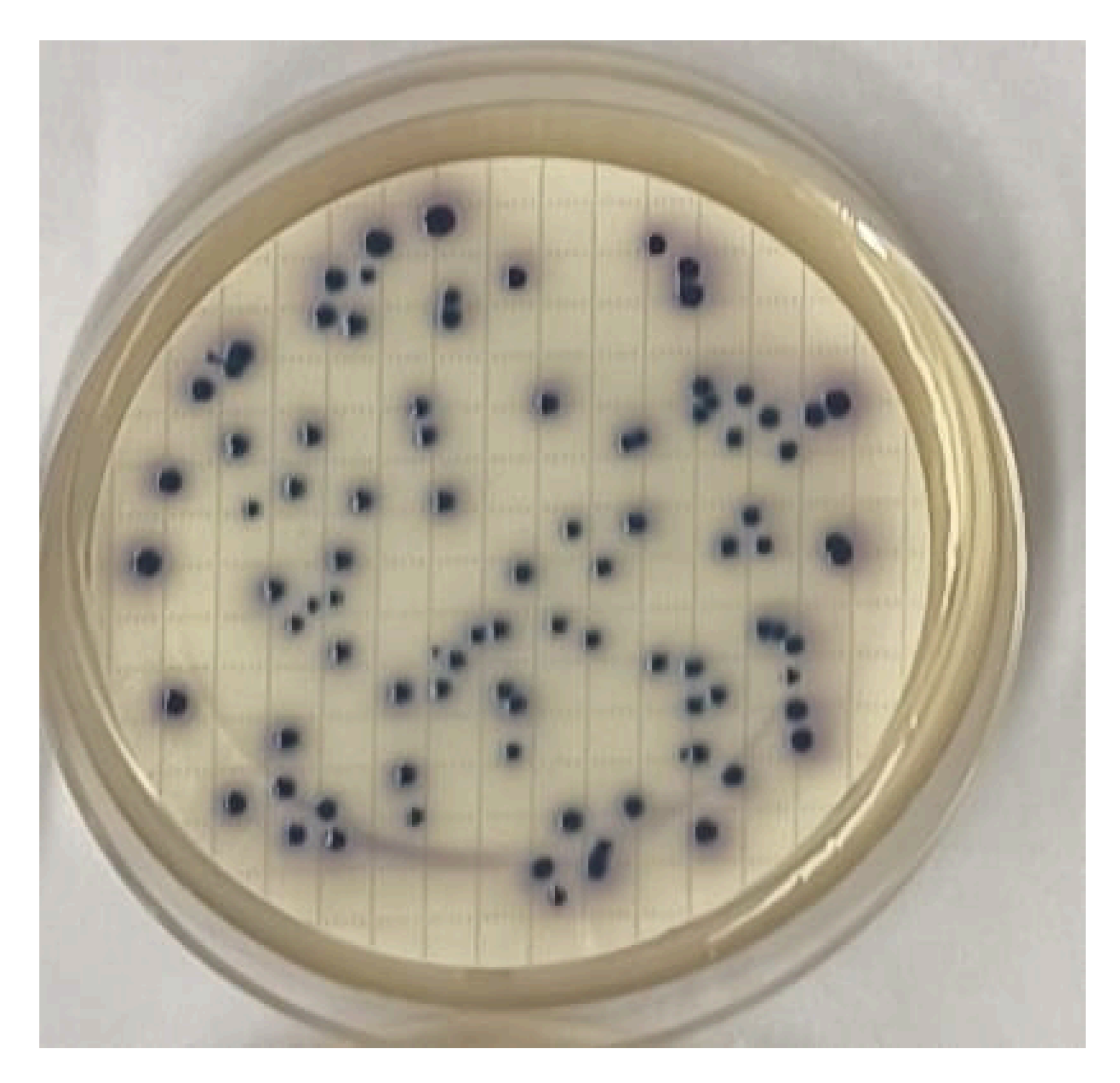	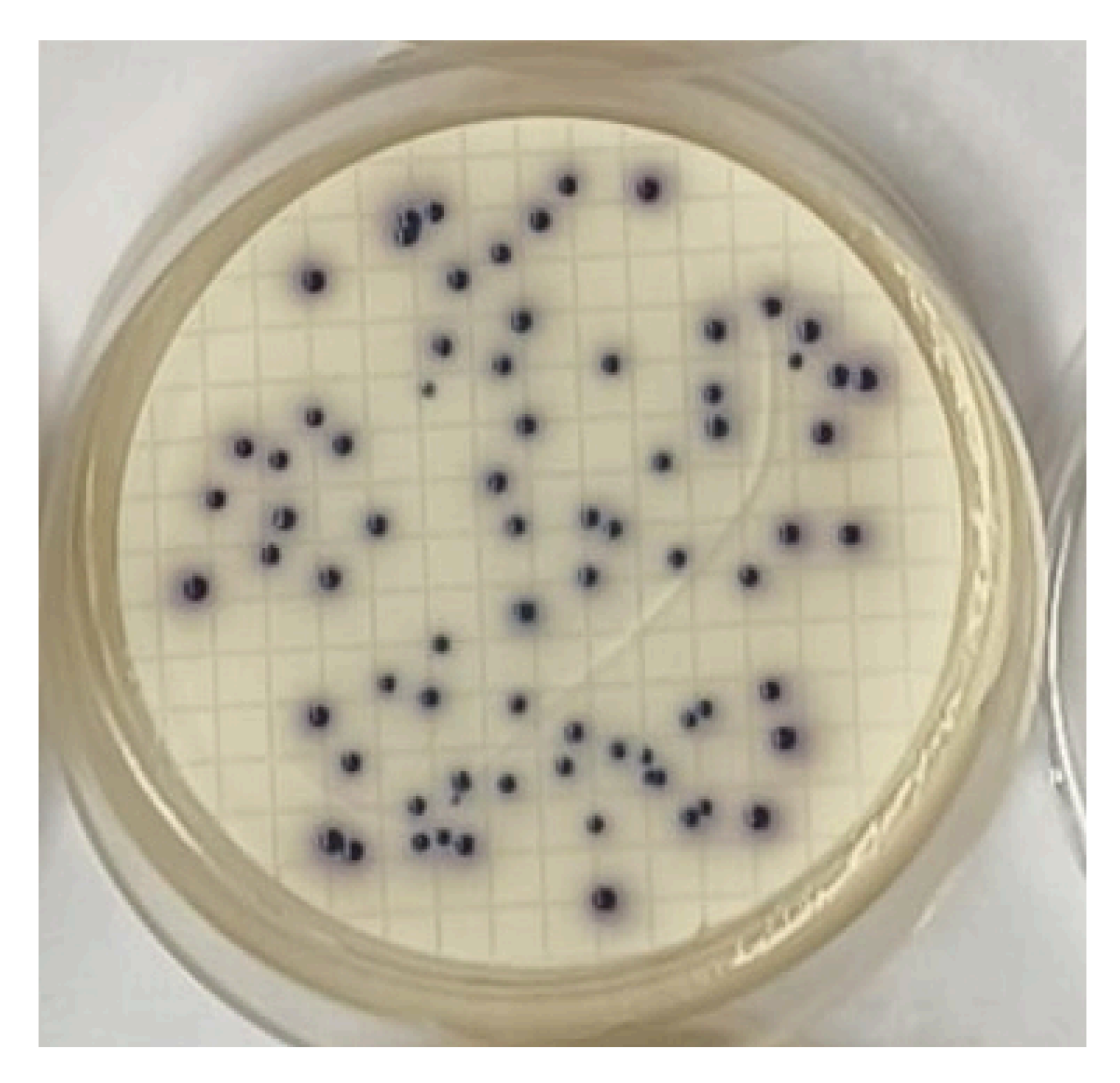	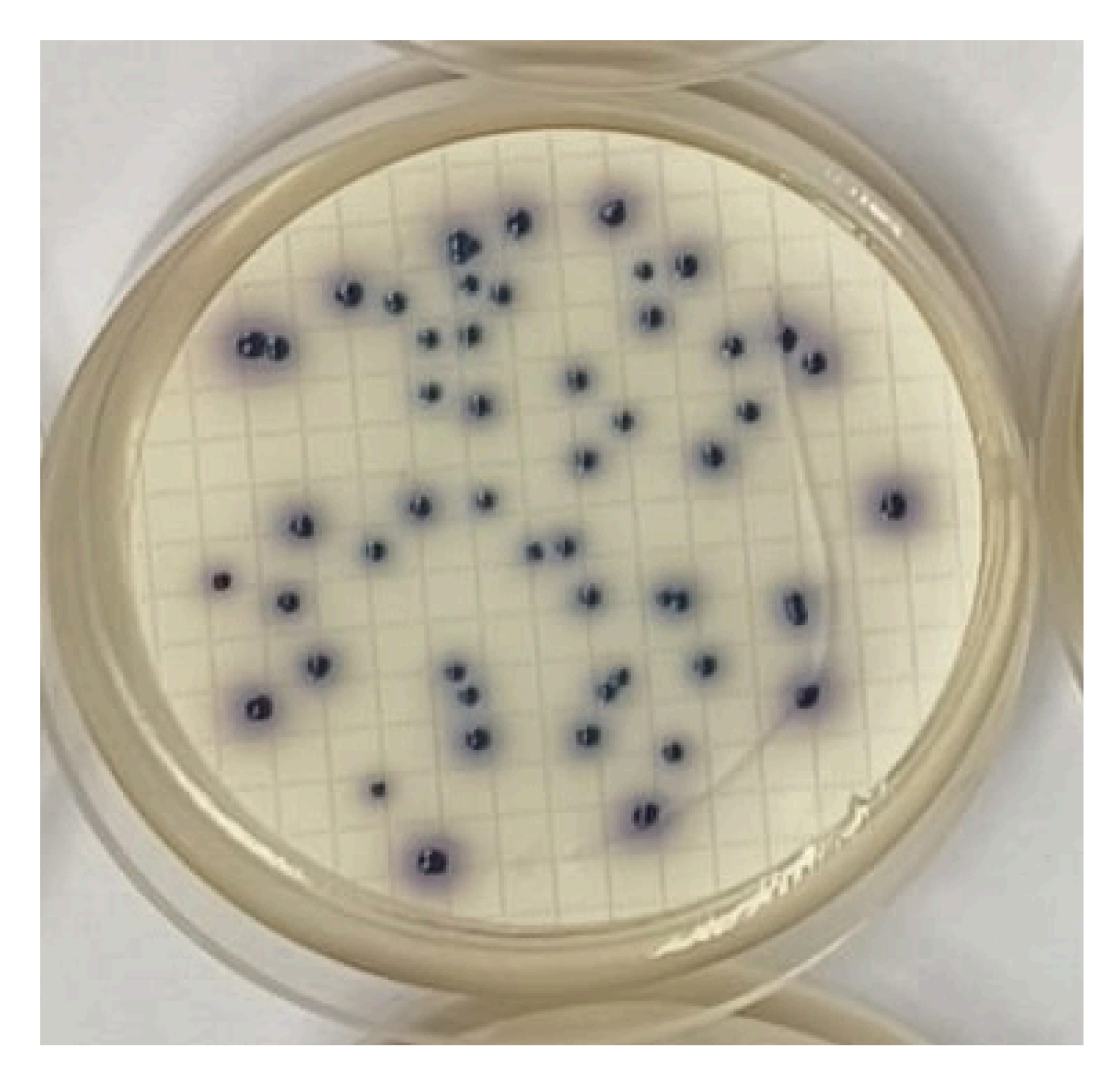
GP-SJ	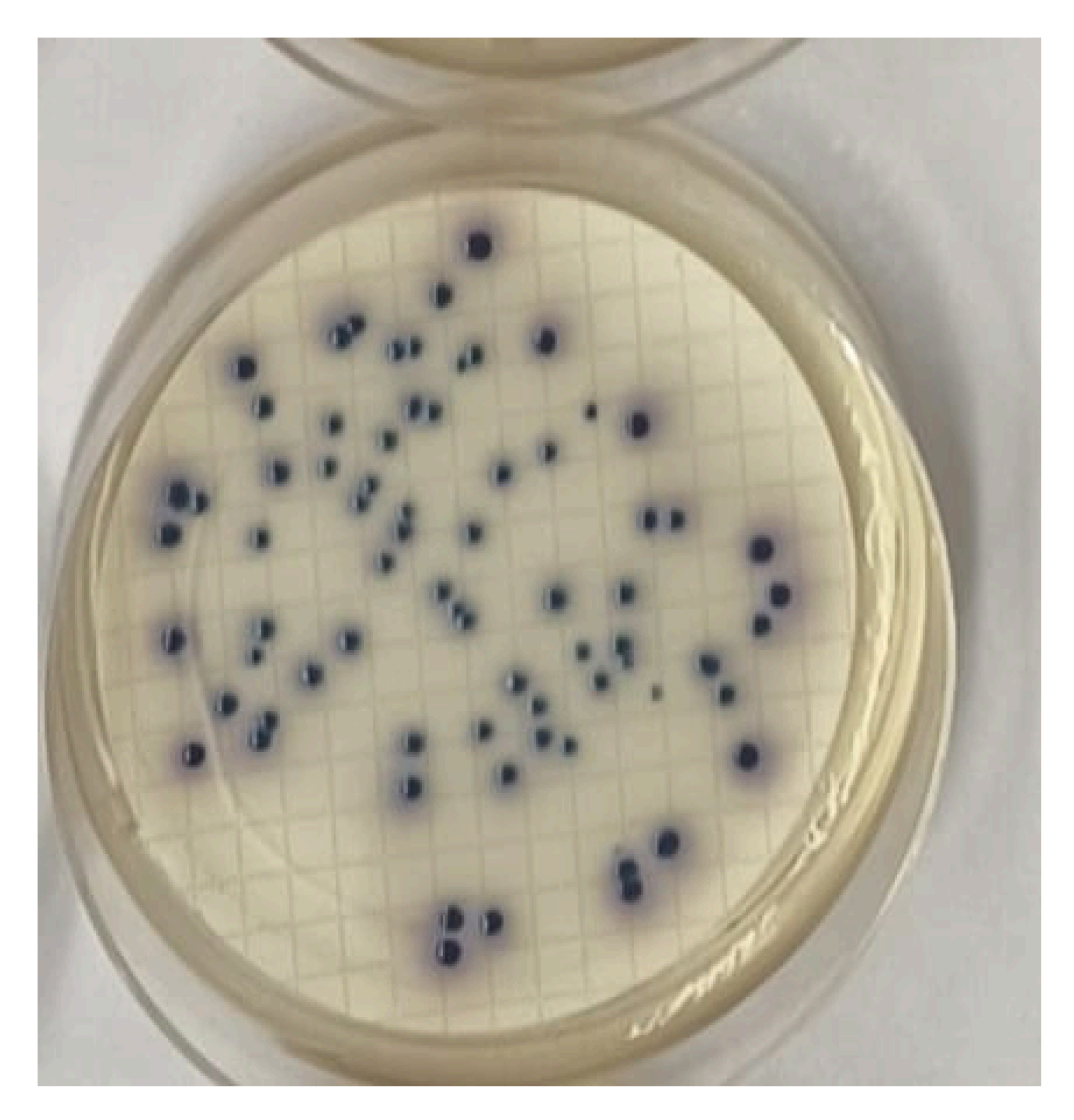	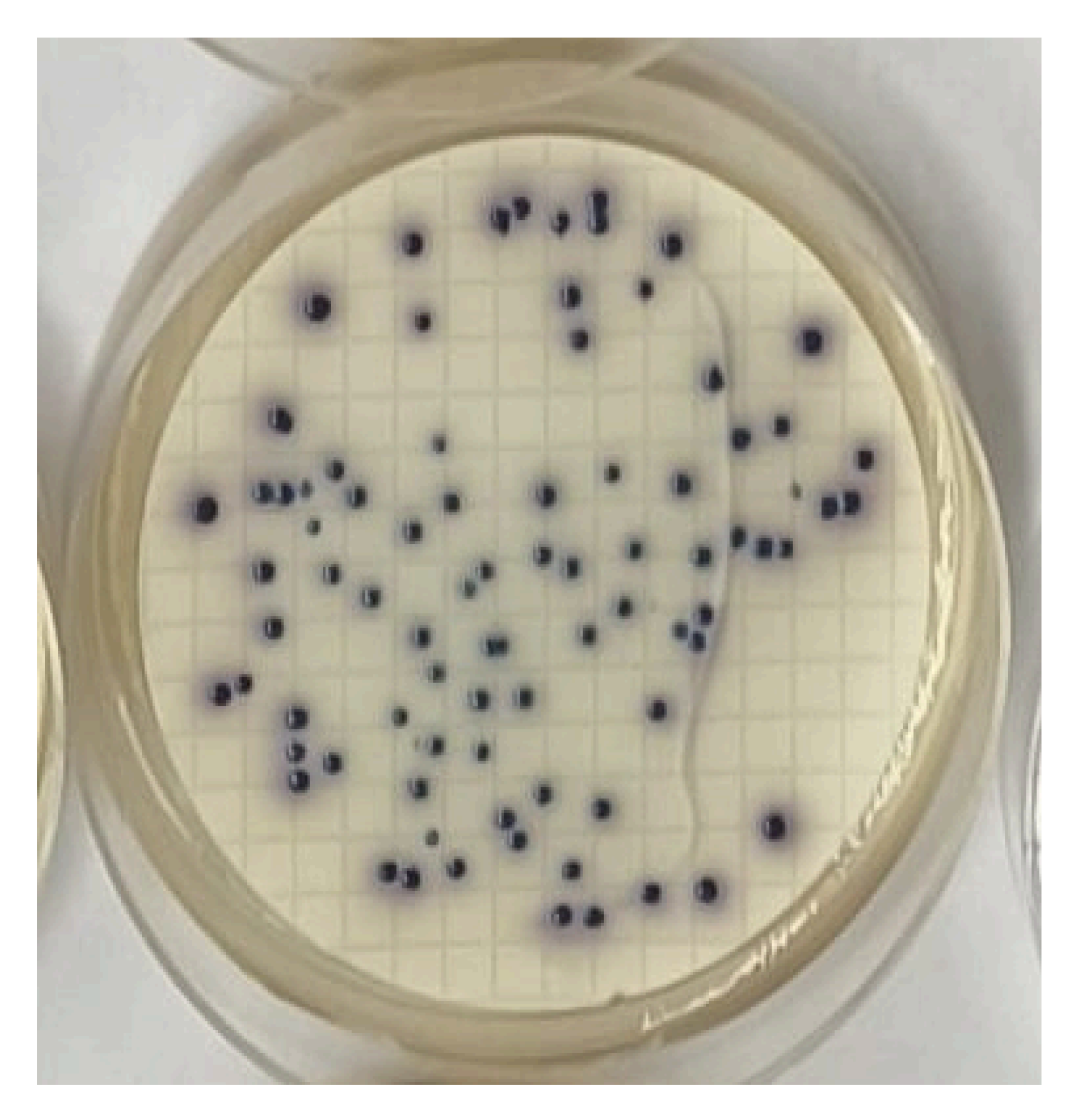	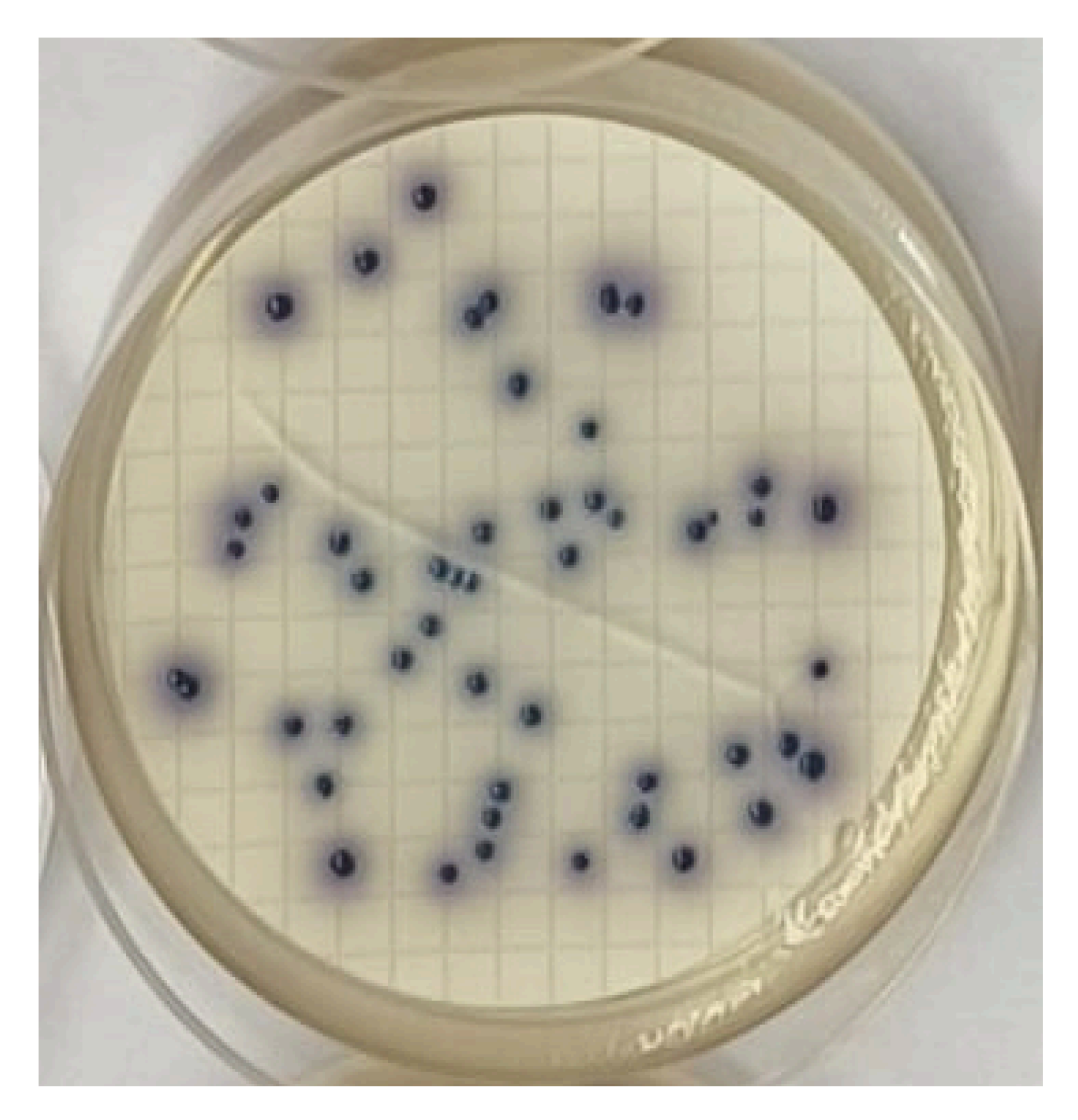
GP-RP	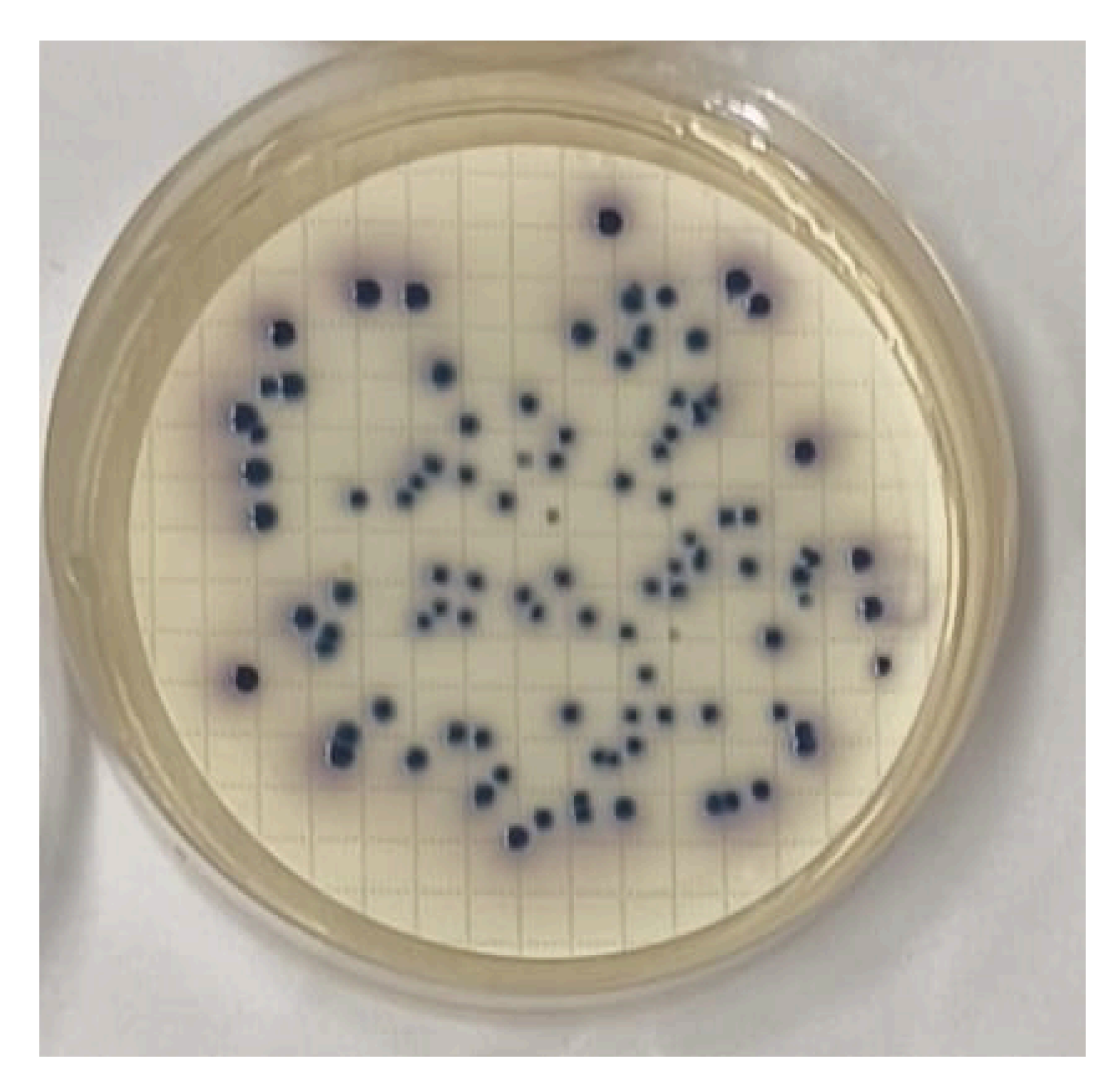	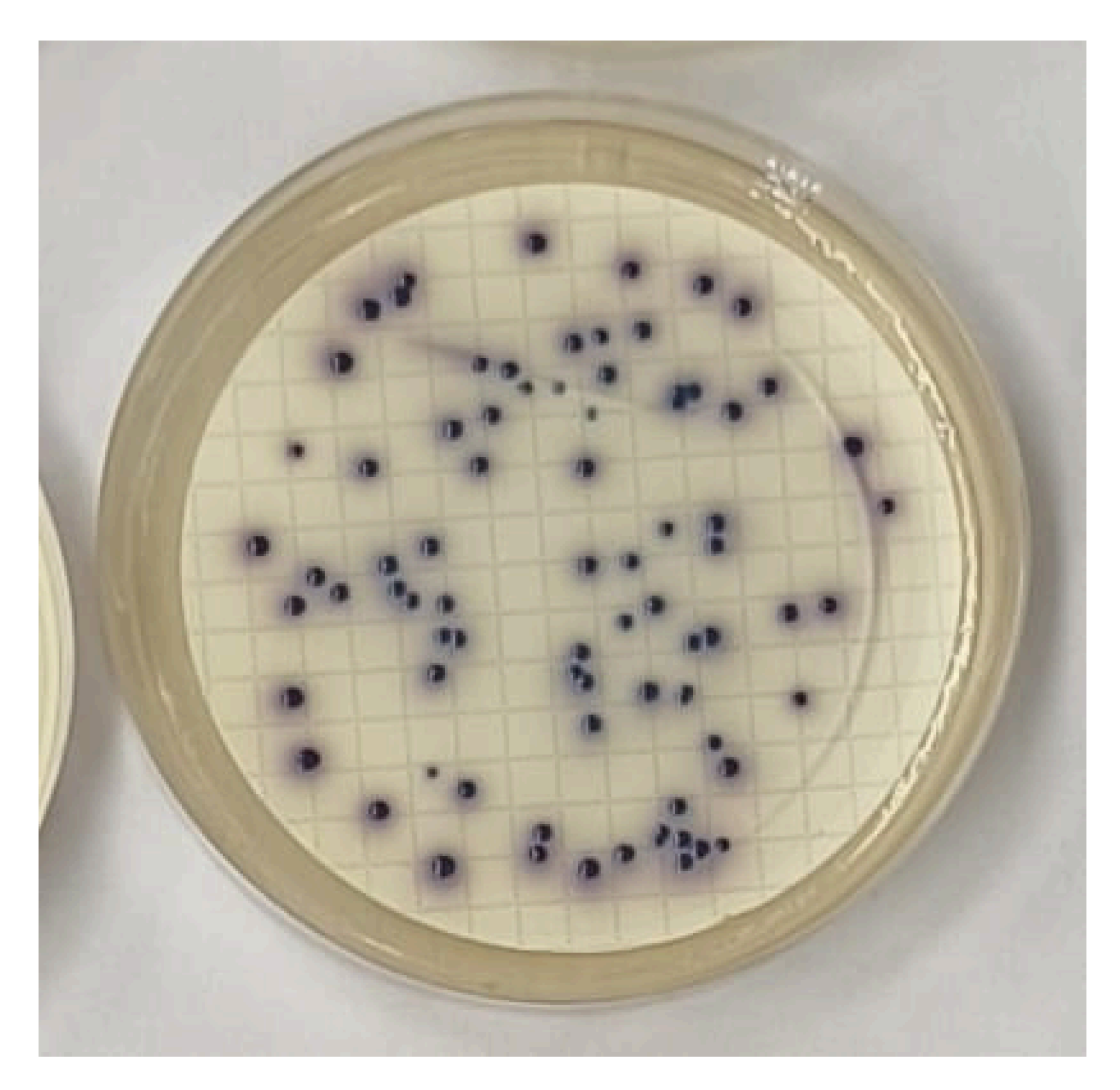	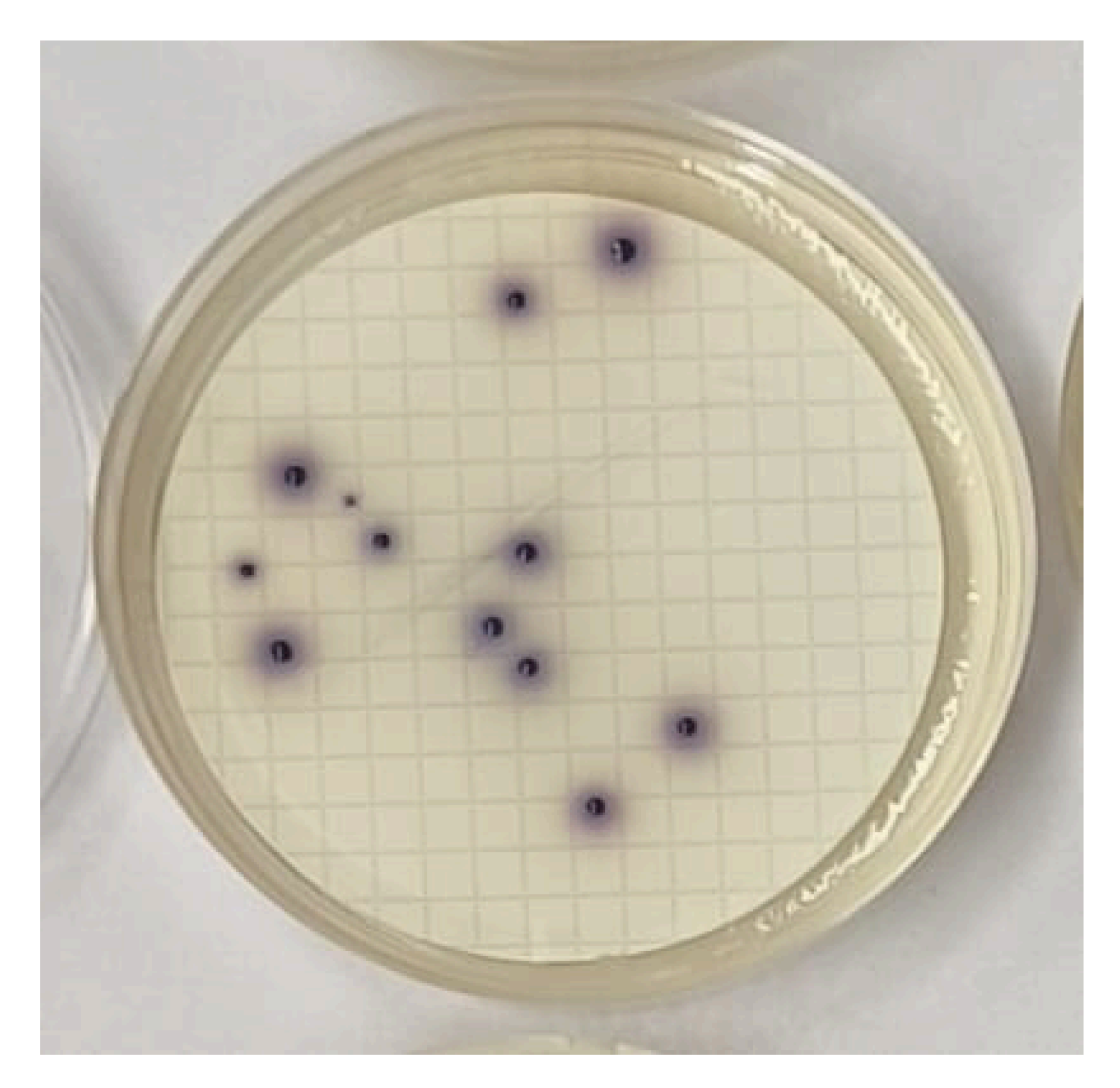

**Figure 7:**
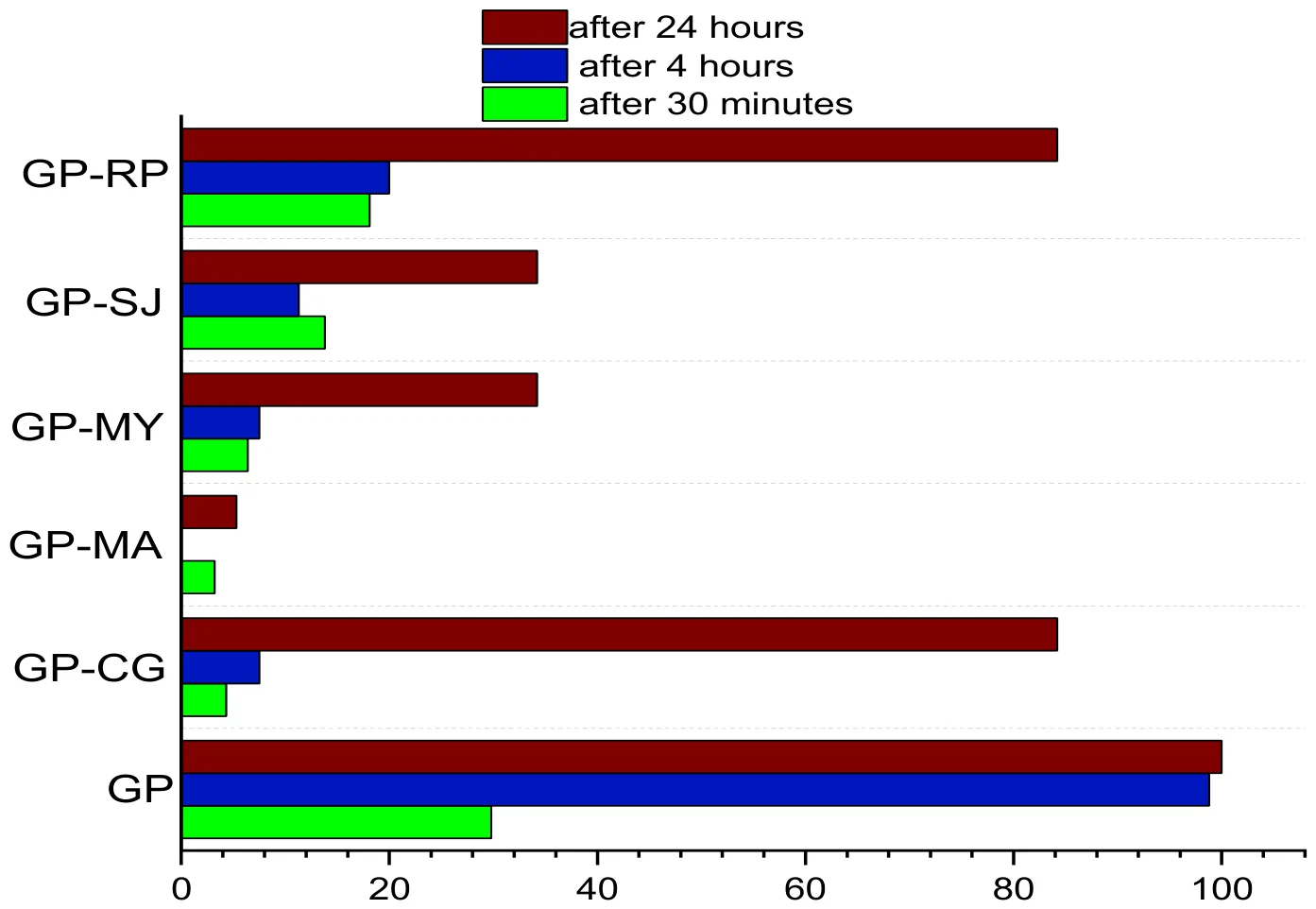
Variation in reduction efficiency of *E. coli*.

Reduction in recoverable *E. coli* colonies is presented in Table 7 and Figure 7.

**Table 7:** Results obtained in the *E. coli* test.

Samples	CFU/mL			Reduction Efficiency in Recoverable *E. coli* Colonies (%)		
	After 30 min	After 4 h	After 24 h	After 30 min	After 4 h	After 24 h
Control −	0	0	0	-	-	-
Control +	94	80	76	-	-	-
GP	66	1	0	29.8	98.8	100
GP-CG	90	74	12	4.3	7.5	84.2
GP-MA	91	88	72	3.2	0	5.3
GP-MY	88	74	50	6.4	7.5	34.2
GP-SJ	81	71	50	13.8	11.3	34.2
GP-RP	77	64	12	18.1	20	84.2

The reference geopolymer (GP) exhibited the highest reduction in recoverable *E. coli* colonies among all investigated materials. After only 30 min, the viable bacterial population decreased from 94 to 66 CFU mL^−1^, corresponding to an inhibition efficiency of approximately 30%. A dramatic reduction was observed after 4 h, when only 1 CFU mL^−1^ remained, representing an antibacterial efficiency exceeding 98%. Complete bacterial inhibition was achieved after 24 h, with no viable colonies detected.

The geopolymer modified with *Rapana venosa* Ca-based particles (GP-RP) showed the second-best reduction in recoverable *E. coli* colonies. Although only limited inhibition was observed during the first four hours, the bacterial population decreased markedly after 24 h, reaching 12 CFU mL^−1^, corresponding to approximately 84% inhibition.

A similar long-term behavior was observed for GP-CG, which exhibited only minor antibacterial activity during the initial contact period but achieved an inhibition efficiency comparable to GP-RP after 24 h.

In contrast, GP-MA displayed the weakest reduction in recoverable *E. coli* colonies. The bacterial population remained close to that of the positive control throughout the experiment, indicating that the incorporation of *Chamelea gallina* shell powder did not significantly enhance the reduction efficiency of the geopolymer matrix.

Intermediate behavior was observed for GP-MY and GP-SJ, both exhibiting approximately 34% bacterial reduction after 24 h, indicating moderate antibacterial activity.

The *E. coli* reduction efficiency increased with increasing contact time for all geopolymer composites, suggesting that bacterial inhibition is governed by a time-dependent interaction between the material surface and the bacterial cells rather than by an immediate bactericidal effect.

This behavior reflects the combined influence of mineralogical composition, pore architecture, and particle distribution. GP-CG and GP-RP exhibited the highest antibacterial efficiencies among the shell-modified geopolymers, which may be attributed to their favorable microstructural characteristics that promote intimate contact between bacterial cells and the geopolymer surface. Although GP-CG exhibited a lower BET surface area than GP-RP, its compact calcite-containing matrix appears to provide favorable surface chemistry for bacterial inactivation, indicating that preliminary antibacterial response is not governed solely by specific surface area. In contrast, GP-MA showed the lowest antibacterial efficiency, suggesting that its surface characteristics were less effective in promoting bacteria–surface interactions despite the presence of shell-derived calcium phases. GP-MY and GP-SJ displayed intermediate antibacterial activity, consistent with their intermediate microstructural and textural characteristics. These findings indicate that preliminary antibacterial response results from the synergistic effects of mineralogical composition, pore architecture, and surface properties rather than from the total specific surface area alone.

Interestingly, the incorporation of Ca-based particles from calcined shells did not systematically improve the antibacterial activity of the geopolymer matrix. Although several modified composites exhibited enhanced long-term inhibition, none surpassed the reduction in recoverable *E. coli* colonies of the reference geopolymer. This observation suggests that the antimicrobial behavior is controlled primarily by the intrinsic physicochemical properties of the geopolymer matrix, while the shell-derived additives influence the antibacterial response indirectly through modifications of the microstructure, surface characteristics, and porosity rather than acting as direct antimicrobial agents.

The present investigation was limited to *E. coli* and was conducted as a comparative screening study without independent biological replicates suitable for statistical analysis. Further work should include Gram-positive strains, replicated CFU assays, measurements of suspension pH and ion release, and post-contact surface characterization to validate the proposed antibacterial mechanisms.

### 3.7. Methylene Blue Adsorption Under Dark Condition

**Figure 8:**
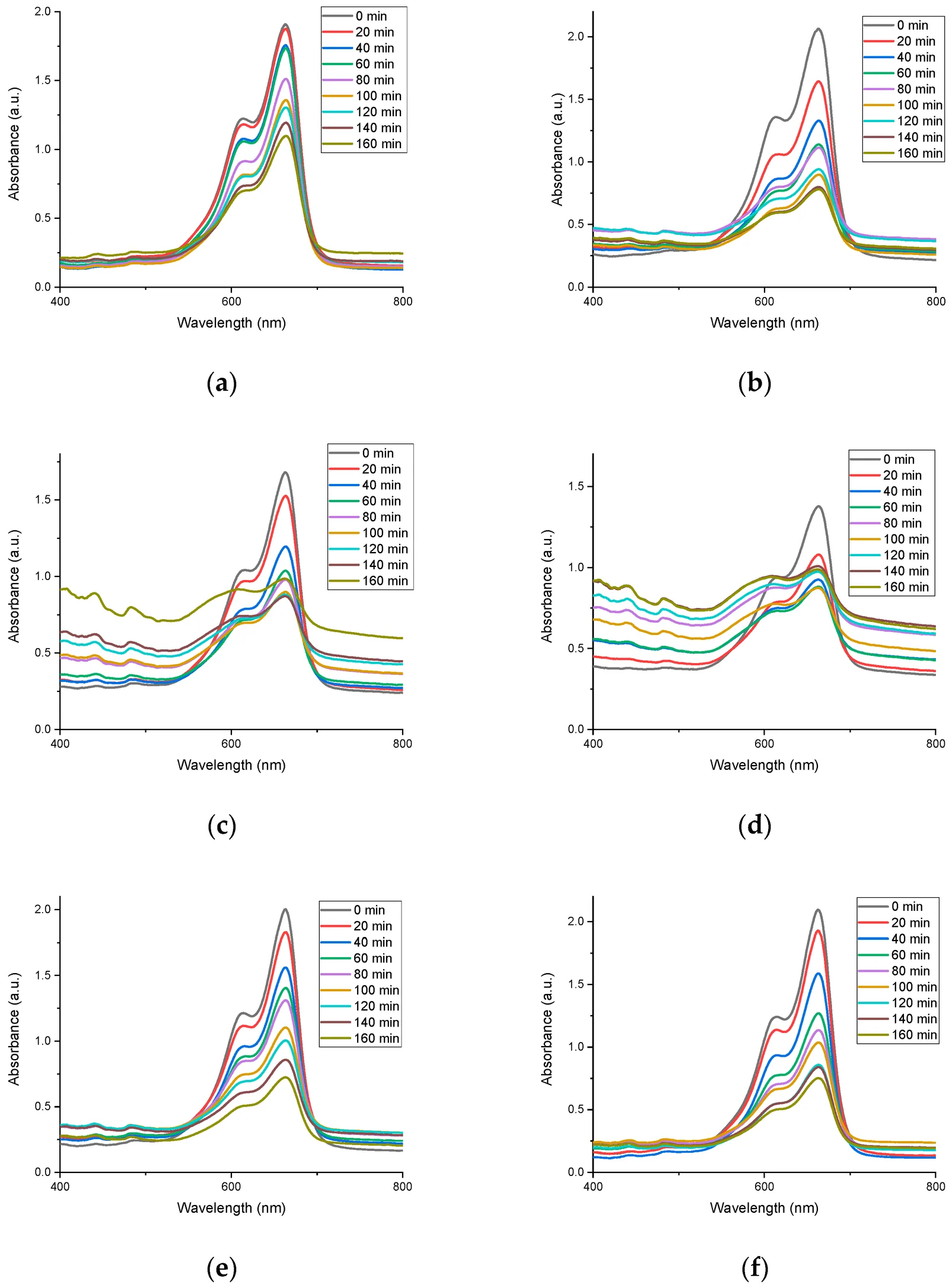
Time dependent UV-Vis adsorption spectra for MB dye solution: (**a**) GP; (**b**) GP-CG; (**c**) GP-MA; (**d**) GP-MY; (**e**) GP-SJ; (**f**) GP-RP.

The evolution of the UV–Vis spectra, presented in Figure 8, shows a gradual decrease in the characteristic methylene blue absorption band located at approximately 664–668 nm. Because the experiments were conducted in the dark to eliminate photocatalytic contributions, this decrease in MB concentration is consistent with adsorption of dye molecules onto the geopolymer surface.

All investigated samples exhibited a rapid adsorption stage during the first 60–80 min of contact, followed by a slower adsorption rate the absorbance profiles tended toward a plateau after approximately 140–160 min. This behavior is characteristic of adsorption processes in mesoporous materials, where the initially abundant active sites are progressively occupied, leading to a reduction in the adsorption rate as surface saturation is approached.

**Figure 9:**
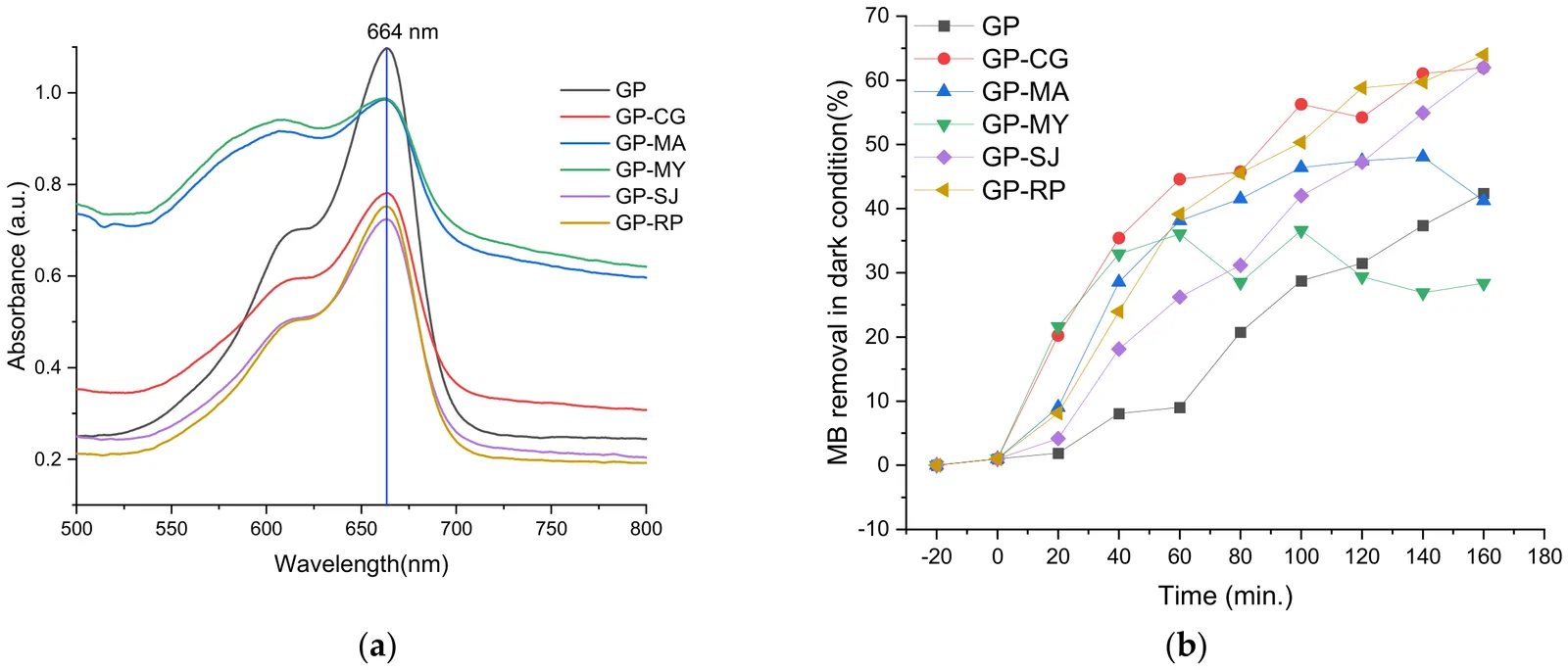
(**a**) UV–Vis absorption spectra of methylene blue solutions after 160 min; (**b**) MB removal in dark condition.

Figure 9a compares the UV–Vis absorption spectra of methylene blue solutions after 160 min of contact with the synthesized geopolymer samples. All spectra preserve the characteristic absorption maximum at approximately 664 nm, and no detectable shift in the principal MB absorption maximum was observed during the experiment. While this observation argues against major chemical degradation of the MB chromophore, it does not by itself exclude all alternative chemical or physical processes; therefore, the decrease in MB concentration under dark conditions is consistent with adsorption onto the geopolymer materials, rather than being definitively established by UV–Vis monitoring alone.

A pronounced reduction in the absorbance intensity at 664 nm is observed for all calcium-modified geopolymers compared with the initial solution, indicating their ability to remove methylene blue from the aqueous phase. However, significant differences are evident among the investigated geopolymers.

Among all samples, GP-RP exhibited the lowest absorbance over the entire visible region, demonstrating the highest adsorption efficiency and confirming the quantitative adsorption results obtained from the kinetic analysis. A very similar behavior was observed for GP-SJ while GP-CG showed an intermediate decrease in absorbance. In contrast, GP-MA and particularly GP-MY retained substantially higher absorbance values, indicating a larger residual dye concentration and consequently a lower adsorption capacity.

The differences become most evident at the characteristic MB absorption band centered at 664 nm, where the progressive decrease in peak intensity follows the order:

GP-RP≈GP-SJ>GP-CG>GP>GP-MA>GP-MY

The reference geopolymer (GP) reached an adsorption efficiency of 42.39% after 160 min. The incorporation of shell-derived calcium-rich particles modified the adsorption depending on the biological origin of the additive. The highest adsorption efficiencies were obtained for GP-RP (63.97%), GP-SJ (61.98%), and GP-CG (61.96%), corresponding to an improvement of approximately 20% points compared with the reference geopolymer. In contrast, GP-MA exhibited an adsorption efficiency (41.23%) comparable to that of GP, whereas GP-MY showed the lowest adsorption capacity (28.36%).

The adsorption capacity ($q_{160}$) was calculated using the experimentally determined methylene blue removal efficiencies after 160 min. As summarized in Table 8, the adsorption capacities ranged from 4.25 mg g^−1^ for GP-MY to 9.60 mg g^−1^ for GP-RP. GP-CG and GP-SJ exhibited comparable adsorption capacities of 9.29 and 9.30 mg g^−1^, respectively, whereas the reference geopolymer reached only 6.36 mg g^−1^. These results confirm that shell-derived calcium particles produced formulation-dependent adsorption behavior.

These differences can be directly correlated with the textural characteristics revealed by BET analysis. The reference geopolymer exhibited the lowest specific surface area (36.22 m^2^ g^−1^) and the lowest pore volume, resulting in a limited number of accessible adsorption sites. Conversely, GP-SJ and GP-CG displayed substantially higher BET surface areas (94.30 and 84.90 m^2^ g^−1^, respectively), together with larger pore volumes and well-developed mesoporous networks, providing a considerably larger accessible surface for dye adsorption. The enhanced adsorption observed for these composites therefore originates from the combined effect of increased specific surface area, improved pore connectivity, and higher accessibility of the adsorption sites.

Although GP-MA exhibited a relatively high BET surface area (63.78 m^2^ g^−1^), its adsorption performance remained similar to that of the reference geopolymer. This apparent discrepancy can be explained by the compact microstructure observed by SEM and by the lowest mesoporosity index among the modified samples, suggesting that the incorporation of calcite particles promoted matrix densification and partial blockage of the mesoporous network. Consequently, despite the increase in total surface area, the accessibility of adsorption sites was reduced, limiting the overall adsorption capacity.

GP-MY exhibited another distinctive behavior. Despite possessing a relatively high BET surface area (71.28 m^2^ g^−1^), it presented the lowest adsorption efficiency. This result indicates that adsorption is not governed solely by the magnitude of the specific surface area. The XRD analysis identified aragonite as the predominant calcium carbonate polymorph in GP-MY, unlike the calcite-rich GP-SJ and GP-RP composites. The different crystallographic structure of aragonite, together with its interaction with the aluminosilicate matrix, may reduce the affinity of the composite surface toward methylene blue molecules, leading to a lower adsorption efficiency despite the relatively developed porous structure.

The adsorption performance of GP-SJ and GP-RP can also be associated with their favorable microstructural organization. SEM observations revealed a well-developed porous architecture and good dispersion of the calcium-rich particles within the geopolymeric matrix, while BET analysis was consistent with the presence of highly accessible mesoporous networks. Such structures facilitate both the diffusion of dye molecules into the porous network and the availability of many adsorption sites. In contrast, GP-CG, although characterized by a more heterogeneous morphology, still exhibited high adsorption efficiency due to its large specific surface area and high pore volume, which compensated for the local structural heterogeneity.

The pseudo-first-order (PFO) adsorption kinetics were evaluated using the Lagergren model. Its linearized form is expressed as Equation (5).

(5) $$\ln\left(q_{e}-q_{t}\right)=\ln\left(q_{e}\right)-k_{1}\cdot t$$

where q_e_ and q_t_ (mg g^−1^) represent the adsorption capacities at equilibrium estimated and at time t, respectively, and k_1_ (min^−1^) is the PFO rate constant. For the linearized PFO analysis, the adsorption capacity measured at the end of the investigated contact period was used as an experimental approximation of when an unequivocal equilibrium plateau could not be established. Therefore, this value was considered an operational estimate rather than a definitive equilibrium adsorption capacity.

To avoid potential distortions associated with linearization, the PFO model was additionally fitted directly to the experimental data using its nonlinear form, as presented in Equation (6).

(6) $$q_{t}=q_{e}\cdot \left[1-e^{-k_{1}\cdot t}\right]$$

where both q_e_ and k_1_ were determined as fitted parameters.

The pseudo-second-order (PSO) model was also evaluated to provide an independent comparison of the kinetic behavior. The linearized Ho–McKay equation was expressed in Equation (7).

(7) $$\frac{t}{q_{t}}=\frac{1}{k_{2}\cdot q_{e}^{2}}+\frac{t}{q_{e}}$$

where k_2_ (g mg^−1^ min^−1^) is the PSO rate constant. The corresponding nonlinear PSO equation was:

For the nonlinear PFO and PSO analyses, the models were fitted directly to the experimental q_t_ values by unweighted nonlinear least-squares regression, with q_e_ and the corresponding kinetic rate constant (k_1_ for PFO and k_2_ for PSO) treated as adjustable parameters. The linearized models were evaluated by unweighted linear least-squares regression.

**Figure 10:**
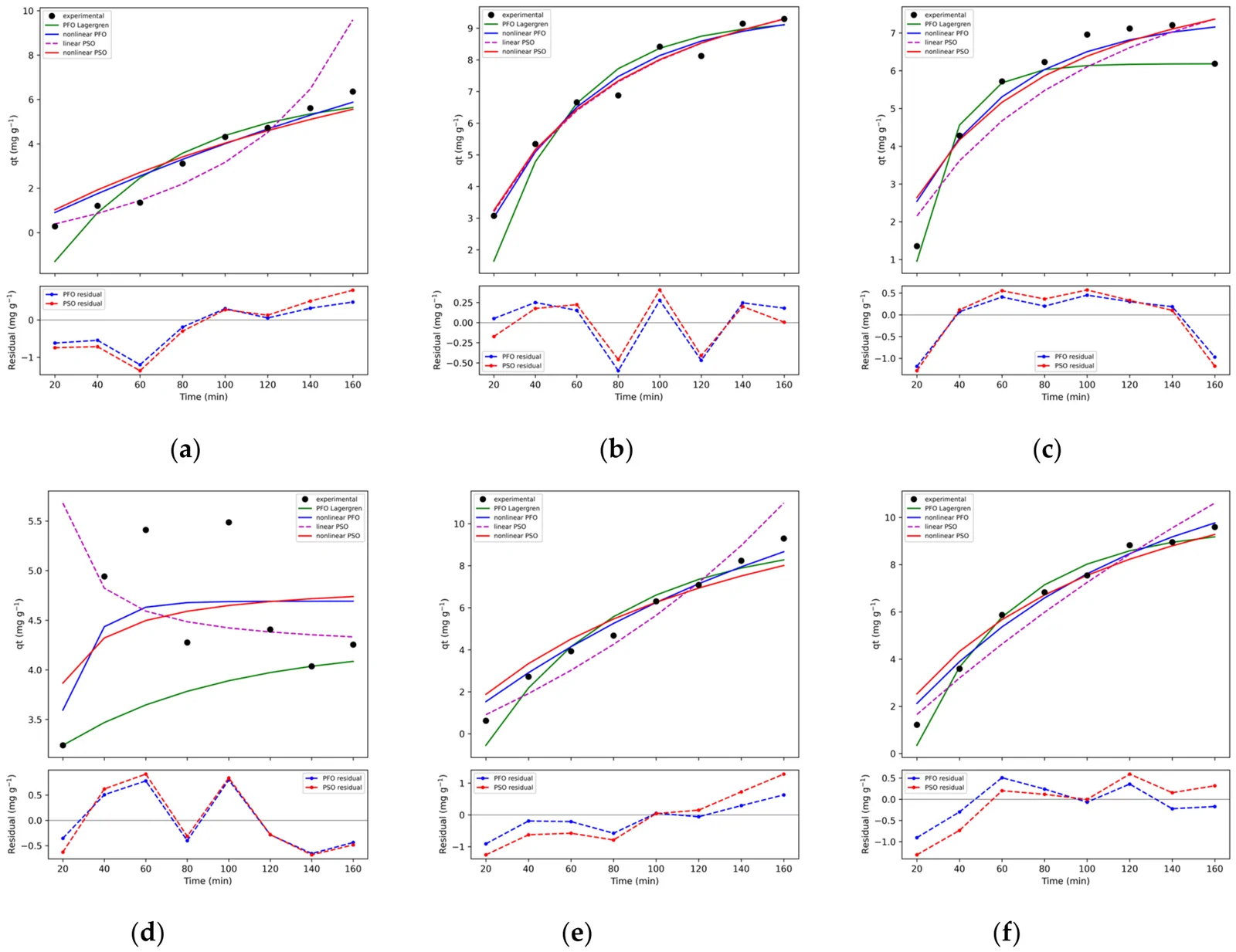
Comparison of pseudo-first-order (PFO) and pseudo-second-order (PSO) kinetic models for methylene blue adsorption onto the investigated geopolymers: (**a**) GP, (**b**) GP-CG, (**c**) GP-MA, (**d**) GP-MY, (**e**) GP-SJ, and (**f**) GP-RP, over the 20–160 min contact-time interval. Black symbols represent the experimental adsorption capacities (q_t_), while the curves correspond to the linearized Lagergren PFO, nonlinear PFO, linearized PSO, and nonlinear PSO model predictions. The lower panels show the residuals, defined as the differences between experimental and predicted values, for the nonlinear PFO and PSO fits.

Model performance was assessed using several complementary statistical criteria rather than relying exclusively on the coefficient of determination. The goodness of fit was evaluated using the coefficient of determination (R^2^), and Akaike information criterion (AIC). Agreement between experimentally observed and model-predicted adsorption capacities was also considered. In addition, residual plots were examined to identify systematic deviations between experimental and fitted values that may not be evident from alone. For direct comparison of competing kinetic models, goodness-of-fit statistics were calculated in the original response domain whenever applicable as presented in Figure 10, Table 9 and Table 10.

The nonlinear kinetic fits revealed sample-dependent adsorption behavior. For GP-CG, both models provided a satisfactory representation of the experimental data, with values of 0.973 and 0.978 for PFO and PSO, respectively. The slightly lower AIC obtained for PSO (−13.58) compared with PFO (−12.09) indicates that PSO provides a marginally better empirical description of MB uptake by GP-CG. The relatively small and alternating residuals further support the adequacy of both models for this sample.

GP-RP was also satisfactorily described by the nonlinear models; however, PFO provided the better statistical description, with R^2^ = 0.976 and AIC = −7.76, compared with R^2^ = 0.953 and AIC = −2.45 for PSO. The nonlinear PFO rate constant obtained for GP-RP was k_1_ = 0.00913 min^−1^. This value does not represent the highest nonlinear PFO rate constant among the investigated materials and therefore does not support designation of GP-RP as the fastest adsorbing material. Nevertheless, GP-RP reached the highest experimental adsorption capacity at 160 min (mg g^−1^), corresponding to the highest MB removal efficiency (63.97%). GP-SJ similarly showed a better agreement with PFO (R^2^ = 0.971, AIC = −6.26) than with PSO (R^2^ = 0.914, AIC = 2.44).

For GP-MA, the nonlinear PFO model performed better than the nonlinear PSO model, although neither model reproduced all experimental points with the same accuracy. This result is particularly relevant because the previously used apparent first-order representation yielded. The analysis therefore demonstrates that this low coefficient should not be interpreted as the goodness of fit of the conventional Lagergren PFO adsorption model.

GP-MY exhibited distinctly different behavior. Neither nonlinear PFO nor nonlinear PSO satisfactorily described the experimental adsorption profile. The pronounced non-monotonic variation suggests that a simple single-rate kinetic expression is insufficient to represent this system within the investigated time interval. Consequently, no mechanistic interpretation based on PFO or PSO fitting is proposed for GP-MY. The linearized Lagergren regression for GP-MY relied on only two valid points due to the non-monotonic q_t_ profile, and the resulting R^2^ = 1.000 is a statistical artifact rather than evidence of model adequacy.

The linearized PSO analysis additionally generated nonphysical parameter estimates for several samples, including negative equilibrium adsorption capacities or negative kinetic constants (Table 10). These mathematically unstable estimates are retained only to illustrate the limitations associated with model linearization and were not used for kinetic interpretation. Accordingly, the nonlinear PFO and PSO regressions, performed directly in the original domain, were considered the principal basis for kinetic-model comparison. The linearized PFO and PSO results are therefore reported only as supplementary comparative representations and are not used to infer adsorption mechanisms or to rank the kinetic performance of the investigated materials.

The fitted nonlinear PFO rate constants (k_1_) further demonstrate the formulation-dependent character of the adsorption kinetics, as presented in Table 9. Although GP-MY yielded the highest fitted nonlinear k1, this value should not be interpreted as evidence of the fastest adsorption kinetics because the corresponding nonlinear PFO fit was poor (R^2^ = 0.365), indicating that the model does not adequately represent its experimental adsorption profile. Among the remaining samples, GP-MA exhibited the highest nonlinear PFO k_1_ value; however, its model fit was less satisfactory (R^2^ = 0.894) than those obtained for GP-CG, GP-SJ, and GP-RP. Therefore, the kinetic rate constants were interpreted together with the corresponding goodness-of-fit parameters rather than as standalone indicators of adsorption performance. Importantly, GP-RP should not be regarded as the material with the fastest adsorption kinetics; instead, its principal experimentally demonstrated advantage was the highest adsorption capacity after 160 min (q_160_ = 9.60 mg g^−1^), corresponding to the highest MB removal efficiency (63.97%) under the investigated conditions.

The kinetic analysis does not support a universal kinetic model for all investigated geopolymers. PFO provided the statistically preferred description for GP, GP-MA, GP-SJ, and GP-RP, whereas PSO was marginally favored for GP-CG. Neither model adequately described GP-MY. Importantly, these kinetic models are considered empirical descriptions of the adsorption-rate profiles rather than direct evidence of a specific molecular adsorption mechanism. Thus, a better agreement with the PSO model, as observed for GP-CG, should not be interpreted as proof of chemisorption.

The results indicate that adsorption efficiency is associated with the combined influence of specific surface area, pore accessibility, mesoporous network development, and the mineralogical nature of the calcium-containing phases. While an increase in BET surface area generally promotes methylene blue adsorption, the results further demonstrate that pore accessibility and microstructural organization are equally important in determining the overall adsorption performance. Consequently, GP-SJ and GP-RP exhibited the most favorable combination of textural and structural properties, making them the most promising materials for dye adsorption applications.

## 4. Discussion

### 4.1. Effect of Shell Mineralogy on Geopolymerization

The comprehensive characterization performed in this study suggests that the incorporation of calcined marine shell powders into the metakaolin-based geopolymer matrix does not merely introduce calcium-rich mineral phases, but fundamentally modifies the structural organization, pore architecture and functional performance of the resulting composites. The combined interpretation of XRD, SEM, EDS, BET, methylene blue adsorption and antibacterial assays reveal that the type of shell-derived calcium source plays a decisive role in controlling the balance between matrix densification and mesoporous network development, ultimately determining the environmental performance of the geopolymers.

The mineralogical composition of the shell-derived particles also plays an important role during geopolymerization. XRD analysis confirmed that all modified geopolymers preserved the characteristic aluminosilicate framework of the geopolymer matrix, composed primarily of quartz, muscovite and iron oxide embedded within the amorphous N-(K)-A-S-H gel. The shell-derived additives introduced additional calcium carbonate phases, predominantly calcite in GP-CG, GP-MA, GP-SJ and GP-RP, while aragonite was identified only in GP-MY. The calcite-containing formulations generally exhibited more favorable pore architectures than the aragonite-containing GP-MY sample; however, this association should not be interpreted as evidence of a direct causal effect of carbonate polymorphism, since other shell-dependent compositional and microstructural factors may also contribute. Well-dispersed calcite particles appear to promote the formation of accessible mesoporosity, whereas excessive calcium accumulation, as observed for GP-MA, results in matrix densification and partial pore blocking. The presence of aragonite in GP-MY is associated with a less pronounced modification of the pore structure, leading to intermediate textural properties and lower adsorption performance.

The relatively constant Si/Al ratios determined by EDS further demonstrate that the geopolymerization process was not altered by the incorporation of shell powders. Instead, the calcium-rich particles acted primarily as textural modifiers, influencing the local organization of the geopolymer network rather than replacing the aluminosilicate gel.

The structural modifications observed by XRD are consistent with the microstructural evolution revealed by SEM and the textural properties obtained by BET analysis. The reference geopolymer exhibited the lowest specific surface area (36.22 m^2^ g^−1^) together with a compact and relatively homogeneous morphology, confirming that the geopolymer gel alone generates a dense matrix with limited pore accessibility. The addition of shell-derived particles generally increased both the specific surface area and pore volume; however, the magnitude of this improvement strongly depended on the mineralogical nature and dispersion of the calcium phases.

GP-SJ and GP-CG exhibited the most favorable structural characteristics. SEM images revealed rough and well-developed surfaces, while BET analysis demonstrated the highest specific surface areas and large mesoporous volumes, indicating that particles derived from *Pecten maximus* and *Chamelea gallina* promoted the formation of interconnected pore networks. In contrast, GP-MA, despite containing abundant calcite, exhibited a denser microstructure associated with the highest Ca/Si and Ca/Al ratios determined by EDX. This observation suggests that excessive local calcium enrichment promotes matrix densification and partial pore blocking, limiting the effective development of accessible mesoporosity. GP-MY showed an intermediate behavior, where the presence of aragonite resulted in moderate textural improvement, indicating that the polymorphic nature of calcium carbonate also contributes to the evolution of the geopolymer structure. GP-RP presented intermediate BET characteristics, suggesting a more compact incorporation of calcite particles while maintaining a relatively well-connected porous architecture.

These structural differences are directly reflected in the adsorption behavior toward methylene blue. The adsorption experiments demonstrated that the removal efficiency is not governed solely by the BET specific surface area but rather by the combined effects of pore accessibility, mesoporous connectivity and surface chemistry. Although GP-SJ exhibited the highest BET surface area, GP-RP achieved the highest methylene blue removal efficiency (63.97%). Similarly, GP-CG also exhibited high adsorption efficiency despite a slightly lower BET value than GP-SJ. These observations indicate that an increase in specific surface area alone is insufficient to maximize adsorption performance. Instead, the accessibility of adsorption sites, diffusion pathways within the mesoporous network and the interaction between methylene blue molecules and calcium-modified geopolymer surfaces collectively determine the adsorption process.

The textural properties obtained in the present study are consistent with the broad range reported for metakaolin-based geopolymers modified with calcium-rich biogenic wastes. Previous studies have shown that incorporating shell-derived fillers generally alters the pore network through a combination of particle packing effects, partial dissolution of calcium-containing phases, and changes in geopolymer gel formation. Reported BET specific surface areas for shell-containing geopolymers typically range from approximately 10 to 60 m^2^ g^−1^, depending on the precursor composition, shell treatment, activator chemistry, and curing conditions. The geopolymer developed with *Pecten maximus*-derived particles exhibited the highest specific surface area and pore volume among the investigated formulations, placing it at the upper end of the values commonly reported for calcium-modified geopolymers. These results indicate that the origin and mineralogical composition of the shell-derived additives influence not only the calcium supply but also the evolution of the porous geopolymeric network.

### 4.2. Adsorption Mechanism

The adsorption performance towards methylene blue is also comparable to that reported for many shell-based geopolymer adsorbents described in the literature. Previous investigations generally attribute the adsorption process to the combined effects of electrostatic interactions, mesoporous diffusion, and the abundance of negatively charged aluminosilicate surface sites [14,17]. In the present work, the high adsorption capacities observed for GP-SJ and GP-CG correlate well with their larger specific surface areas and higher mesoporous volumes, confirming that adsorption is governed predominantly by the accessibility of active adsorption sites rather than solely by the total calcium content. This observation agrees with previous reports indicating that well-developed mesoporosity plays a more significant role in dye adsorption than the simple incorporation of calcium-rich fillers.

The adsorption of methylene blue by the investigated geopolymers can be attributed to the combined contribution of surface interactions and intraparticle mass transfer. The aluminosilicate framework and surface hydroxyl groups of geopolymers may provide sites capable of interacting with cationic methylene blue molecules. Nevertheless, because zeta-potential measurements and pH-dependent adsorption experiments were not performed, the surface charge of the materials under the actual experimental conditions cannot be conclusively established. Similarly, the possible contributions of ion exchange, hydrogen bonding, and dipole interactions cannot be independently confirmed without ion-release measurements or spectroscopic characterization before and after adsorption.

The mesoporous network facilitates the diffusion of MB molecules toward accessible adsorption sites; however, adsorption performance is not controlled by BET surface area alone. Although GP-SJ exhibited the largest specific surface area, GP-RP achieved the highest adsorption capacity after 160 min, indicating that pore connectivity, surface accessibility, and the spatial distribution of shell-derived particles are more relevant than the total measured surface area. Well-dispersed calcite particles may promote accessible diffusion pathways and heterogeneous adsorption sites, whereas excessive calcium accumulation and matrix densification, as observed for GP-MA, can partially block pores and restrict dye transport. The lower performance of the aragonite-containing GP-MY further suggests that the mineralogical form and integration of CaCO_3_ within the geopolymer matrix influence the resulting pore architecture and adsorption behavior. Therefore, MB removal may occur through a coupled mechanism involving electrostatic attraction, ion exchange, surface interactions, and diffusion through the mesoporous geopolymer network.

The sample-dependent kinetic behavior identified through the nonlinear PFO/PSO comparison provides additional, though indirect, support for this mixed-mechanism picture.

Table 11 compares the adsorption performance of the shell-modified geopolymers developed in this study with representative geopolymer-based adsorbents reported in the literature. Maximum adsorption capacities ranging from approximately 8 to 43.48 mg g^−1^ have been reported for metakaolin-based geopolymer adsorbents. However, a direct ranking of the materials is not fully appropriate because the studies employed different initial dye concentrations, adsorbent dosages, pH values, contact times, geopolymer compositions, and procedures for determining adsorption capacity. In particular, the literature values generally represent maximum equilibrium capacities derived from multi-concentration adsorption isotherms, whereas the values reported in the present study correspond to the adsorption capacity measured after 160 min at a single initial MB concentration of 15 mg L^−1^. Under these comparatively low-concentration conditions, GP-RP achieved the highest adsorption capacity among the investigated shell-modified materials, reaching 9.60 mg g^−1^, together with a removal efficiency of 63.97%. Although this value should not be interpreted as the maximum adsorption capacity of the material, it falls within the range reported for some conventional metakaolin-based geopolymers, while recognizing that direct quantitative comparison is limited by the substantially different experimental conditions employed across studies.

Moreover, unlike most previously reported geopolymer adsorbents, the materials developed in the present work were evaluated for both dye adsorption and antibacterial activity, supporting their potential as multifunctional materials for wastewater treatment.

### 4.3. E. coli Colony Reduction Efficiency

The *E. coli* colony reduction efficiency of the investigated materials is likely governed by several interacting physicochemical effects rather than by a single bactericidal mechanism. The highly alkaline nature of the geopolymer matrix may increase the local pH at the material–solution interface, potentially disturbing membrane integrity, enzyme activity, and cellular homeostasis in *E. coli*. In addition, dissolved alkali ions and changes in ionic strength may contribute to osmotic stress, while direct contact between bacterial cells and the heterogeneous geopolymer surface may promote membrane damage. Shell-derived calcium phases may also release limited amounts of Ca-containing species; however, the present results do not indicate a direct relationship between calcium incorporation and antibacterial efficiency. Indeed, none of the modified formulations consistently outperformed the reference geopolymer, suggesting that the shell-derived particles act mainly by modifying surface accessibility, porosity, compactness, and local chemical heterogeneity rather than as independent antimicrobial agents. Consequently, the observed antibacterial response is attributed primarily to the intrinsic alkalinity and surface characteristics of the geopolymer matrix, with an indirect contribution from the microstructural modifications induced by the biogenic calcium additives.

However, because the suspension pH during bacterial contact was not measured, the relative contribution of the intrinsic matrix alkalinity versus the shell-derived calcium phases to this antibacterial response cannot be unambiguously distinguished; this limitation is particularly relevant given that the unmodified reference geopolymer (GP) achieved the highest antibacterial efficiency, with complete bacterial inhibition (100%) after 24 h, exceeding all shell-modified formulations.

The *E. coli* colony reduction efficiency does not simply increase with calcium content or BET surface area but appears to depend on the accessibility of the geopolymer surface and the interaction between bacterial cells and the modified aluminosilicate matrix. The reference geopolymer exhibited the highest antibacterial activity against *E. coli*, indicating that the intrinsic alkaline geopolymer matrix possesses considerable antimicrobial properties. However, among the modified materials, GP-RP maintained one of the highest antibacterial efficiencies while simultaneously exhibiting the best adsorption performance. This behavior suggests that the balanced incorporation of calcite-derived particles preserves the antibacterial activity of the geopolymer while improving adsorption capability. In contrast, GP-CG, despite its highly developed porous structure and excellent adsorption performance, exhibited a comparatively lower antibacterial activity, indicating that the mechanisms governing dye adsorption and bacterial inactivation are not identical.

Similar antibacterial behavior has previously been reported for alkaline geopolymers and calcium-modified aluminosilicate matrices, where microbial inactivation is generally associated with the combined effects of alkaline surface conditions, ionic exchange, osmotic stress, and direct interactions between bacterial membranes and reactive mineral surfaces [5,8,20]. Unlike studies relying on metal nanoparticles such as Ag, Cu, or ZnO [1,2,3], the present materials achieve antibacterial activity without incorporating additional antimicrobial agents, demonstrating that biogenic calcium-based shell particles can contribute to multifunctional behavior while maintaining a relatively simple and environmentally friendly formulation.

Compared with previously published shell-derived geopolymers, most investigations have primarily focused on improving mechanical properties or recycling marine waste into construction materials [3,8,10,17]. Only a limited number of studies have simultaneously evaluated pore structure, adsorption capability, and antibacterial performance within the same material system [9,10,12]. Therefore, the present work extends the current state of the art by demonstrating that the biological origin of calcined shell particles affects the microstructure of the geopolymer matrix, which in turn governs both adsorption efficiency and antibacterial behavior.

The combined characterization results demonstrate that the functional performance of the investigated geopolymers cannot be explained by a single structural parameter.

These observations indicate that shell-derived particles do not simply act as inert mineral fillers. Instead, they influence the evolution of the geopolymer matrix by modifying local calcium availability, particle dispersion, and pore development during geopolymerization. The resulting balance between porosity, pore accessibility, and matrix compactness ultimately appears to influence the adsorption and reduction efficiency of recoverable *E. coli* colonies.

## 5. Conclusions

This study demonstrated that calcined marine shell waste can be successfully valorized as a functional calcium-rich additive for the development of multifunctional metakaolin-based geopolymers intended for environmental remediation applications. The incorporation of shell-derived particles modified the porous architecture of the geopolymer matrix without disrupting its aluminosilicate framework, producing hybrid materials with improved adsorption and antibacterial properties. The integrated analysis therefore indicates that multifunctional performance cannot be predicted from a single structural parameter, as presented in Table 12. Neither the highest BET surface area nor the highest calcium content alone guarantees optimum environmental performance. Instead, the results indicate that the best functional behavior is achieved through a balanced combination of mineralogical composition, particle dispersion, pore accessibility and matrix compactness. Well-dispersed calcite particles promote the development of interconnected mesoporosity and efficient mass transport, whereas excessive local calcium accumulation favors matrix densification and restricts pore accessibility. Likewise, the presence of aragonite appears to produce a less effective structural modification than calcite under the investigated synthesis conditions.

By controlling the evolution of pore architecture and surface accessibility, they simultaneously influence adsorption kinetics and antibacterial behavior. Among the investigated formulations, Under the investigated conditions, GP-RP exhibited the highest methylene blue removal efficiency and rapid adsorption kinetics while maintaining a comparatively high reduction in recoverable *E. coli* colonies during preliminary antibacterial screening. These findings demonstrate that the design of efficient geopolymer adsorbents should be based on the optimization of the entire microstructural architecture instead of maximizing individual parameters such as calcium content or BET specific surface area. Consequently, GP-RP emerges as the most promising material for sustainable wastewater depollution, enabling the simultaneous removal of organic dye pollutants and reduction in bacterial contamination using a low-cost geopolymer synthesized from biogenic shell waste.

The combined structural characterization suggests that the functional performance of the developed geopolymers is governed by the synergistic interaction between mineralogical composition, calcium distribution, pore accessibility and mesoporous network connectivity rather than by a single structural parameter such as calcium content or BET specific surface area. The results demonstrate that shell-derived calcium particles primarily act as microstructural regulators, controlling the evolution of the porous network and the accessibility of active adsorption sites.

The adsorption experiments revealed a strongly formulation-dependent effect of shell-derived calcium modification on methylene blue removal. GP-CG, GP-SJ, and GP-RP exhibited higher MB removal efficiencies (61.96%, 61.98%, and 63.97%, respectively) than the reference GP (42.39%), whereas GP-MA showed a slightly lower removal efficiency (41.23%) and GP-MY exhibited lower performance (28.36%) under the investigated conditions. Although GP-SJ exhibited the highest specific surface area, GP-RP achieved the highest experimental adsorption capacity at 160 min (9.60 mg g^−1^) and the highest MB removal efficiency (63.97%). The nonlinear PFO model also provided a satisfactory description of the GP-RP adsorption profile (R^2^ = 0.976, AIC = −7.76).

These results demonstrate that the incorporation of shell-derived calcium particles does not inherently improve adsorption performance; rather, the resulting behavior depends on the specific mineralogical and microstructural modifications induced by each shell-derived additive, including pore accessibility, particle dispersion, and matrix organization. In parallel, the antibacterial investigation showed that the incorporation of shell-derived particles influences bacterial inhibition differently from dye adsorption, indicating that these two functionalities may involve distinct physicochemical mechanisms.

Among the investigated formulations, GP-RP showed the most favorable overall combination of MB removal and preliminary antibacterial response under the investigated conditions. This assessment is based primarily on its highest experimentally measured MB removal efficiency and adsorption capacity at 160 min, together with a comparatively high reduction in recoverable *E. coli* colonies during preliminary antibacterial screening. However, because the antibacterial experiments were conducted without independent biological replicates, this overall assessment should be regarded as a qualitative comparison of the investigated functionalities rather than evidence of statistically demonstrated superiority or formal optimization. This overall performance indicates that multifunctional behavior is associated with a balance between shell-derived calcium incorporation, pore accessibility, and matrix organization rather than with the maximization of either porosity or calcium content alone. The results therefore provide useful structure–property guidelines for the development of multifunctional geopolymer adsorbents based on biogenic waste.

The present results indicate that no single structural parameter can adequately explain the adsorption and reduction in recoverable *E. coli* colonies of the investigated geopolymers. The highest calcium content did not correspond to the highest antibacterial activity or adsorption performance. These observations demonstrate that the functional behavior of the investigated materials results from the combined influence of mineralogical composition, calcium distribution, pore accessibility, mesoporous connectivity, and matrix compactness rather than from an individual structural characteristic.

This work highlights the potential of marine shell waste as a sustainable secondary raw material for producing shell-modified geopolymer composite with environmental functionality. The proposed approach simultaneously contributes to waste valorization, circular economy strategies and the development of low-cost materials capable of removing organic dye pollutants while reducing bacterial contamination in aqueous systems. These findings provide a solid basis for the future design of multifunctional geopolymer materials for sustainable wastewater depollution. Although methylene blue is widely used as a model cationic dye for evaluating the adsorption performance of novel materials, real industrial wastewater contains more complex chemical compositions, including mixtures of organic pollutants, dissolved salts, suspended solids, and competing inorganic ions. Such species may compete with dye molecules for the available adsorption sites or modify the surface charge of the geopolymer, potentially affecting adsorption efficiency. Likewise, solution pH is expected to influence both the surface chemistry of the geopolymer and the ionization state of the adsorbate, thereby altering electrostatic interactions and adsorption capacity. Further investigations involving real industrial effluents, regeneration and reuse cycles, pH-dependent adsorption, competing ions, and long-term durability are required before large-scale implementation can be considered. These investigations will contribute to the development of sustainable geopolymer-based technologies capable of simultaneously removing organic pollutants and reducing microbial contamination in water treatment systems.

## Figures and Tables

**Table 1:** BET variation.

Sample	BET (m^2^/g)	Single Point (m^2^/g)	t-Plot Extern (m^2^/g)	BJH ads. (m^2^/g)	BJH ads. Average Pore Diameter (nm)	BJH des. Average Pore Diameter (nm)
GP	36.22	35.41	33.51	31.74	35.20	31.78
GP-CG	84.90	83.11	77.05	78.93	17.24	14.98
GP-MA	63.78	62.36	59.36	55.40	15.80	13.41
GP-MY	71.28	69.63	67.22	68.86	12.10	11.24
GP-SJ	94.30	91.97	89.49	90.10	17.18	15.42
GP-RP	66.69	65.13	63.19	65.42	14.13	11.49

**Table 2:** Volume of pores.

Sample	Vp ads. at P/Po = 0.95 (cm^3^/g)	Vp des. at P/Po = 0.95 (cm^3^/g)	BJH ads. (cm^3^/g)	BJH des. (cm^3^/g)
GP	0.0939	0.1500	0.2794	0.2942
GP-CG	0.2312	0.2645	0.3403	0.3605
GP-MA	0.1444	0.1648	0.1677	0.1689
GP-MY	0.1841	0.2033	0.2720	0.2757
GP-RP	0.1568	0.1751	0.2312	0.2394
GP-SJ	0.2637	0.2946	0.3870	0.4085

**Table 3:** BET-JJH and IM.

Sample	BET (m^2^/g)	BJH ads. Pore Volume (cm^3^/g)	Mesoporosity Index (I_m_)
GP	36.22	0.2794	0.00771
GP-CG	84.90	0.3403	0.00401
GP-MA	63.78	0.1677	0.00263
GP-MY	71.28	0.2720	0.00382
GP-RP	66.69	0.2312	0.00347
GP-SJ	94.30	0.3870	0.00410

**Table 4:** Elemental composition of biogenic calcium-modified geopolymers.

Element		O	Si	Mg	Al	C	Na	Ca	K	Fe
		Wt.(%)								
**GP-CG**	zone 1	46.8	20.7	-	12.5	6.5	6.3	3.6	3.3	0.3
	zone 2	50.7	15.6	-	10	14	6.2	1.5	1.9	0.2
**GP-MA**	zone 1	53	18	-	11.3	7.6	5.6	2.2	2.1	0.2
	zone 2	45.5	20.5	-	12.2	5.9	7.8	4.1	3.6	0.4
**GP-MY**	zone 1	51.7	18.5	-	11.4	7.1	7	2.2	2	0.2
	zone 2	47.7	21.7	-	13.1	6.5	5	3.1	2.7	0.2
**GP-SJ**	zone 1	50.2	19.6	-	12.2	6.8	6	2.5	2.4	0.2
	zone 2	48.6	20.3	-	12.4	6.6	6.2	2.9	2.7	0.2
**GP-RP**	zone 1	49.5	21.1	0.2	13.2	4.5	6.3	2.2	2.7	0.3
	zone 2	46.3	21.5	0	13	5.6	5.9	3.8	3.6	0.3

**Table 5:** Calculated ratio of biogenic calcium-modified geopolymers.

Sample	Si/Al	Na/Al	(Na+K)/Al	Ca/Si	Ca/Al	Ca/(Si+Al)
**GP-CG**	1.61	0.56	0.79	0.14	0.22	0.08
**GP-MA**	1.64	0.58	0.82	0.16	0.27	0.10
**GP-MY**	1.64	0.56	0.77	0.12	0.19	0.07
**GP-SJ**	1.62	0.55	0.76	0.13	0.21	0.08
**GP-RP**	1.63	0.47	0.71	0.14	0.23	0.09

**Table 8:** Methylene blue removal efficiency and adsorption capacity after 160 min.

Sample	Removal Efficiency (%)	Adsorption Capacity q_160_ (mg g^−1^)
**GP**	42.39	6.36
**GP-CG**	61.96	9.29
**GP-MA**	41.23	6.18
**GP-MY**	28.36	4.25
**GP-SJ**	61.98	9.3
**GP-RP**	63.97	9.6

**Table 9:** Kinetic parameters of Lagergren and PFO models.

Sample	q_160_, exp (mg g^−1^)	R^2^ PFO Lagergren	k_1_ Lagergren (min^−1^)	qe, Lagergren Intercept (mg g^−1^)	R^2^ PFO Nonlinear	q_e,_ PFO Nonlinear (mg g^−1^)	k_1_ PFO Nonlinear (min^−1)^
GP	6.36	0.926	0.0169	10.736	0.926	15	0.00311
GP-CG	9.29	0.843	0.02639	12.968	0.973	9.573	0.01897
GP-MA	6.18	0.986	0.05842	16.811	0.894	7.415	0.02098
GP-MY	4.25	1	0.01281	1.311	0.365	4.692	0.07265
GP-SJ	9.30	0.942	0.0162	13.609	0.971	15	0.00539
GP-RP	9.60	0.97	0.02215	14.396	0.976	12.717	0.00913

q160, exp, represents experimental adsorption capacity measured at 160 min; k_1_ is the PFO rate constant; qe, PFO linear is equilibrium capacity from the linearized Lagergren PFO intercept; q_e_, PFO nonlinear is equilibrium capacity from the nonlinear PFO fit.

**Table 10:** Linearized PSO parameters and nonlinear PSO fitting.

Sample	R^2^ PSO Linear	q_e_, PSO Linear	k_2_ PSO Linear (g mg^−1^ min^−1^)	R^2^ PSO Nonlinear	q_e_, PSO Nonlinear (mg g^−1^)	AIC PFO Nonlinear	AIC PSO Nonlinear
GP	0.558	−4.0348 *	0.00109	0.886	15	−3.03	0.46
GP-CG	0.983	12.766	0.00131	0.978	12.652	−12.09	−13.58
GP-MA	0.671	11.288	0.00104	0.855	9.892	−2.14	0.34
GP-MY	0.962	4.19	−0.04548 *	0.186	4.896	−3.34	−1.35
GP-SJ	0.21	−18.937 *	0.00012	0.914	15	−6.26	2.44
GP-RP	0.182	46.529	4e-05	0.953	15	−7.76	−2.45

k_2_ is the PSO rate constant, R^2^ is the coefficient of determination; q_e_, PSO linear is equilibrium capacity from the linearized PSO; q_e_, PSO nonlinear is equilibrium capacity from the nonlinear PSO fit, and AIC is the Akaike information criterion.* Nonphysical parameter estimate resulting from the linearized PSO regression. These values are reported only to document the limitations of the linearized representation and were not used for kinetic interpretation. Nonlinear PFO and PSO regression was used as the principal basis for kinetic-model comparison.

**Table 11:** Comparison of the MB adsorption performance of representative geopolymer-based adsorbents reported in the literature.

Graph 2.	Main Precursor	BET (m^2^ g^−1^)	MB Adsorption Condition	Adsorption Capacity (mg g^−1^)	MB Removal Efficiency (%)	Best-Fit Isotherm/Kinetic Model	Antibacterial Performance	Reference
MK-based geopolymer	MK	N.R.	C_0_ = 40 mg L^−1^; 0.1 g adsorbent/100 mL; 120 min.	q_max_ = 43.48	N.R.	Langmuir; pseudo-second order	N.R.	[28]
Foamed metakaolin-based geopolymer	Metakaolin-based geopolymer foam	N.R.; mesopore volume: 0.143 cm^3^ g^−1^	Batch and fixed-bed experiments; removal evaluated up to $C_{0}=25$ mg L^−1^	q_max_ = 40.0	≈95	Langmuir; pseudo-second order	N.R.	[29]
Metakaolin-based geopolymers with different Si/Al ratios	Four geopolymer formulations obtained by varying the Si/Al ratio	N.R.	T = 30 °C; pH 7; equilibrium reached within approximately 5–10 min	19.0–35.3	N.R.	Langmuir; adsorption dynamic intraparticle model	N.R.	[30]
Partially dealuminated metakaolin-based geopolymer	Partially dealuminated metakaolin activated using 12 M NaOH	9.3	C_0_ = 60 mg L^−1^; 0.35 g adsorbent; pH 7–12; 240 min	q_max_ = 8.0	99	Freundlich; pseudo-second order	N.R.	[31]

N.R. = not reported; q_max_ represents the maximum adsorption capacity obtained from equilibrium/isotherm experiments. These values should not be interpreted as directly equivalent.

**Table 12:** Multifunctional performance of biogenic Ca-modified GP.

Sample	XRD	SEM	BET	EDX	Interpretation
GP	aluminosilicate	compact	low	low Ca	dense matrix
GP-CG	calcite	porous	high	moderate Ca	interconnected mesopores
GP-MA	calcite	compact	moderate	highest Ca	pore blocking
GP-MY	aragonite	intermediate	intermediate	low Ca	limited textural modification
GP-SJ	calcite	rough	highest	moderate Ca	optimal pore development
GP-RP	calcite	heterogeneous	intermediate	moderate Ca	balanced compactness

## Data Availability

The original contributions presented in this study are included in the article/Supplementary Material. Further inquiries can be directed to the corresponding author.

## Supplementary Materials

The following supporting information can be downloaded at: https://www.mdpi.com/article/10.3390/polym18162005/s1, Figure S1: EDX spectra and corresponding elemental mapping images of the investigated shell-modified geopolymers: (a,b) GP-CG; (c,d) GP-MA; (e,f) GP-MY; (g,h) GP-SJ; and (i,j) GP-RP. For each sample, the first panel shows the EDX spectrum acquired from area 2, while the second panel presents the corresponding elemental distribution maps.
