# Supplementary material for: Structure–Property Relationships in Metakaolin Geopolymers Modified with Shell-Derived Calcium Particles for Multifunctional Wastewater Treatment
Source: Polymers (Basel). 2026 Aug 17;18(16):2005. doi: 10.3390/polym18162005 (PMC13516835; doi:10.3390/polym18162005)
Supplement: Supplementary file 1 [file polymers-18-02005-s001.zip › polymers-4471514-supplementary.pdf]

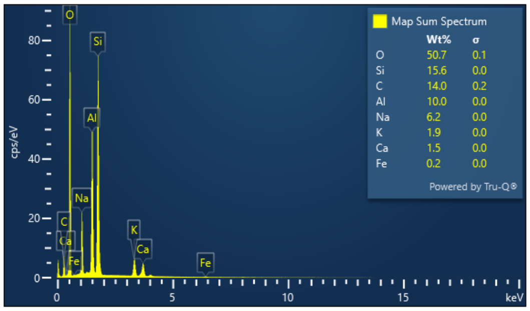
Map Sum Spectrum
Wt% σ
O 50.7 0.1
Si 15.6 0.0
C 14.0 0.2
Al 10.0 0.0
Na 6.2 0.0
K 1.9 0.0
Ca 1.5 0.0
Fe 0.2 0.0
Powered by Tru-Q®
cps/eV
keV

(a)

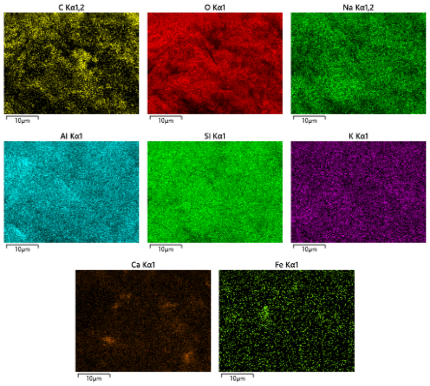
C Kα1,2
O Kα1
Na Kα1,2
Al Kα1
Si Kα1
K Kα1
Ca Kα1
Fe Kα1

(b)

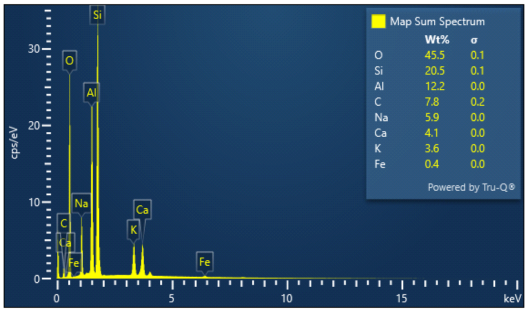
Map Sum Spectrum
Wt% σ
O 45.5 0.1
Si 20.5 0.1
Al 12.2 0.0
C 7.8 0.2
Na 5.9 0.0
Ca 4.1 0.0
K 3.6 0.0
Fe 0.4 0.0
Powered by Tru-Q®
cps/eV
keV

(c)

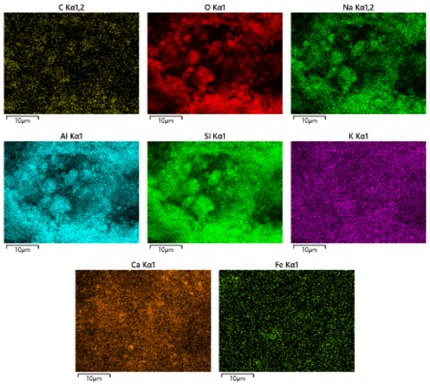
C Kα1,2
O Kα1
Na Kα1,2
Al Kα1
Si Kα1
K Kα1
Ca Kα1
Fe Kα1

(d)

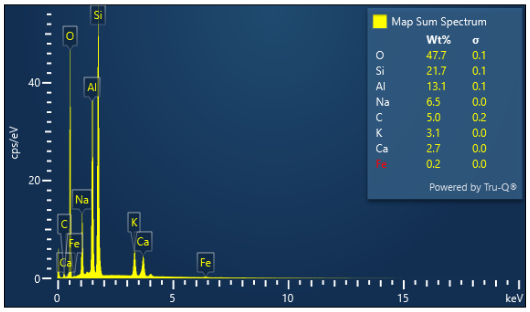
Map Sum Spectrum
Wt% σ
O 47.7 0.1
Si 21.7 0.1
Al 13.1 0.1
Na 6.5 0.0
C 5.0 0.2
K 3.1 0.0
Ca 2.7 0.0
Fe 0.2 0.0
Powered by Tru-Q®
cps/eV
keV

(e)

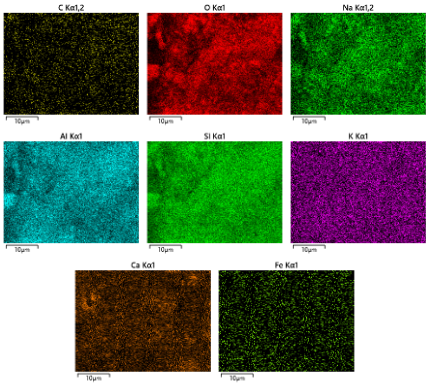
C Kα1,2
O Kα1
Na Kα1,2
Al Kα1
Si Kα1
K Kα1
Ca Kα1
Fe Kα1

(f)

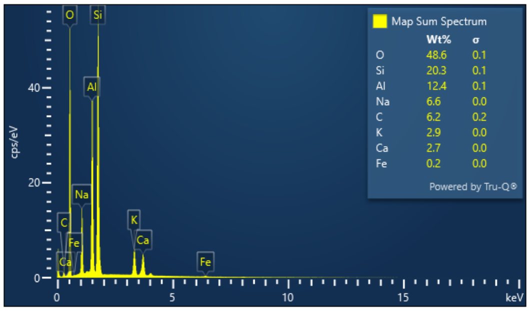

(g)

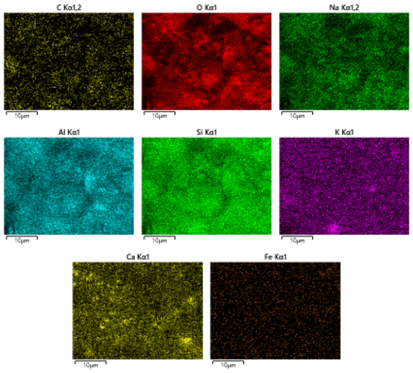

(h)

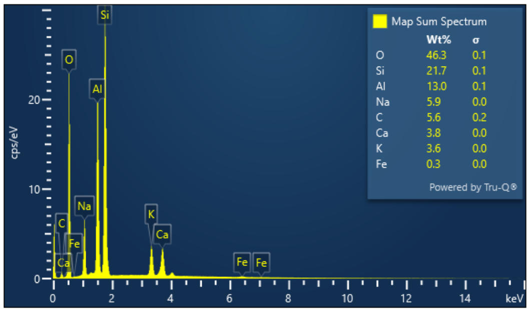

(i)

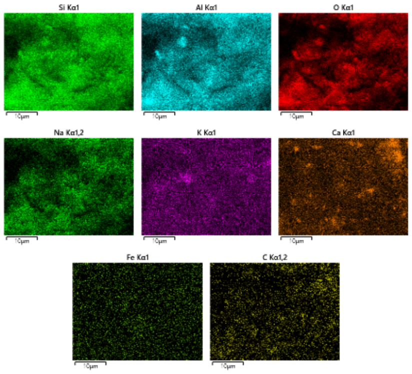

(j)

Figure S1. EDX spectra and corresponding elemental mapping images of the investigated shell-modified geopolymers: (a,b) GP-CG; (c,d) GP-MA; (e,f) GP-MY; (g,h) GP-SJ; and (i,j) GP-RP. For each sample, the first panel shows the EDX spectrum acquired from area 2, while the second panel presents the corresponding elemental distribution maps.
